# Genomics-Driven Activation of Silent Biosynthetic Gene Clusters in *Burkholderia gladioli* by Screening Recombineering System

**DOI:** 10.3390/molecules26030700

**Published:** 2021-01-29

**Authors:** Hanna Chen, Tao Sun, Xianping Bai, Jie Yang, Fu Yan, Lei Yu, Qiang Tu, Aiying Li, Yajie Tang, Youming Zhang, Xiaoying Bian, Haibo Zhou

**Affiliations:** 1 Helmholtz International Lab for Anti-Infectives, Shandong University–Helmholtz Institute of Biotechnology, State Key Laboratory of Microbial Technology, Shandong University, Qingdao 266237, China; chenhannahappy@163.com (H.C.); suntao_go@126.com (T.S.); xpbai201812479@163.com (X.B.); yangjie0737@163.com (J.Y.); fuyan@sdu.edu.cn (F.Y.); tuqiang1986@163.com (Q.T.); ayli@sdu.edu.cn (A.L.); yajietang@sdu.edu.cn (Y.T.); 2 Jiangsu Marine Resources Development Research Institute, Lianyungang 222005, China; 3 Soil and Fertilizer Station of Shandong Province, Jinan 250100, China; yuleisdnu@163.com

**Keywords:** *Burkholderia*, recombinase system, lipopeptides, promoter engineering, silent biosynthetic gene clusters, genome mining

## Abstract

The *Burkholderia* genus possesses ecological and metabolic diversities. A large number of silent biosynthetic gene clusters (BGCs) in the *Burkholderia* genome remain uncharacterized and represent a promising resource for new natural product discovery. However, exploitation of the metabolomic potential of *Burkholderia* is limited by the absence of efficient genetic manipulation tools. Here, we screened a bacteriophage recombinase system Redγ-BAS, which was functional for genome modification in the plant pathogen *Burkholderia gladioli* ATCC 10248. By using this recombineering tool, the constitutive promoters were precisely inserted in the genome, leading to activation of two silent nonribosomal peptide synthetase gene clusters (*bgdd* and *hgdd*) and production of corresponding new classes of lipopeptides, burriogladiodins A–H (**1**–**8**) and haereogladiodins A–B (**9**–**10**). Structure elucidation revealed an unnatural amino acid *Z*- dehydrobutyrine (Dhb) in **1**–**8** and an *E*-Dhb in **9**–**10**. Notably, compounds **2**–**4** and **9** feature an unusual threonine tag that is longer than the predicted collinearity assembly lines. The structural diversity of burriogladiodins was derived from the relaxed substrate specificity of the fifth adenylation domain as well as chain termination conducted by water or threonine. The recombinase-mediating genome editing system is not only applicable in *B. gladioli,* but also possesses great potential for mining meaningful silent gene clusters from other *Burkholderia* species.

## 1. Introduction

The genus *Burkholderia* belongs to the beta subdivision of proteobacteria and occupies diverse ecological niches ranging from terrestrial and aquatic niches as free-living organisms to in association with eukaryotic hosts [1,2,3,4]. It exhibits great potential in the production of a variety of potent antibacterial, antitumor, herbicidal and insecticidal compounds [5,6]. Genome analysis showed a large number of natural product biosynthetic gene clusters in *Burkholderia*, presenting an abundant reservoir of nonribosomal peptides and polyketides, which are of particular interest due to their various biological properties [3,7]. Based on the genomic-guided discovery technologies associated with genome data, diverse compounds from *Burkholderia* have been discovered, such as bolagladins/glidochelins, gladiofungins, thailandepsins/burkholdacs [8,9,10,11,12]. However, many silent BGCs embedded in the *Burkholderia* genome still needs to be investigated.

During our ongoing genome mining efforts to discover new bioactive compounds from *Burkholderia* sensu lato, the model plant pathogen *Burkholderia gladioli* ATCC 10248, which potentially produces new structures based on bioinformatic analysis, attracted our interests [4,13,14,15,16]. Its genome size is estimated to be 8.9 Mbp with 68% of the G + C content, harboring two chromosomes (NZ_CP009323.1, NZ_CP009322.1) and three free plasmids (NZ_CP009321.1, NZ_CP009320.1, NZ_CP009319.1) [17]. The antiSMASH analysis predicted 19 biosynthetic gene clusters (BGCs) [18]. At present, only three types of natural products, polyketide gladiolins and nonribosomal peptide icosalides as well as sulfazecin, have been discovered in this model strain, while the others were likely dormant treasure trove and needed to be awakened through appropriate technical methods [19,20,21].

Promoter engineering has been proven as a useful strategy in the activation of silent gene clusters [22]. Through substitution of the native promoter of dedicated BGC with a constitutive or inducible promoter, transcriptional regulation of the BGC in the original producer could be bypassed. However, this methodology requires genome editing tools that should be workable in the target microorganisms. Red/ET recombineering technology mediated by λ phage Redα/Redβ or Rac prophage RecE/RecT recombinases is an efficient genetic engineering method and was used primarily in *Escherichia coli* for genome editing by using short homology arms (40–50 bp) [23].

Due to the limits of the Red/ET recombination system in the other microorganisms, our group recently established two other recombination techniques based on the homology to the recombinases for *Burkholderia* genus and *Pseudomonas* genus, respectively [13,24]. One is Redγ-Redαβ7029, which was discovered from *Schlegelella brevitalea* DSM 7029 (previously known as Burkholderiales strain) and is workable in several Burkholderiales strains [13,15,25]. The other is a lambda Red-like recombination system BAS from *Pseudomonas aeruginosa* phage Ab31 that was established to carry out genome editing in four *Pseudomonas* species [24]. These recombineering-mediated genome editing systems provide us a convenient alternative for gene manipulation of the target *B. gladioli* strain ATCC 10248.

**Figure 1:**
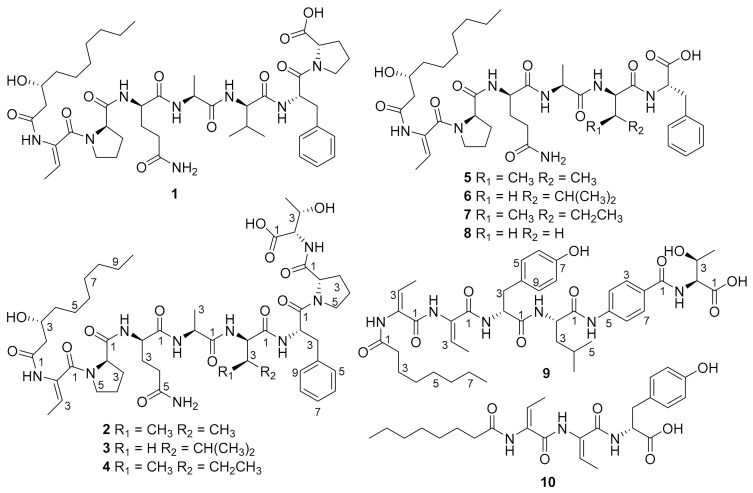
Structures of compounds **1**–**10**.

In this work, we first screened an applicable genome editing recombination system for *B. gladioli* ATCC 10248 and used it to activate two silent nonribosomal peptide synthetase (NRPS) BGCs by insertion of potent exogenous promoters. Ten new lipopeptides, burriogladiodins A–H (**1**–**8**) and haereogladiodins A–B (**9**–**10**) (Figure 1), were identified through HRESIMS, NMR, and Marfey’s analysis.

## 2. Results and Discussion

### 2.1. Bioinformatic Analysis and Manipulation of Silent BGCs in B. gladioli ATCC 10248

Bioinformatic analysis with the aid of the antiSMASH platform showed nineteen putative secondary metabolite BGCs in the genome of *B. gladioli* ATCC 10248, including six BGCs in chromosome 1 and thirteen BGCs in chromosome 2 (Table S1) [18]. Except for known gladiolin, icosalide and sulfazecin BGCs [19,20,21], the remaining five NRPSs, two polyketide synthases (PKSs), and one NRPS-PKS hybrid clusters exhibited difference to the known BGCs, indicating the potential of new secondary metabolites production in ATCC 10248. Among the NRPS BGCs, BGC 2 and BGC 5 on the chromosome 2 (Chr2C2 and Chr2C5), attracted our attention for containing a starter condensation (C_s_) domain putatively responsible for the biosynthesis of lipopeptides, which are remarkable classes of pharmaceutical molecules with distinctive antibacterial, antifungal, or surfactant activities [26,27].

According to bioinformatics prediction, Chr2C2 and Chr2C5 are conserved among some plant-pathogenic *Burkholderia* species producing unusual threonine-tagged lipopeptides, such as *B. glumae* and *B. plantarii* [28,29]. Based on the predicted substrate specificity of the assembly lines, Chr2C2 and Chr2C5 probably synthesize heptapeptide and pentapeptide with FA-Thr-Pro-Gln-Ala-X-Phe-Pro and FA-Thr-Thr-X-X-Pro backbones, respectively. LC–MS analysis of the crude extracts from the culture of ATCC 10248 did not show corresponding products under our laboratory conditions, indicating that Chr2C2 and Chr2C5 are silent.

### 2.2. Screening an Available Recombineering Genome Editing System for B. gladioli ATCC 10248

Due to the lack of feasible genetic tools for the manipulation of ATCC 10248, we set out to establish an efficient recombination system in the strain by introducing phage recombinases, which showed high efficiency in genome editing of other strains. Three recombination systems, Redγβα, Redγ-Redαβ7029, and Redγ-BAS, were employed to perform genome editing in ATCC 10248. Redγβα from *E. coli* and Redγ-Redαβ7029 from *S. brevitalea* DSM 7029 have been used in the genome editing of the *Burkholderia* species, while the recombinases BAS from *P. aeruginosa* which is closely related to *Burkholderia gladioli* ATCC 10248 [13,24,30].

**Figure 2:**
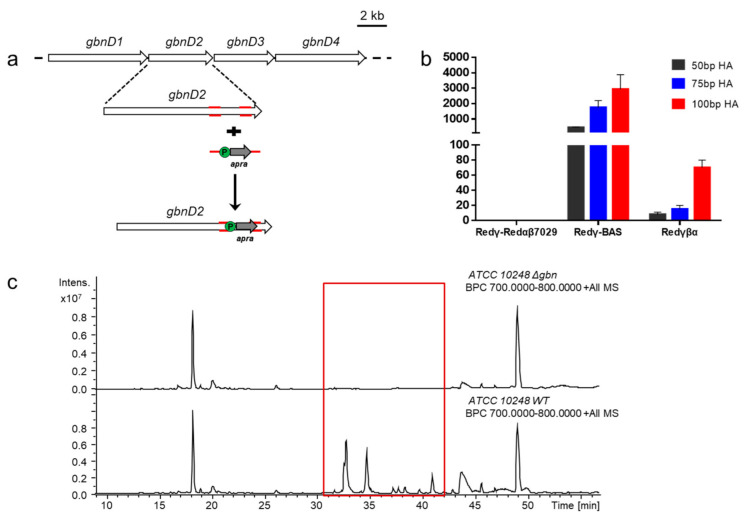
Assay of recombination systems in *B. gladioli* ATCC 10248. (**a**) Diagram of knockout of gladiolin gene cluster in strain ATCC 10248. (**b**) Recombination efficiency comparison of three recombinases Redγ-Redαβ7029, Redγ-BAS, and Redγβα. (**c**) Validation of gladiolin biosynthetic gene clusters (BGC) knockout through LC–MS analysis of the metabolic profiles of wild type and *Δgbn* mutant.

To investigate the applicability of the three recombination systems in ATCC 10248 genome modification, the three recombination systems were first electro-transformed into ATCC 10248, respectively. Then an apramycin resistance gene flanked with homology arms of different length (50 bp, 75 bp, 100 bp) was transformed in the three transformants, respectively, and used to replace the 1276 bp fragment (468577–469853) of the gladiolin gene cluster (*gbn*) on chromosome 2 (Figure 2a). The recombinants were verified by colony PCR and abolishment of gladiolins production (Figure 2c). The results showed that Redγ-BAS could efficiently mediate genome modification with all three selected lengths of the homology arms. Redγβα also functioned with the three lengths of homology arms, but with a low colony-forming unit (CFU) of ~8, ~15, and ~70, respectively. Unexpectedly, Redγ-Redαβ7029 was ineffective in ATCC 10248 even when 100 bp-homology arms were used (Figure 2b). Therefore, the Redγ-BAS recombination system was used for genome mining in ATCC 10248.

### 2.3. Activating Two Silent Biosynthetic Gene Clusters and Structure Elucidation of Lipopeptides

**Figure 3:**
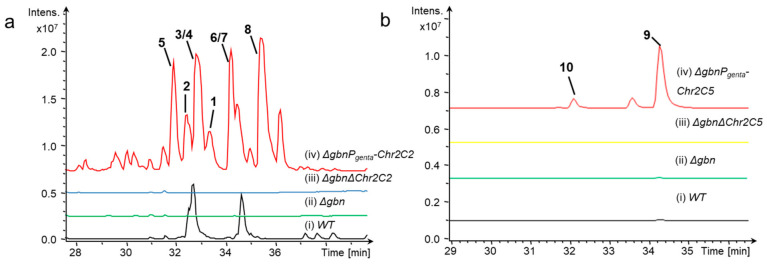
Mining of two silent biosynthetic gene clusters in *B. gladioli* ATCC 10248. (**a**) HPLC–MS analysis (BPC 700–1200) of crude extracts from wild type and mutants. (**b**) HPLC-MS analysis (BPC 474, 807) of crude extracts from wild type and mutants.

To activate the silent Chr2C2 and Chr2C5 gene clusters, the original promoters of the target BGCs were replaced by the constructive promoter P_genta_ associated with the gentamicin resistance gene. In order to avoid interference from the high yield of gladiolin, we constructed the gladiolin-deficient mutant by inserting an apramycin resistance gene in the gladiolin gene cluster (Figures S1 and S2). The inactivation mutants of the Chr2C2 and Chr2C5 gene clusters were constructed by gentamicin resistance gene replacing the core domains of the target genes. The mutants were fermented, and the compounds were extracted. Subsequent LC–MS analysis showed six new peaks in the Chr2C2 activation mutant (*m*/*z* 911 (**1**), 1012 (**2**), 1026 (**3** and **4**), 814 (**5**), 828 (**6** and **7**), and 786 (**8**) [M + H]^+^), and two new peaks in the Chr2C5 activation mutant (*m*/*z* 807 (**9**) and 474 (**10**) [M + H]^+^) compared with the wild type ATCC 10248 and inactivation mutants ATCC 10248Δ*gbn*Δ*Chr2C2* and ATCC 10248Δ*gbn*Δ*Chr2C5* (Figure 3).

**Figure 4:**
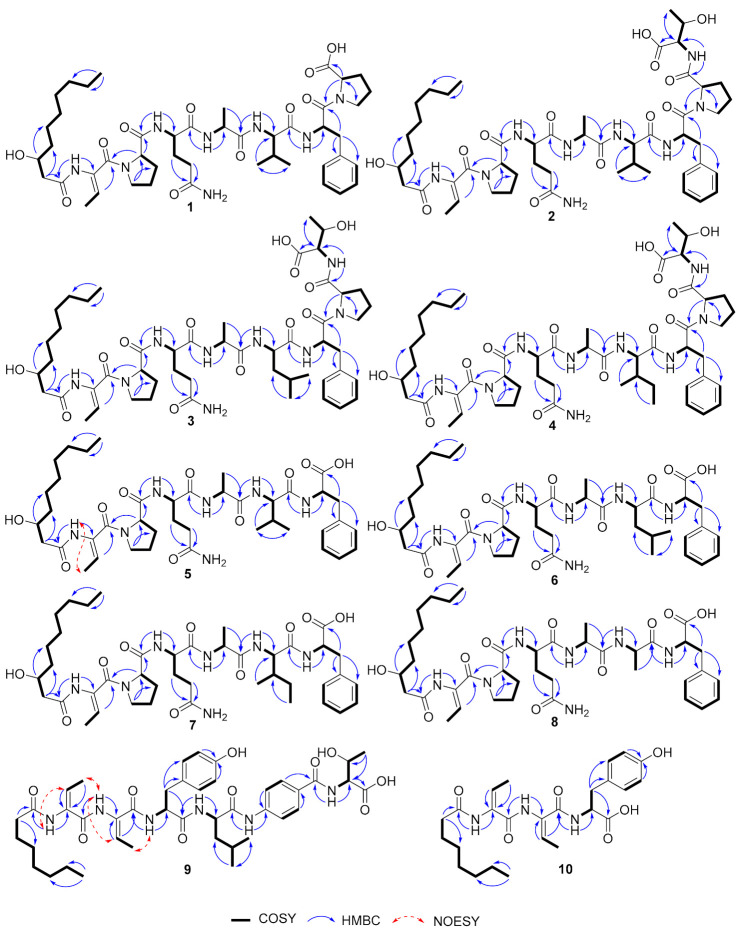
Key (^1^H–^1^H correlation spectroscopy) COSY, (Heteronuclear multiple bond coherence spectroscopy) HMBC, and (Nuclear overhauser effect spectroscopy) NOESY correlations of compounds **1**–**10**.

Burriogladiodin A (**1**) was obtained as a white solid with the molecular formula deduced to be C_46_H_70_N_8_O_11_ (HRESIMS, m/z 911.5220 [M + H]^+^, calcd 911.5237). According to the ^1^H and ^13^C NMR data of **1** (Table 1), compound **1** contains seven amino acid moieties: Dhb, two Pro, Gln, Ala, Val, and Phe, which was closely related to burriogladin A isolated from *B. gladioli* pv. *agaricicola* with the difference of the *p*-hydroxyphenyl glycine (*p*-Hpg), which is replaced by a Val in **1** [28], as suggested by the HMBC correlations from the Val H-2 to Val C-1/C-3/C-4/C-5, from Val H-4/H-5 to C-2/C-3, and supported by successive COSY correlations between Val H-2/H-3/H-4 or H-5. The detailed structure of **1** was further confirmed by the 2D NMR correlations (Figure 4), MS/MS fragmentations and Marfey’s analysis (Figures S3 and S4). The configuration of amino acid residues was determined to be D and L-Pro, D-Gln, L-Ala, D-Val, and L-Phe. The D-amino acids were predicted to be generated by the corresponding C_d_ domain in the assembly line with dual epimerization and condensation activity [31]. The absolute configuration at C-3 of *β*-OH-decanoate (*β*-OH-Dec) was also proposed to be *3R* because its C_s_ domain showed high homology to the C_s_ domain of burriogladins (99% identity) [28].

Burriogladiodin B (**2**) was also isolated as a white solid with the molecular formula C_50_H_77_N_9_O_13_ (HRESIMS, *m/z* 1012.5693 [M + H]^+^, calcd 1012.5714). Preliminary NMR analysis of **2** (Table 1) showed a close similarity to **1** except for several additional typical Thr signals. The Thr fragment connected to Pro2 via amide bond was suggested by obvious changes of the chemical shifts of the Pro2 part and evidenced by 2D NMR correlations from Thr NH to Thr C-2 and Pro2 C-1 (Figure 4). The configurations of Thr from **2** were confirmed to be L-type by Marfey’s analysis, while the other amino acids were identical to **1** (Table S3).

Burriogladiodins C (**3**) and D (**4**) could not be separated by reverse-phase C_18_ column and thus existed as a 2:1 (as calculated from the ^1^H NMR spectra integral) mixture of isomers in DMSO-*d_6_*. They have the same molecular formula, C_51_H_79_N_9_O_13_, deduced by the HRESIMS spectrum on the protonated ion peak at *m/z* 1026.5851 [M + H]^+^. The NMR data of **3**–**4** (Table 2) showed a high similarity to **2** except for one additional methylene signals (*δ*_C_ 41.4 in **3**, 25.4 in **4**). HMBC correlations from Leu or Ile H-1 to C-1/C-3/C-4 and H-5/H-6 to C-3/C-4 together with series COSY correlations between Leu or Ile NH/H-2/H-3/H-4/H-5 or H-6 clearly indicated a Leu in **3** and an Ile in **4** instead of the Val in **2**, respectively. Finally, the complete structures of **3** and **4** were elucidated unambiguously by 2D-NMR correlations (Figure 4) and also fulfilled with tandem MS/MS analysis and feeding experiment (Figure S5). The absolute configurations of Leu in **3** and Ile in **4** were both determined to be D configuration by Marfey’s analysis (Table S3).

HRESIMS of burriogladiodins E–H (**5–8**) gave three separate peaks with *m/z* [M + H]^+^ value of 814.4709 (**5**), 828.4853 (**6**, **7** as a mixture), and 786.4390 (**8**). The proposed molecular formulas of burriogladiodins E-H are C_41_H_63_N_7_O_10_, C_42_H_65_N_7_O_10_ and C_39_H_59_N_7_O_10_, respectively. Burriogladiodins E-H (**5**–**8**) were found to have similar NMR spectra (Table 3 and Table 4) to **1**–**4**. Compared to **1**, compounds **5**–**8** showed the common feature of the disappearance of the Pro moiety. Moreover, the main difference between **6** and **7** (Table 4), obtained as a mixture in the ratio of ca. 3:4, was originated from the substitution of Leu in **6** by Ile in **7**. A comparison of the 1D NMR data of **8** (Table 3) with **5** undoubtedly demonstrated that **8** had an Ala in place of the Val in **5**. The planar structures of **5**–**8** were further confirmed by the 2D NMR correlations (Figure 4) and MS/MS analysis as well as the feeding experiment (Figures S6 and S7). The configuration of the Dhb units in **1**–**8** was determined to be *Z* configuration by NOESY correlation, represented by compound **5** (Figure 4) and their biosynthesis consideration.

Haereogladiodins A (**9**) and B (**10**) were isolated as white solid and their molecular formulas were determined to be C_42_H_58_N_6_O_10_ (HRESIMS, *m/z* 807.4272 [M + H]^+^, calcd 807.4287) and C_25_H_35_N_3_O_6_ (HRESIMS, *m/z* 474.2600 [M + H]^+^, calcd 474.2599), respectively. The 1D NMR data of **9** and **10** (Table 5) were similar to haereoglumins A and B, but with the difference in the first amino acid [28], which showed a Tyr in **9** and **10**. Their structures were further confirmed by the 2D NMR correlations (Figure 4) and MS/MS fragmentations (Figure S8). The *E*-geometry of the double bonds in Dhb was determined by the NOESY correlations (Figure 4). According to Marfey’s analysis, the absolute configuration of the amino acids in **9** and **10** are D-Tyr, L-Leu, and L-Thr, respectively.

### 2.4. Biosynthesis of Burriogladiodins and Haereogladiodins

**Figure 5:**
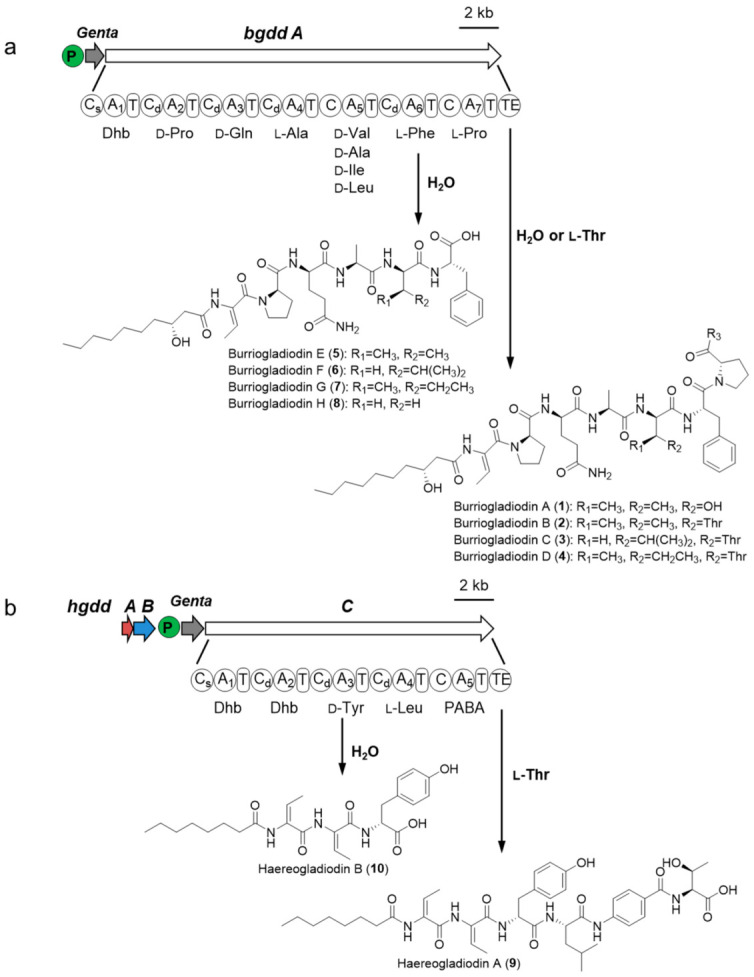
The proposed biosynthetic pathway of two silent biosynthetic gene clusters in *B. gladioli* ATCC 10248. (**a**) The NRPS module architecture of biosynthetic pathway of Chr2C2 (*bgdd*). *P*: promoter P_genta_; *Genta*: gentamicin resistance gene; *Bgdd A*: nonribosomal peptide synthetase (NRPS) (**b**) The NRPS module architecture of biosynthetic pathway of Chr2C5 (*hgdd*). *Hgdd A*: Glyoxalase; *Hgdd B*: para-aminobenzoic acid (PABA) synthase; *Hgdd C*: NRPS.

Accurate structural determination assisted with bioinformatic analysis allowed us to propose the biosynthetic mechanisms of burriogladiodins and haereogladiodins (Figure 5a,b). The elucidated structure of burriogladiodin A (**1**) is consistent with the chemical backbone predicted by in silico BGC analysis, while burriogladiodins B–D (**2**–**4**) with the additional C-terminal threonine tag are assumed to be introduced by the TE domain. The C-terminal threonine tag has been found in the biosynthesis of burrioplantin/burriogladins/burrioglumins, of which the BGCs showed high homology with the *bgdd* gene cluster [28,29]. The structural diversities of burriogladiodins B–D (**2**–**4**) is proposed to be generated by the substrate flexibility (Val/Leu/Ile) of the A_5_ domain. In addition, premature termination of the elongation in the assembling line led to the formation of the four truncated burriogladiodins E–H (**5**–**8**). Hereogladiodins are proposed to share similar biosynthetic mechanisms with burriogladiodins. Compared to **9**, hereogladiodin B (**10**) was an early hydrolysis product like **5**–**8** and our previously discovered holrhizins [14].

### 2.5. The Bioactivity Assays of Burriogladiodins and Haereogladiodins

Bioactivity test of compounds **1**–**10** showed no obvious activities against our selected four Gram-positive and Gram-negative bacteria (MIC > 100 μM) and as well as six tumor cell lines and normal cell line 293 T (IC_50_ > 20 μM, Table S4). Since lipopeptides often mediate important processes such as biofilm formation and swarming motility, and the lipopeptide with unusual threonine-tag are conserved in mushroom and plant pathogenic *Burkholderia* and environmental *Paraburkholderia*, which could promote bacterial infection in the host [15,28,29], we performed swarming and swimming assays with wild type and mutants to verify the activities in the bacterial cell motility (Figure S9). The swarming and swimming assays showed that the activation mutant swarmed and colonized in a bigger area compared to the wild-type strain. However, the activation mutant strain colonized in a smaller area compared to the gladiolin-deficient mutant. The gladiolins could probably inhibit the swarming ability of wild type, while burriogladiodins and haereogladiodins may promote the swarming ability of strain ATCC 10248.

In this work, we successfully established a recombination system for genome mining in *B. gladioli* ATCC 10248 and activated two silent NRPS BGCs by inserting a potent exogenous promoter. Two new classes of lipopeptides, burriogladiodins (**1**–**8**) and haereogladiodins (**9**–**10**) were isolated and elucidated, which enriched a new member of linear lipopeptides. According to the swarming and swimming assays, these compounds probably play a role in bacteria invasion of plant hosts.

## 3. Materials and Methods

### 3.1. General Experimental Procedures

Optical rotations were obtained on a JASCO *P*-1020 digital polarimeter (JASCO Corporation, Tokyo, Japan). UV spectra were recorded on a Thermo Scientific Dionex Ultimate 3000 DAD detector, and IR spectra were taken on a Nicolet NEXUS 470 spectrophotometer as KBr disks (Thermo Fisher Scientific, Waltham, MA, USA). ^1^H and ^13^C NMR, DEPT, and 2D NMR spectra were recorded on an Agilent 500 MHz DD2 (Agilent Technologies Inc., Santa Clara, CA, USA) using TMS as an internal standard. HRESIMS spectra were measured on a Bruker Impact HD microTOF Q III mass spectrometer (Bruker, Rheinstetten, Germany) using the standard ESI source. UHPLC-MS was operated using a Thermo Scientific Dionex Ultimate 3000 system coupled with the Bruker amazon SL Ion Trap mass spectrometry, controlled by Hystar v3.2 and Chromeleon Xpress software. A Thermo Scientific™ Acclaim™ C_18_ column (2.1 × 100 mm, 2.2 μm) was used. The mobile phase consisted of H_2_O and acetonitrile (ACN), both containing 0.1% formic acid. Semipreparative HPLC (Agilent Technologies Inc., Santa Clara, CA, USA) was performed using an ODS column (Bruker ZORBAX SB-C_18_, 9.4 × 250 mm, 5 μm, 3 mL min^−1^). Vacuum-liquid chromatography (VLC) was carried out over silica gel H (Qingdao Marine Chemical Factory, Qingdao, China).

### 3.2. Bacterial Strains, Plasmids and Reagents

The strains, mutants and plasmids used in this study are lists in Table S5. *B. gladioli* ATCC 10248 was ordered from the China General Microbiological Culture Collection Center (CGMCC). *B. gladioli* ATCC 10248 and mutant strains were cultured on CYMG medium (casein peptone 8 g L^−1^, Yeast extract 4 g L^−1^, MgCl_2_·6H_2_O 8.06 g L^−1^, Glycerol 5 mL L^−1^) broth or agar plates at 30 °C. The mutant strains were grown in the presence of 30 μg mL^−1^ kanamycin [km], 120 μg mL^−1^ gentamicin [genta] and 250 μg mL^−1^ apramycin [apra].

### 3.3. The Construction of Optimized Recombination System in B. gladioli ATCC 10248

The procedure of the chromosome modification was mediated by linear and circle homology recombination (LCHR). Three recombination systems including Redγβα, Redγ-Redαβ7029, and Redγ-BAS were used in this study [13,23,25]. The effect of the length of the homologous arm on recombination efficiency was explored by using an apramycin resistance gene flanked with varying length homology arms (50 bp, 75 bp, 100 bp) to replace the fragment (468577–469901) of the gladiolin biosynthetic gene cluster in ATCC 10248/pBBR1-Rha-Redγ-Redαβ7029-km, ATCC 10248/pBBR1-Rha-Redγ-BAS-km, and ATCC 10248/pBBR1-Rha-Redγβα-km.

### 3.4. Knockout and Promoter Insertion of the Silent Gene Clusters on the Chromosome of B. gladioli ATCC 10248

The target genes were knocked out by the gentamicin resistance gene using the Redγ-BAS system. The target BGC activation mutant was constructed by the insertion of a constructive promoter (P_genta_), replacing the original promoter in front of the main biosynthetic gene of BGCs. The antibiotic resistance gene and constructive promoter flanked with homology arms (50 bp) were generated by polymerase chain reaction (PCR) amplification using 2 × PrimerSTAR Max polymerases (Takara Biomedical Technology (Beijing) Co., Ltd., Beijing, China), and the templates for genta^R^, P_genta_ and apra^R^ are derived from plasmids R6K-lox71-genta-lox66-FleQ and RK2-apra-cm, respectively. For the recombineering, purified PCR products of the resistance gene were transformed into *B. gladioli* ATCC 10248/pBBR1-Rha-Redγ-BAS-km, respectively. Recombinants were selected on CYMG plates containing gentamicin (120 μg mL^−1^) or apramycin (250 μg mL^−1^), respectively. Correct recombinants were verified by colony PCR. A list of recombinants generated in this study is provided in Table S5. Primers used for gene cluster modification are listed in Table S6.

### 3.5. Extraction and Isolation

The recombinant *B. gladioli* ATCC 10248*ΔgbnP_genta_-bgdd* and *B. gladioli* ATCC 10248Δ*gbnP_genta_-hgdd* were fermented in 20 L of CYMG medium supplemented with 30 μg mL^−1^ km at 30 °C, 200 rpm for 3 days, and then added 2% XAD 16 (*v*/*v*) incubated for another 1 day. The resin was collected by sieving, washed with double distilled H_2_O (ddH_2_O), and then extracted with methanol (5 L). The extracts were concentrated under reduced pressure. The final crude extracts were subjected to vacuum liquid chromatography (VLC) on a silica gel column using step gradient elution with CH_2_Cl_2_ and MeOH (1:0 to 0:1) to separate into several fractions. The subfractions containing target compounds further purified by semipreparative reverse-phase HPLC (Agilent ZORBAX SB-C_18_, 9.4 × 250 mm, 5 μm, 3 mL min^−1^) using ACN and H_2_O contained 0.1% TFA as mobile phase with the following conditions: 0−5 min, 42% ACN; 5−30 min, 42−52% ACN; 30.1 min, 95% ACN; 30.1−35 min, 95% ACN to yield **1** (11 mg, t_R_ = 26 min, 0.55 mg/L); 0−5 min, 40% ACN; 5−28 min, 40−48% ACN; 28.1 min, 95% ACN; 28.1−33 min, 95% ACN to yield **2** (6 mg, t_R_ = 24 min, 0.3 mg/L); constant gradient 42% ACN to yield **3**/**4** (15 mg, t_R_ = 23 min, 0.75 mg/L), and **6**/**7** (17 mg, t_R_ = 25 min, 0.85 mg/L); constant gradient 46% ACN to yield **8** (6 mg, t_R_ = 23 min); constant gradient 42% ACN to yield **9** (8 mg, t_R_ = 30 min, 0.4 mg/L) and **10** (5 mg, t_R_ = 23 min, 0.25 mg/L).

*Burriogladiodin A* (**1**): white solid, [*α*] ^20^_D_ -8 (*c* 0.25, MeOH), *λ*_max_ 220 nm; IR (KBr) *v*_max_ 3302, 2929, 1653, 1541, 1445, 1201, 1027, 701 cm^−1^; ^1^H and ^13^C NMR, Table 1; HRESI/MS: *m/z* 911.5220 [M + H]^+^ (calculated for C_46_H_70_N_8_O_11_, 911.5237).

*Burriogladiodin B* (**2**): white solid, [*α*] ^20^_D_ -27 (*c* 0.16, MeOH), *λ*_max_ 220 nm; IR (KBr) *v*_max_ 3290, 2930, 1653, 1542, 1439, 1202, 1026, 700 cm^−1^; ^1^H and ^13^C NMR, Table 1; HRESI/MS: *m/z* 1012.5693 [M + H]^+^ (calculated for C_50_H_77_N_9_O_13_, 1012.5714).

*Burriogladiodins C*–*D* (**3**–**4**): white solid, *λ*_max_ 220 nm; IR (KBr) *v*_max_ 3294, 2929, 1686, 1543, 1439, 1202, 701 cm^−1^; ^1^H and ^13^C NMR, Table 2; HRESI/MS: *m/z* 1026.5851 [M + H]^+^ (calculated for C_51_H_79_N_9_O_13_, 1026.5870).

*Burriogladiodin E* (**5**): white solid, [*α*] ^20^_D_ -15 (*c* 0.15, MeOH), *λ*_max_ 220 nm; IR (KBr) *v*_max_ 3294, 2929, 1637, 1541, 1437, 1027, 699 cm^−1^; ^1^H and ^13^C NMR, Table 3; HRESI/MS: *m/z* 814.4695 [M + H]^+^ (calculated for C_41_H_63_N_7_O_10_, 814.4709).

*Burriogladiodins F*–*G* (**6**–**7**): white solid, *λ*_max_ 220 nm; IR (KBr) *v*_max_ 3298, 2930, 1653, 1542, 1439, 1203, 1027, 701 cm^−1^; ^1^H and ^13^C NMR, Table 4; HRESI/MS: *m/z* 828.4853 [M + H]^+^ (calculated for C_42_H_66_N_7_O_10_, 828.4866).

*Burriogladiodin H* (**8**): white solid, [*α*] ^20^_D_ -3 (*c* 0.10, MeOH), *λ*_max_ 220 nm; IR (KBr) *v*_max_ 3312, 2928, 1654, 1541, 1206, 703 cm^−1^; ^1^H and ^13^C NMR, Table 3; HRESI/MS: *m/z* 786.4390 [M + H]^+^ (calculated for C_39_H_59_N_7_O_10_, 786.4396).

*Haereogladiodin A* (**9**): white solid, [*α*] ^20^_D_ -9 (*c* 0.30, MeOH), *λ*_max_ 220 nm; IR (KBr) *v*_max_ 3303, 2931, 1679, 1518, 1207, 1027, 722 cm^−1^; ^1^H and ^13^C NMR, Table 5; HRESI/MS: *m/z* 807.4272 [M + H]^+^ (calculated for C_42_H_58_N_6_O_10_, 807.4287).

*Haereogladiodin B* (**10**): white solid, [*α*] ^20^_D_ +15 (*c* 0.10, MeOH), *λ*_max_ 220 nm; IR (KBr) *v*_max_ 3261, 2922, 1647, 1517, 1245, 1025, 829, 653 cm^−1^; ^1^H and ^13^C NMR, Table 5; HRESI/MS: *m/z* 474.2600 [M + H]^+^ (calculated for C_25_H_35_N_3_O_6_, 474.2599).

### 3.6. Feeding of Labeled Amino Acids

The detailed procedure was reported by our previous study [14]. The final crude extracts were dissolved with 150 μL MeOH. The crude extracts were analyzed by HPLC-MS. The HPLC-MS condition: 0–5 min, 30% ACN; 5–50 min, 30–75% ACN; 50.1 min, 95% ACN; 50.1–55 min, 95% ACN; and 55.1–60 min, 30% ACN. ACN and H_2_O contained 0.1% formic acid. The labeled precursors L-Val (D8, 98%), L-Ala (3, 3, 3–D3, 99%), L-Ile (^15^ N, 98%), and L-Leu (1,2–^13^ C2, 99%) were purchased from Cambridge Isotope Laboratories.

### 3.7. Antibacterial and Cytotoxic Activities Assay

The antibacterial activities of compounds were evaluated using Kirby–Bauer disk diffusion method. The tested bacteria included Gram-negative bacteria *Escherichia coli* ATCC 35218 and *Pseudomonas aeruginosa* ATCC 27853, and Gram-positive bacteria *Staphylococcus aureus* ATCC 29213 and *Bacillus subtilis* ATCC 6633. The tested microorganisms were obtained from China General Microbiological Culture Collection Center (CGMCC). The tested cells contained human hematological disease cells K562, human breast adenocarcinoma cells MCF7, human hepatoma cell HepG-2, human lung adenocarcinoma cell A549, human negroid cervix epithelioid carcinoma Hela, human colon cancer cell HCT-116, and human lung normal cell 2B. The detailed procedure was performed according to methods previously described [14].

### 3.8. Swarming and Swimming Assay

The hot CYMG-0.5% agar and hot CYMG-0.25% agar (15 mL) was poured into Petri dishes for swarming assay and swimming assay, respectively [28,32]. The plates were dried. The overnight bacterial culture in CYMG was diluted to get the OD_600_ to 0.1. The suspension (3 μL) was carefully dropped at the center of the agar plate. The plates were incubated at 30 °C for 36 h.

## Figures and Tables

**Table 1:** The ^1^H (500 MHz) and ^13^C NMR (125 MHz) data of **1**–**2** in DMSO-*d*_6._

	No	1		2	
		*δ*_C_	*δ*_H_ (*J* in Hz)	*δ*_C_	*δ*_H_ (*J* in Hz)
*β*-OH-Dec	1	172.1, C		172.1, C	
	2	42.6, CH_2_	2.37, d (6.4)	42.6, CH_2_	2.36, d (6.5)
	3	67.0, CH	3.88, m	67.0, CH	3.87, m
	4	37.3, CH_2_	1.30, m	37.3, CH_2_	1.30, m
	5	25.0, CH_2_	1.23, m ^a^	25.0, CH_2_	1.23, m ^a^
	6	29.1, CH_2_	1.23, m ^a^	29.1, CH_2_	1.23, m ^a^
	7	28.7, CH_2_	1.23, m ^a^	28.8, CH_2_	1.23, m ^a^
	8	31.3, CH_2_	1.23, m ^a^	31.3, CH_2_	1.23, m ^a^
	9	22.1, CH_2_	1.23, m ^a^	22.1, CH_2_	1.23, m ^a^
	10	14.0, CH_3_	0.86, t (6.7)	14.0, CH_3_	0.86, t (7.1)
Dhb	1	166.9, C			
	2	132.1, C		166.9, C	
	3	120.7, CH	5.64, q (6.8)	132.1, C	5.64, q (6.8)
	4	11.9, CH_3_	1.67, d (6.8)	120.8, CH	1.66, d (6.8)
	2-NH	166.9, C	9.71, s	12.0, CH_3_	9.71, s
Pro	1	171.2, C		171.3, C	
	2	61.0, CH	4.20, t (7.6)	61.0, CH	4.20, t (7.2)
	3a	29.3, CH_2_	2.16, m	29.3, CH_2_	2.16, m
	3b		1.75, m ^a^		1.74, m ^a^
	4a	24.9, CH_2_	1.84, m ^a^	24.9, CH_2_	1.84, m ^a^
	4b		1.75, m ^a^		1.74, m ^a^
	5a	48.7, CH_2_	3.57, m	48.7, CH_2_	3.57, m
	5b	171.2, C	3.40, m	171.3, C	3.40, m
Gln	1	171.0, C		171.0, C	
	2	52.6, CH	4.07, m ^a^	52.6, CH	4.07, m ^a^
	3a	27.1, CH_2_	2.04, m ^a^	27.1, CH_2_	2.04, m ^a^
	3b		1.84, m ^a^		1.84, m ^a^
	4	32.2, CH_2_	2.04, m ^a^	32.2, CH_2_	2.04, m ^a^
	5	174.2, C		174.2, C	
	2-NH	171.0, C	7.77, d (8.7)	171.0, C	7.77, d (8.5)
Ala	1	172.0, C		172.0, C	
	2	49.4, CH	4.07, m ^a^	49.5, CH	4.07, m ^a^
	3	17.7, CH_3_	1.19, d (7.3)	17.8, CH_3_	1.19, d (7.3)
	2-NH		7.52, d (6.4)	172.0, C	7.52, d (6.3)
Val	1	170.1, C		170.1, C	
	2	56.8, CH	4.14, dd (6.9, 9.4)	56.7, CH	4.14, m ^a^
	3	30.8, CH	1.66, m	30.8, CH	1.65, m
	5	18.9, CH_3_	0.46, d (6.9)	18.9, CH_3_	0.44, d (6.2)
	6	17.4, CH_3_	0.44, d (6.9)	17.4, CH_3_	0.43, d (6.2)
	2-NH	170.1, C	7.21, d (7.9)	170.1, C	7.18, d (7.8)
Phe	1	169.6, C		169.8, C	
	2	51.8, CH	4.72, m	52.0, CH	4.71, m
	3a	36.8, CH_2_	2.97, dd (4.2, 14.0)	36.9, CH_2_	2.97, dd (3.4, 14.0)
	3b		2.72, dd (10.3, 14.0)		2.72, dd (10.7, 14.0)
	4	137.6, C		137.8, C	
	5/9	129.3, CH	7.27, d (7.2)	129.3, CH	7.27, d (7.3)
	6/8	128.0, CH	7.21, t (7.2)	128.0, CH	7.21, t (7.3)
	7	126.2, CH	7.14, t (7.2)	126.2, CH	7.13, t (7.3)
	2-NH	169.6, C	8.45, d (8.5)		8.44, d (8.5)
Pro	1	173.2, C		171.8, C	
	2	58.6, CH	4.27, dd (4.1, 8.8)	59.3, CH	4.54, dd (3.0, 8.3)
	3a	28.6, CH_2_	2.12, m	28.8, CH_2_	2.04, m ^a^
	3b		1.88, m ^a^		1.90, m ^a^
	4	24.5, CH_2_	1.88, m ^a^	24.5, CH_2_	1.90, m ^a^
	5	46.4, CH_2_	3.64, m	46.8, CH_2_	3.61, m
Thr	1			172.1, C	
	2			57.5, CH	4.21, m ^a^
	3			66.4, CH	4.14, m ^a^
	4			20.4, CH_3_	1.08, d (6.4)
	2-NH			172.1, C	7.76, d (8.6)

^a^ overlapped.

**Table 2:** The ^1^H (500 MHz) and ^13^C NMR (125 MHz) data of **3**–**4** in DMSO-*d*_6._

	No	3		4	
		*δ*_C_	*δ*_H_ (*J* in Hz)	*δ*_C_	*δ*_H_ (*J* in Hz)
*β*-OH-Dec	1	172.3, C		172.3, C	
	2a	42.6, CH_2_	2.37, dd (9.6, 14.4)	42.7, CH_2_	2.37, dd (9.6, 14.4)
	2b		2.24, dd (2.3, 14.4)		2.24, dd (2.3, 14.4)
	3	67.0, CH	3.90, m	67.0, CH	3.90, m
	4	37.4, CH_2_	1.32, m	37.3, CH_2_	1.32, m
	5	25.0, CH_2_	1.24, m ^a^	25.0, CH_2_	1.24, m ^a^
	6	29.2, CH_2_	1.24, m ^a^	29.1, CH_2_	1.24, m ^a^
	7	28.8, CH_2_	1.24, m ^a^	28.9, CH_2_	1.24, m ^a^
	8	31.3, CH_2_	1.24, m ^a^	31.4, CH_2_	1.24, m ^a^
	9	22.2, CH_2_	1.24, m ^a^	22.2, CH_2_	1.24, m ^a^
	10	14.0, CH_3_	0.86, t (6.3)	14.0, CH_3_	0.85, t (6.3)
Dhb	1	167.1, C		166.9, C	
	2	132.1, C		132.1, C	
	3	121.3, CH	5.67, q (6.8)	120.6, CH	5.62, q (6.8)
	4	12.0, CH_3_	1.68, d (6.8)	12.0, CH_3_	1.67, d (6.8)
	2-NH		9.74, s		9.69, s
Pro	1	171.2, C		171.3, C	
	2	61.2, CH	4.20, m ^a^	61.0, CH	4.20, m ^a^
	3a	29.3, CH_2_	2.17, m	29.3, CH_2_	2.17, m
	3b		1.76, m ^a^		1.76, m ^a^
	4a	25.0, CH_2_	1.83, m ^a^	24.9, CH_2_	1.83, m ^a^
	4b		1.76, m ^a^		1.76, m ^a^
	5a	48.6, CH_2_	3.68, m	48.7, CH_2_	3.68, m
	5b		3.38, m		3.38, m
Gln	1	170.9, C		171.1, C	
	2	52.6, CH	4.06, m ^a^	52.7, CH	4.06, m ^a^
	3a	27.2, CH_2_	2.03, m ^a^	27.1, CH_2_	2.03, m ^a^
	3b		1.83, m ^a^		1.83, m ^a^
	4	32.3, CH_2_	2.03, m ^a^	32.2, CH_2_	2.03, m ^a^
	5	174.3, C		174.2, C	
	2-NH		7.71, d (8.9)		7.76, d (8.9)
Ala	1	172.1, C		172.0, C	
	2	49.6, CH	4.00, m ^a^	49.4, CH	4.00, m ^a^
	3	17.6, CH_3_	1.19, d (7.2)	17.8, CH_3_	1.19, d (7.2)
	2-NH		7.52, d (6.5)		7.53, d (6.5)
Leu/Ile	1	171.9, C		170.4, C	
	2	50.1, CH	4.30, dd (4.5, 9.5)	55.0, CH	4.27, dd (6.1, 9.5)
	3a	41.4, CH_2_	1.01, m	37.3, CH	1.53, m
	3b		0.92, m		
	4a	23.7, CH	1.24, m ^a^	25.4, CH_2_	0.92, m ^a^
	4b				0.73, m ^a^
	5	23.0, CH_3_	0.69, d (6.8)	11.5, CH_3_	0.66, t (7.5)
	6	21.5, CH_3_	0.68, d (6.8)	13.9, CH_3_	0.39, d (6.7)
	2-NH		7.20, d ^a^		7.26, d ^a^
Phe	1	169.8, C		169.9, C	
	2	51.8, CH	4.67, m	52.1, CH	4.67, m
	3a	37.0, CH_2_	2.96, m	36.9, CH_2_	2.96, m
	3b		2.73, m		2.73, m
	4	137.8, C		137.8, C	
	5/9	129.5, CH	7.26, d (7.3)	129.3, CH	7.26, d (7.2)
	6/8	127.9, CH	7.20, t (7.3)	128.0, CH	7.20, t (7.2)
	7	126.1, CH	7.13, t (7.3)	126.2, CH	7.14, t (7.2)
	2-NH		8.42, d (8.5)		8.42, d (8.5)
Pro	1	171.8, C		171.9, C	
	2	59.3, CH	4.54, dd (2.1, 7.8)	59.3, CH	4.54, dd (2.1, 7.8)
	3a	28.8, CH_2_	2.03, m ^a^	28.9, CH_2_	2.03, m ^a^
	3b		1.92, m ^a^		1.92, m ^a^
	4a		2.03, m ^a^	24.5, CH_2_	2.03, m ^a^
	4b	24.5, CH_2_	1.92, m ^a^		1.92, m ^a^
	5	46.8, CH_2_	3.60, m	46.8, CH_2_	3.60, m
Thr	1	172.1, C		172.1, C	
	2	57.5, CH	4.20, m ^a^	57.5, CH	4.20, m ^a^
	3	66.5, CH	4.14, m	66.5, CH	4.14, m
	4	20.4, CH_3_	1.08, d (6.3)	20.4, CH_3_	1.08, d (6.3)
	2-NH		7.76, d (8.6)		7.77, d (8.6)

^a^ overlapped.

**Table 3:** The ^1^H (500 MHz) and ^13^C NMR (125 MHz) data of **5** and **8** in DMSO-*d*_6._

	No	5		8	
		*δ*_C_	*δ*_H_ (*J* in Hz)	*δ*_C_	*δ*_H_ (*J* in Hz)
*β*-OH-Dec	1	172.1, C		171.7, C	
	2a	42.7, CH_2_	2.42, dd (4.1, 14.6)	42.7, CH_2_	2.36, dd (8.7, 13.1)
	2b		2.37, dd (8.8, 14.6)		
	3	67.0, CH	3.90, m	67.1, CH	3.89, m
	4	37.3, CH_2_	1.32, m	37.2, CH_2_	1.34, m
	5	24.9, CH_2_	1.22, m ^a^	24.9, CH_2_	1.23, m ^a^
	6	29.0, CH_2_	1.22, m ^a^	29.0, CH_2_	1.23, m ^a^
	7	28.7, CH_2_	1.22, m ^a^	28.7, CH_2_	1.23, m ^a^
	8	31.3, CH_2_	1.22, m ^a^	31.3, CH_2_	1.23, m ^a^
	9	22.1, CH_2_	1.22, m ^a^	22.1, CH_2_	1.23, m ^a^
	10	13.9, CH_3_	0.85, t (6.7)	14.0, CH_3_	0.85, t (7.1)
Dhb	1	166.9, C		166.9, C	
	2	132.0, C		131.9, C	
	3	120.9, CH	5.64, q (6.9)	120.5, CH	5.61, q (7.1)
	4	12.0, CH_3_	1.67, d (6.9)	12.0, CH_3_	1.66, d (7.1)
	2-NH		9.69, s		9.71, s
Pro	1	171.3, C		171.3, C	
	2	60.9, CH	4.21, t (7.5)	60.8, CH	4.20, m ^a^
	3a	29.2, CH_2_	2.16, m	29.3, CH_2_	2.16, m
	3b		1.74, m ^a^		1.75, m ^a^
	4a	24.9, CH_2_	1.83, m ^a^	24.8, CH_2_	1.84, m ^a^
	4b		1.74, m ^a^		1.75, m ^a^
	5a	48.7, CH_2_	3.67, m	48.8, CH_2_	3.63, m
	5b		3.41, m		3.42, m
Gln	1	171.0, C		171.2, C	
	2	52.6, CH	4.09, m ^a^	52.7, CH	4.08, m
	3a	27.1, CH_2_	2.06, m ^a^	26.9, CH_2_	2.07, m ^a^
	3b		1.84, m ^a^		1.84, m ^a^
	4	32.2, CH_2_	2.06, m ^a^	32.0, CH_2_	2.07, m ^a^
	5	174.1, C		174.0, C	
	2-NH		7.77, d (8.8)		7.76, d (8.5)
Ala	1	172.1, C		171.7, C	
	2	49.5, CH	4.09, m ^a^	49.3, CH	4.03, m
	3	17.7, CH_3_	1.21, d (7.4)	17.4, CH_3_	1.18, d (7.2)
	2-NH		7.55, d (6.4)		7.57, d (6.3)
Val/Ala	1	170.3, C		171.6, C	
	2	56.9, CH	4.14, dd (6.9, 9.3)	47.5, CH	4.24, m
	3	30.6, CH	1.72, m	18.3, CH_3_	0.93, d (7.1)
	4	18.9, CH_3_	0.56, d (6.7)		
	5	17.3, CH_3_	0.51, d (6.7)		
	2-NH	170.3, C	7.25, d (9.2)		7.52, d (8.0)
Phe	1	172.8, C		172.8, C	
	2	53.4, CH	4.40, m	53.2, CH	4.42, m
	3a	36.8, CH_2_	3.06, dd (4.5, 13.8)	37.0, CH_2_	3.06, dd (4.9, 13.8)
	3b		2.83, dd (10.4, 13.8)		2.83, dd (9.6, 13.8)
	4	137.7, C	7.22, m ^a^	137.4, C	
	5/9	129.1, CH	7.22, m ^a^	129.2, CH	7.24, m ^a^
	6/8	128.0, CH	7.15, t (6.7)	128.1, CH	7.18, m ^a^
	7	126.3, CH	8.22, d (8.3)	126.3, CH	7.18, m ^a^
	2-NH				8.09, d (8.3)

^a^ overlapped.

**Table 4:** The ^1^H (500 MHz) and ^13^C NMR (125 MHz) data of **6**–**7** in DMSO-*d*_6._

	No	6		7	
		*δ*_C_	*δ*_H_ (*J* in Hz)	*δ*_C_	*δ*_H_ (*J* in Hz)
*β*-OH-Dec	1	172.0, C		171.9, C	
	2a	42.6, CH_2_	2.38, dd (9.7, 14.5)	42.7, CH_2_	2.39, dd (9.7, 14.5)
	2b		2.33, dd (3.4, 14.5)		2.38, dd (4.8, 14.5)
	3	67.1, CH	3.90, m	67.0, CH	3.90, m
	4	37.3, CH_2_	1.33, m	37.3, CH_2_	1.33, m
	5	24.9, CH_2_	1.23, m ^a^	24.9, CH_2_	1.23, m ^a^
	6	29.1, CH_2_	1.23, m ^a^	29.1, CH_2_	1.23, m ^a^
	7	28.7, CH_2_	1.23, m ^a^	28.7, CH_2_	1.23, m ^a^
	8	31.3, CH_2_	1.23, m ^a^	31.3, CH_2_	1.23, m ^a^
	9	22.1, CH_2_	1.23, m ^a^	22.1, CH_2_	1.23, m ^a^
	10	14.0, CH_3_	0.85, t (6.7)	14.0, CH_3_	0.85, t (6.7)
Dhb	1	167.1, C		166.9, C	
	2	131.9, C		131.9, C	
	3	121.2, CH	5.65, q (6.9)	120.7, CH	5.62, q (7.0)
	4	12.0, CH_3_	1.68, d (6.9)	12.0, CH_3_	1.66, d (7.0)
	2-NH		9.74, s		9.68, s
Pro	1	171.3, C		171.3, C	
	2	61.1, CH	4.20, t (7.6)	60.9, CH	4.20, t (7.6)
	3a	29.3, CH_2_	2.17, m	29.3, CH_2_	2.17, m
	3b		1.75, m ^a^		1.75, m ^a^
	4a	24.9, CH_2_	1.83, m ^a^	24.8, CH_2_	1.83, m ^a^
	4b		1.75, m ^a^		1.75, m ^a^
	5a	48.6, CH_2_	3.68, m	48.7, CH_2_	3.68, m
	5b		3.42, m ^a^	171.3, C	3.42, m ^a^
Gln	1	171.0, C		171.1, C	
	2	52.6, CH	4.09, m ^a^	52.7, CH	4.09, m ^a^
	3a	27.1, CH_2_	2.06, m ^a^	27.1, CH_2_	2.06, m ^a^
	3b		1.83, m ^a^		1.83, m ^a^
	4	32.3, CH_2_	2.06, m ^a^	32.2, CH_2_	2.06, m ^a^
	5	174.2, C		174.1, C	
	2-NH		7.71, d (8.8)		7.77, d (8.7)
Ala	1	172.2, C		172.2, C	
	2	49.6, CH	4.02, m	49.4, CH	4.09, m ^a^
	3	17.5, CH_3_	1.20, d (7.2)	17.7, CH_3_	1.20, d (7.2)
	2-NH		7.55, d (5.9)		7.54, d (5.8)
Leu/Ile	1	171.5, C		170.6, C	
	2	50.1, CH	4.30, dd (4.8, 9.5)	55.1, CH	4.27, dd (6.1, 9.4)
	3	41.2, CH_2_	1.09, m	37.0, CH	1.52, m
	4a	23.7, CH	1.32, m ^a^	25.5, CH_2_	1.02, m ^a^
	4b				0.84, m ^a^
	5	23.0, CH_3_	0.71, d (6.5)	11.4, CH_3_	0.70, t (7.7)
	6	21.5, CH_3_	0.71, d (6.5)	13.9, CH_3_	0.46, d (6.8)
	2-NH		7.33, d (8.8)		7.26, d (9.4)
Phe	1	172.9, C		172.9, C	
	2	53.3, CH	4.38, m	53.4, CH	4.42, m
	3a	37.0, CH_2_	3.06, dd (3.8, 13.5)	36.8, CH_2_	3.06, dd (4.0, 13.8)
	3b		2.81, dd (4.4, 13.5)		2.83, dd (4.8, 13.8)
	4	137.6, C		137.7, C	
	5/9	129.2, CH	7.21, m ^a^	129.1, CH	7.21, m ^a^
	6/8	128.0, CH	7.21, m ^a^	128.1, CH	7.21, m ^a^
	7	126.3, CH	7.16, t (6.8)	126.3, CH	7.16, t (6.8)
	2-NH		8.15, d (8.4)		8.18, d (8.4)

^a^ overlapped.

**Table 5:** The ^1^H (500 MHz) and ^13^C NMR (125 MHz) data of **9**–**10** in DMSO-*d*_6._

	No	9		10	
		*δ*_C_	*δ*_H_ (*J* in Hz)	*δ*_C_	*δ*_H_ (*J* in Hz)
Octanoate acid	1	172.1, C		172.1, C	
	2	34.8, CH_2_	2.23, m	35.6, CH_2_	2.17, t (5.4)
	3	24.7, CH_2_	1.49, m ^a^	25.3, CH_2_	1.49, m
	4	28.6, CH_2_	1.23, m ^a^	28.9, CH_2_	1.22, m ^a^
	5	28.7, CH_2_	1.23, m ^a^	29.1, CH_2_	1.22, m ^a^
	6	31.2, CH_2_	1.19, m ^a^	31.6, CH_2_	1.22, m ^a^
	7	22.1, CH_2_	1.23, m ^a^	22.5, CH_2_	1.22, m ^a^
	8	13.9, CH_3_	0.79, t (6.9)	14.4, CH_3_	0.83, t (6.0)
Dhb	1	164.4, C		164.2, C	
	2	131.7, C		132.4, C	
	3	118.1, CH	5.52, q (7.2)	118.9, CH	5.62, q (6.0)
	4	12.7, CH_3_	1.73, d (7.2)	13.3, CH_3_	1.74, d (6.0)
	2-NH		9.83, s		9.18, s
Dhb	1	164.2, C		164.0, C	
	2	129.3, C		130.6, C	
	3	127.3, CH	5.71, q (7.3)	125.4, CH	5.89, q (5.8)
	4	13.3, CH_3_	1.79, d (7.3)	13.8, CH_3_	1.82, d (5.8)
	2-NH		9.85, s		9.51, s
Tyr	1	171.4, C		172.6, C	4.17, m
	2	56.0, CH	4.26, m	55.6, CH	2.93, d (10.5)
	3a	35.5, CH_2_	3.03, dd (5.2, 14.0),	36.5, CH_2_	
	3b		2.92, dd (10.3, 14.0)		
	4	128.2, C		129.1, C	6.98, d (8.0)
	5/9	129.8, CH	7.03, d (8.4)	130.6, CH	6.58, d (8.0)
	6/8	115.0, CH	6.62, d (8.4)	115.2, CH	
	7	155.9, C		156.1, C	7.62, d (6.0)
	2-NH		8.19, d (6.8)		
	7-OH		9.23, s		
Leu	1	171.3, C			
	2	52.3, CH	4.31, m		
	3a	39.9, CH_2_	1.63, m ^a^		
	3b		1.49, m ^a^		
	4a	24.0, CH	1.63, m ^a^		
	4b				
	5	23.1, CH_3_	0.83, d (6.3)		
	6	21.4, CH_3_	0.80, d (6.3)		
	2-NH		8.00, d (7.6)		
PABA	1	166.1, C			
	2	128.6, C			
	3/7	128.1, CH	7.86, d (8.7)		
	4/6	118.6, CH	7.76, d (8.7)		
	5	141.7, C			
	5-NH		9.83, s		
Thr	1	172.3, C			
	2	58.7, CH	4.40, dd (3.5, 8.3)		
	3	66.6, CH	4.19, m		
	4	20.5, CH_3_	1.13, d (6.4)		
	2-NH		7.94, d (8.3)		

^a^ overlapped.

## Data Availability

The data presented in this study are available in article and supplementary material.

## Supplementary Materials

The following are available online, Table S1: The biosynthetic gene clusters from *B. gladioli* ATCC 10248 predicted by antiSMASH 5.0 and summarized by manual analysis. Table S2: The specificity-conferring code of A domains of bgdd and hgdd. Table S3: Retention times of the amino acids derivatized with Marfey’s reagent (L-FDAA). Table S4: The cytotoxic activities of **1**–**10** against six tumor cell lines and one normal cell line. Table S5: Strains, mutants and plasmids used in this study. Table S6: Primers used in this study. Figure S1: Diagram for construction and verification of BGC 2 on chromosome 2 (Chr2C2) activation and inactivation in ATCC 10248. Figure S2: Diagram for construction and verification of BGC 5 on chromosome 2 (Chr2C5) activation and inactivation in ATCC 10248. Figure S3: Six EICs (911, 1012, 1026, 814, 828 and 786) of crude extract of *B. gladioli* ATCC 10248P_genta_-Chr2C2. Figure S4–S8: HRESIMS spectra, MS/MS fragmentations and structures of compounds **1**–**10**. Figure S9: Swarming and swimming assays of wild type and mutants. Figure S10–S59: NMR spectra for compounds **1**–**10**.

## Sample Availability

Samples of the compounds are available from the authors of H.C. and H.Z.
