# Supplementary material for: Genomics-Driven Activation of Silent Biosynthetic Gene Clusters in Burkholderia gladioli by Screening Recombineering System
Source: Molecules. 2021 Jan 29;26(3):700. doi: 10.3390/molecules26030700 (PMC7866175; doi:10.3390/molecules26030700)
Supplement: Supplementary file 1 [file molecules-26-00700-s001.pdf]

Supplementary Material

Genomics-driven activation of silent biosynthetic gene clusters in *Burkholderia gladioli* by screening recombineering system

Hanna Chen[1], Tao Sun[1], Xianping Bai[1], Jie Yang[1, 2], Fu Yan[2], Lei Yu[3], Qiang Tu[1], Aiying Li[1], Ya-Jie Tang [1], Youming Zhang[1,*], Xiaoying Bian[1,*], Haibo Zhou[1,*]

[1] Helmholtz International Lab for Anti-infectives, Shandong University–Helmholtz Institute of Biotechnology, State Key Laboratory of Microbial Technology, Shandong University, Qingdao 266237, People's Republic of China
[2] Jiangsu Marine Resources Development Research Institute, Lianyungang, 222005, People's Republic of China
[3] Soil and Fertilizer Station of Shandong Province, Jinan 250100, People's Republic of China
* Correspondence: haibozhou@sdu.edu.cn (H.Z.); Tel.: +86 532 67720938; bianxiaoying@sdu.edu.cn (X.B.) ; zhangyouming@sdu.edu.cn (Y.Z.)

Tables

Table S1. The biosynthetic gene clusters from *B. gladioli* ATCC 10248 predicted by antiSMASH 5.0 and summarized by manual analysis.

Gene Clusters	Type	Similar known cluster	Similarity
**Chromosome1**			
BGC1	Hserlactone	-	-
BGC2	T1PKS	Lipopolysaccharide	5%
BGC3	NRPS	Sulfazecin	100%
BGC4	NRPS	-	-
BGC5	NRPS-T1PKS	Orfamide B	30%
BGC6	Terpene	Lasalocid	7%
**Chromosome2**			
BGC1	TransAT-PKS	gladiolin	100%
BGC2	NRPS	-	-
BGC3	Phosphonate	Phosphinothricintripeptide	6%
BGC4	Bacteriocin	-	-
BGC5	NRPS	Pyoverdin	1%
BGC6	NRPS	Gramibactin	46%
BGC7	NRPS	-	-
BGC8	Trans-AT PKS	Lactimidomycin	44%
BGC9	Terpene	-	-
BGC10	Terpene	-	-
BGC11	Terpene	Desotamide	9%
BGC12	NRPS	Icosalide	100%
BGC13	Terpene	Barbamide	41%

"-" indicates unknown.

Table S2. The specificity-conferring code of A domains of *bgdd* and *hgdd*.

Domain	Position											
	235	236	239	278	299	301	322	330	331	517	NRPS predictor 3	Found
BGC2												
A1	D	F	W	N	I	G	M	V	H	K	Thr	Dhb
A2	D	V	Q	Y	I	A	H	V	T	K	Pro	Pro
A3	D	A	W	Q	F	G	L	I	D	K	Gln	Gln
A4	D	L	Y	N	N	A	L	T	Y	K	Ala	Ala
A5	D	I	L	H	L	G	C	T	F	K	N/A	Ala/Val/Ile/Leu
A6	D	A	F	T	V	A	A	I	W	K	Phe	Phe
A7	D	V	Q	Y	V	A	H	V	V	K	Pro	Pro
BGC5												
A1	D	F	W	N	V	G	M	V	H	K	Thr	Dhb
A2	D	F	W	N	V	G	M	V	H	K	Thr	Dhb
A3	D	A	S	T	A	V	G	V	C	K	N/A	Tyr
A4	D	A	M	H	L	G	C	T	F	K	N/A	Leu
A5	D	P	K	N	T	A	N	N	D	K	Pro	PABA

Table S3. Retention times of the amino acids derivatized with Marfey's reagent (L-FDAA).

Amino acid	Configuration	Retention time of hydrolyzed compounds (min)								
		Standard amino acid	**1**	**2**	**3/4**	**5**	**6/7**	**8**	**9**	**10**
Pro	D	11.0	11.0	11.0	11.0					
	L	10.7								
Gln	D	10.3	10.3	10.3	10.3	10.3	10.3	10.3		
	L	10.0								
Ala	D	11.1						11.1		
	L	10.6	10.6	10.6	10.6	10.6	10.6	10.6		
Val	D	12.4	12.4	12.4		12.4				
	L	11.7								
Phe	D	13.0								
	L	12.4	12.4	12.4	12.4	12.4	12.4	12.4		
Leu	D	13.3			13.3		13.3			
	L	12.5							12.5	
Tyr	D	11.3							11.3	11.3
	L	11.0								
Ile	D	33.2			33.2		33.2			
	L	29.8								
allo-Ile	D	33.4								
	L	29.9								
Thr	D	21.9								
	L	18.9		18.9	18.9				18.9	
allo-Thr	D	20.3								
	L	19.1								

Table S4. The cytotoxic activities of **1-10** against six tumor cell lines and one normal cell line.

**Compound**	HCT116	MCF-7	Hela	HepG-2	A549	K562	293T
**1**	>20	>20	>20	>20	>20	>20	29.41±0.85
**2**	>20	>20	>20	>20	>20	>20	>40
**3/4**	>20	>20	>20	>20	>20	>20	28.4±0.53
**5**	>20	>20	>20	>20	>20	>20	24.95±0.96
**6/7**	>20	>20	>20	>20	>20	>20	32.76±0.74
**8**	>20	>20	>20	>20	>20	>20	>40
**9**	>20	>20	>20	>20	>20	>20	36.12±0.81
**10**	>20	>20	>20	>20	>20	>20	33.90±0.75
adriamycin	0.73±0.05	0.86±0.07	0.61±0.07	0.68±0.001	0.61±0.03	0.57±0.02	1.47±0.15

Table S5. Strains, mutants and plasmids used in this study.

**Strains**	**Description**	**Source**
Burkholderia gladioli ATCC 10248	Wild type, plant pathogen	CGMCC
**Plasmids**		
pBBR1-Rha-Redγ-BAS-km	pBBR1 replicon, km^R, recombinase Redγ-BAS under the control of rhamnose promoter	[1]
pBBR1-Rha-Redγβα-km	pBBR1 replicon, km^R, recombinase Redγβα under the control of rhamnose promoter	[2]
pBBR1-Rha-Redγ-Redαβ7029-km	pBBR1 replicon, km^R, recombinase Redγ-Redβα7029 under the control of rhamnose promoter	[2]
RK2-apra-cm	Rk2 replicon, $apra^R$, cm^R	Our lab
R6K-lox71-genta-lox66-FleQ	R6K replicon, $genta^R$, PCR templates to amplify gentamicin resistance gene	Our lab
**Mutants**		
B. gladioli ATCC 10248*Δgbn*	The fragment (468577-469853) of gladiolin gene cluster was replaced by apramycin resistance gene in ATCC 10248	This study
B. gladioli ATCC 10248*ΔgbnΔChr2C2*	The region (763367-764614) of Chr2C2 was replaced by gentamicin resistance gene, and the fragment (468577-469853) of gladiolin gene cluster was replaced by apramycin resistance gene in ATCC 10248	This study
B. gladioli ATCC 10248*Δgbn*P_{genta}*-Chr2C2*	The P_{genta} promoter and gentamicin resistance gene was inserted upstream of core biosynthetic region (761640-761899) of Chr2C2, and the fragment (468577-469853) of gladiolin gene cluster was replaced by apramycin resistance gene in ATCC 10248	This study
B. gladioli ATCC 10248*ΔgbnΔChr2C5*	The region (1519820-1521082) of Chr2C5 was replaced by gentamicin resistance gene, and the fragment (468577-469853) of gladiolin gene cluster was replaced by apramycin resistance gene in ATCC 10248	This study
B. gladioli ATCC 10248*Δgbn*P_{genta}*-Chr2C5*	The P_{genta} promoter and gentamicin resistance gene was inserted upstream of core biosynthetic region (1512547-1522619) of Chr2C5, and the fragment (468577-469853) of gladiolin gene cluster was replaced by apramycin resistance gene in ATCC 10248	This study

Table S6. Primers used in this study.

Primers	Primer sequences (5′-3′)
Chr2gbnKO-10248-50apra-S	*TCGAGGCAGGCAGCTCCGGCGCGCGGCCCGCTTCCGTCGCCGGTGCGATG*TCAGCCAATCGACTGGCGA
Chr2gbnKO-10248-50apra-A	*CTGCGCCGGCGTCTTGGCCGACAGCACCAGGTCGAGCGGCTCGCGCGATG*ACGCTCAGTGGAACGAGGTT
Chr2gbnKO-10248-75apra-S	*GCCAAGACCGGAAACGCCCCGCGCG*TCGAGGCAGGCAGCTCCGG
Chr2gbnKO-10248-75apra-A	*GCAAGGCGGCGGCACGCGCCCGCAA*CTGCGCCGGCGTCTTGGCC
Chr2gbnKO-10248-100apra-S	*GGCTGTTGAACCTGCCGCTCCCGTCGCCAAGACCGGAAACGCCCCGCGCG*TCGAGGCAGGCAGCTCCGG
Chr2gbnKO-10248-100apra-A	*TAAGCACCGTCCTCGATCCGCTCCAGCAAGGCGGCGGCACGCGCCCGCAA*CTGCGCCGGCGTCTTGGCC
Chr2C2-10248-Pgenta-S	*GTGCTGAGACCAGCGCATCCGAGACACGCCGCTGCGCGAGGGCCATGCCG*GAAGGCACGAACCCAGTTGA
Chr2C2-10248-Pgenta-A	*TGCGAACTCGTCAGGCCGTGGTGCTCTTGATGCGAAGTGGGATTGTTCAT*AATCTGTACCTCCTTAAGTGA
Chr2C2KO-10248-genta-S	*TCGAGGCGCAGGCCGCACGCGTGCCCGATGCGATCGCGGTGCTGCATGAG*AATCTGTACCTCCTTAAGTGA
Chr2C2KO-10248-genta-A	*TCGCCCGTGTAGTAGGCCGCGAGGCGCTTGTCGCCGGGCGCGTCCTCGCG*GAAGGCACGAACCCAGTTGA
Chr2C5-10248-Pgenta-S	*TTGCGCGGCCGTCAACGGATACACTTCGCCCAGCAGGACTGTCTGGTTCAT*AATCTGTACCTCCTTAAGTGA
Chr2C5-10248-Pgenta-A	*CGGCCGGCGCGACGGCGGTGTAAGGGCGTGCCTGGCGCACGTCATCGAGA*GAAGGCACGAACCCAGTTGA
Chr2C5KO-10248-genta-S	*TGTGTCCGAAGCCGCCTCGGCATCGTCCACAACGAAATACGCGACGAGCC*GAAGGCACGAACCCAGTTGA
Chr2C5KO-10248-genta-A	*GTTCGAGGCCCGTGCGGCGCAAACGCCGGACGCGGTCGCGCTGGAATTCG*AATCTGTACCTCCTTAAGTGA
Chr2C2KO-check-S	CACTTCATCCACAACCAGGC
Chr2C2KO-check-A	GTCCAGATGCACGAAGGC
Chr2C2Pgenta-check-S	GCCTGCTGAATTCGACTCAT
Chr2C2Pgenta-check-A	TAGATCCTGCCCAGCGATTC
Chr2C5KO-check-S	CGTACTGCTCAGCGTCTACC
Chr2C5KO-check-A	AGGAGGAAGTCGAGGCGATC
Chr2C5Pgenta-check-S	ACCAGCAGGTTCACGGCGC
Chr2C5Pgenta-check-A	CGATCACGGCGCTGTCCGAT
Chr2gbnKO-check-S	CTGTTGAACCTGCCGCTC
Chr2gbnKO-check-A	GAACGGATAGGTCGGCAGG

Italic indicates homology arms.

References

1. Yin, J.; Zheng, W.; Gao, Y.; Jiang, C.; Shi, H.; Diao, X.; Li, S.; Chen, H.; Wang, H., Li, R.; Li, A.; Xia, L.; Yin, Y.; Stewart, A.F.; Zhang, Y.; Fu J. Single-Stranded DNA-Binding Protein and Exogenous RecBCD Inhibitors Enhance Phage-Derived Homologous Recombination in Pseudomonas. *iScience*. **2019**, *14*, 1–14.
2. Wang, X.; Zhou, H.; Chen, H.; Jing, X.; Zheng, W.; Li, R.; Sun, T.; Liu, J.; Fu, J.; Huo, L.; Li, Y.; Ding, X.; Mu¨ller, R.; Bian, X.; Zhang, Y. Discovery of recombinases enables genome mining of cryptic biosynthetic gene clusters in Burkholderiales species. *Proc. Natl. Acad. Sci.* **2018**, *115*, E4255–E4263.

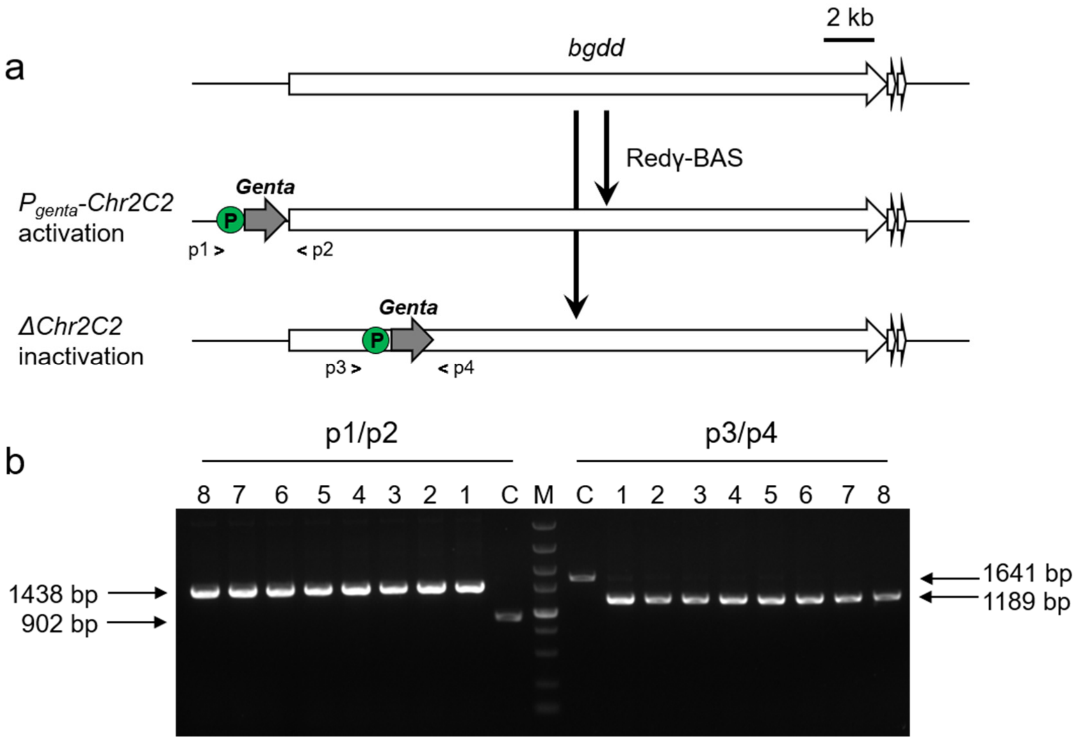

Figure S1. Diagram for construction and verification of BGC 2 on chromosome 2 (Chr2C2) activation and inactivation in ATCC 10248.

a. Diagram for construction of BGC 2 activation (P_{genta}-Chr2C2) and inactivation (ΔChr2C2) using Redγ-BAS recombinases.

b. PCR verification of the activation and inactivation of Chr2C2 of ATCC 10248 using p1/p2 and p3/p4. p1: Chr2C2KO-check-S, p2: Chr2C2KO-check-A, p3: Chr2C2Pgenta-check-S, p4: Chr2C2Pgenta-check-A. M: DL5000 DNA ladder, C: ATCC 10248. size of PCR product: 1438 bp (mutants, p1/p2), 1189 bp (mutants, p3/p4).

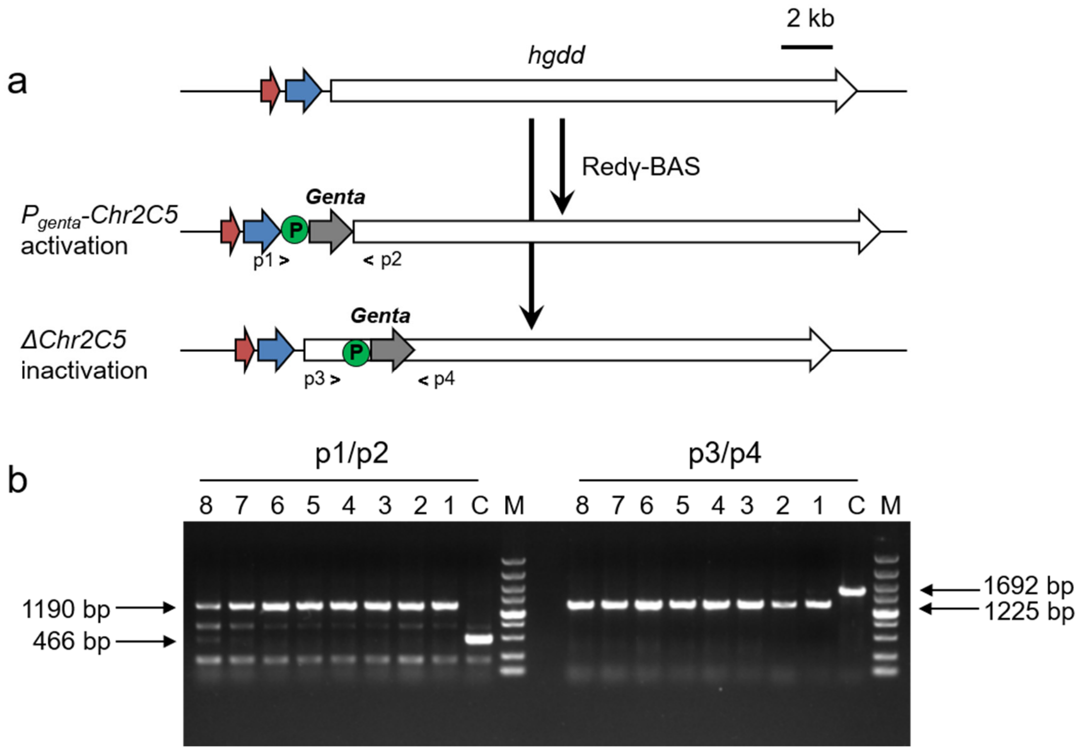

Figure S2. Diagram for construction and verification of BGC 5 on chromosome 2 (Chr2C5) activation and inactivation in ATCC 10248.

a. Diagram for construction of BGC 5 activation (P_{genta}-Chr2C5) and inactivation (ΔChr2C5) using Redγ-BAS recombinases.

b. PCR verification of the activation and inactivation of Chr2C5 of ATCC 10248 using p1/p2 and p3/p4. p1: Chr2C5KO-check-S, p2: Chr2C5KO-check-A, p3: Chr2C5Pgenta-check-S, p4: Chr2C5Pgenta-check-A. M: DL5000 DNA ladder, C: ATCC 10248. size of PCR product: 1190 bp (mutants, p1/p2), 1225 bp (mutants, p3/p4).

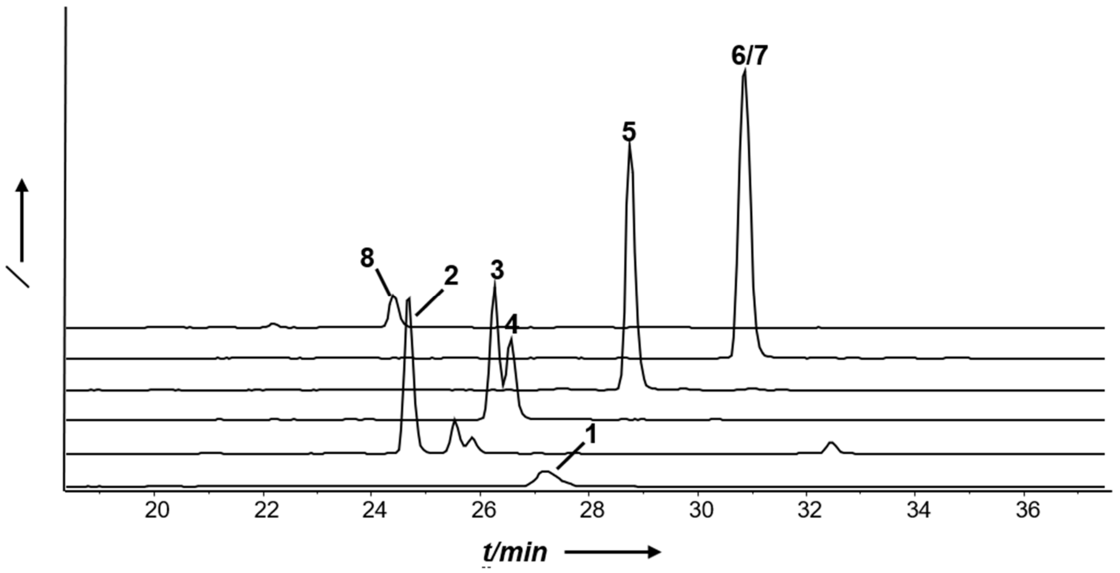

Figure S3. Six EICs (911, 1012, 1026, 814, 828 and 786) of crude extract of *B. gladioli* ATCC 10248*ΔgbnP*$_{genta}$*-Chr2C2*. Compounds **1**-**8** were purified and their structures were elucidated by NMR, HRESIMS and feeding experiments with labelled precursors.

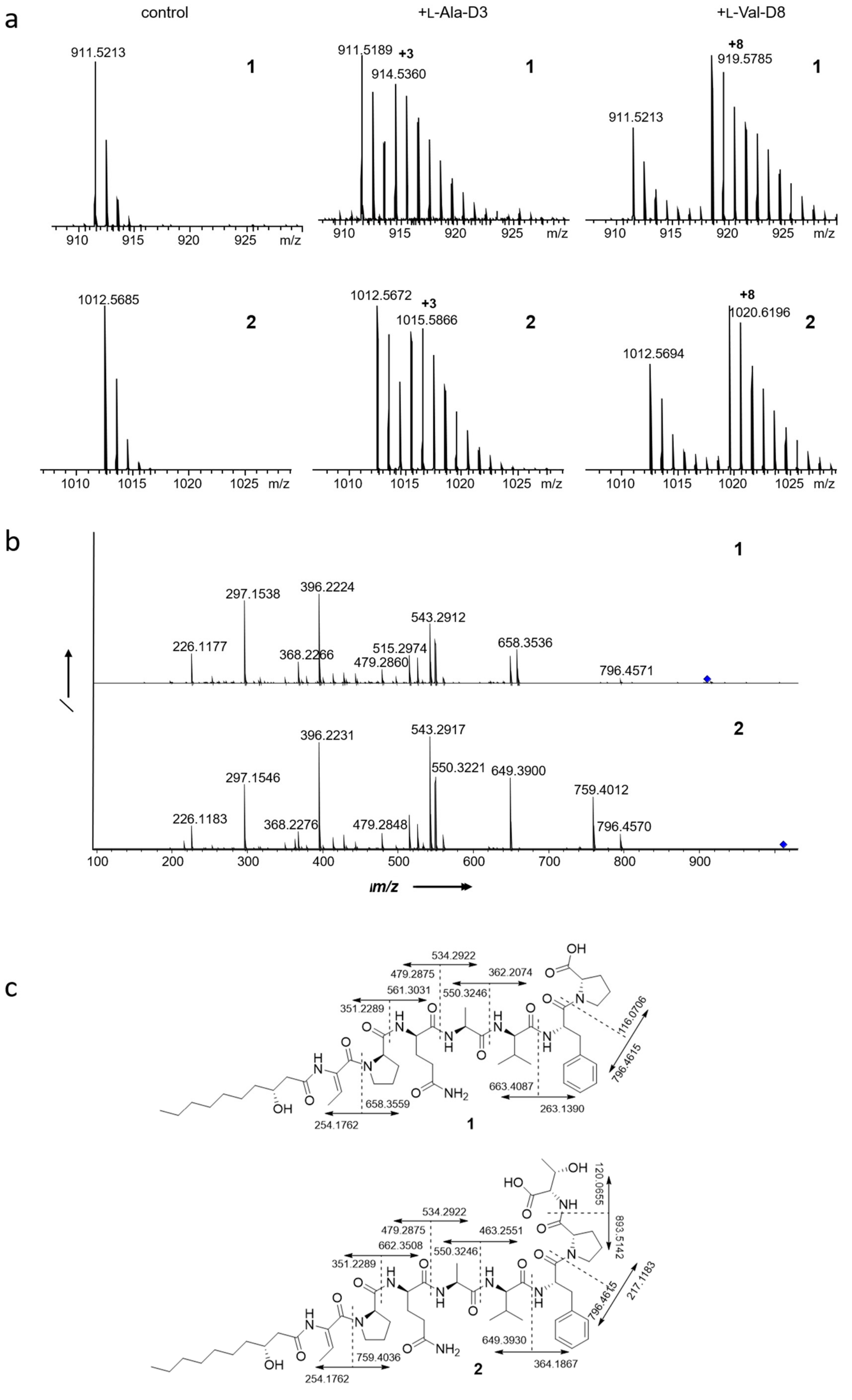
a
control
+L-Ala-D3
+L-Val-D8
911.5213
1
911.5189
+3
914.5360
+8
919.5785
1012.5685
2
1012.5672
1015.5866
1012.5694
1020.6196
m/z
b
297.1538
396.2224
543.2912
226.1177
368.2266
515.2974
479.2860
658.3536
796.4571
396.2231
543.2917
550.3221
649.3900
297.1546
759.4012
226.1183
368.2276
479.2848
796.4570
c
534.2922
479.2875
362.2074
561.3031
550.3246
351.2289
116.0706
796.4615
663.4087
263.1390
658.3559
254.1762
120.0655
893.5142
463.2551
662.3508
217.1183
649.3930
364.1867
759.4036

Figure S4. HRESIMS spectra, MS/MS fragmentations and structures of compound **1** and **2**. **a**, HRESIMS spectra of **1** and **2** after HPLC-MS analysis of culture extracts from feeding experiments with labelled L-Ala and L-Val. **1** and **2** both contained one Ala and one Val. **b**, MS/MS fragmentations of **1** and **2**. **c**, Structure and calculated fragmentations of **1** and **2**.

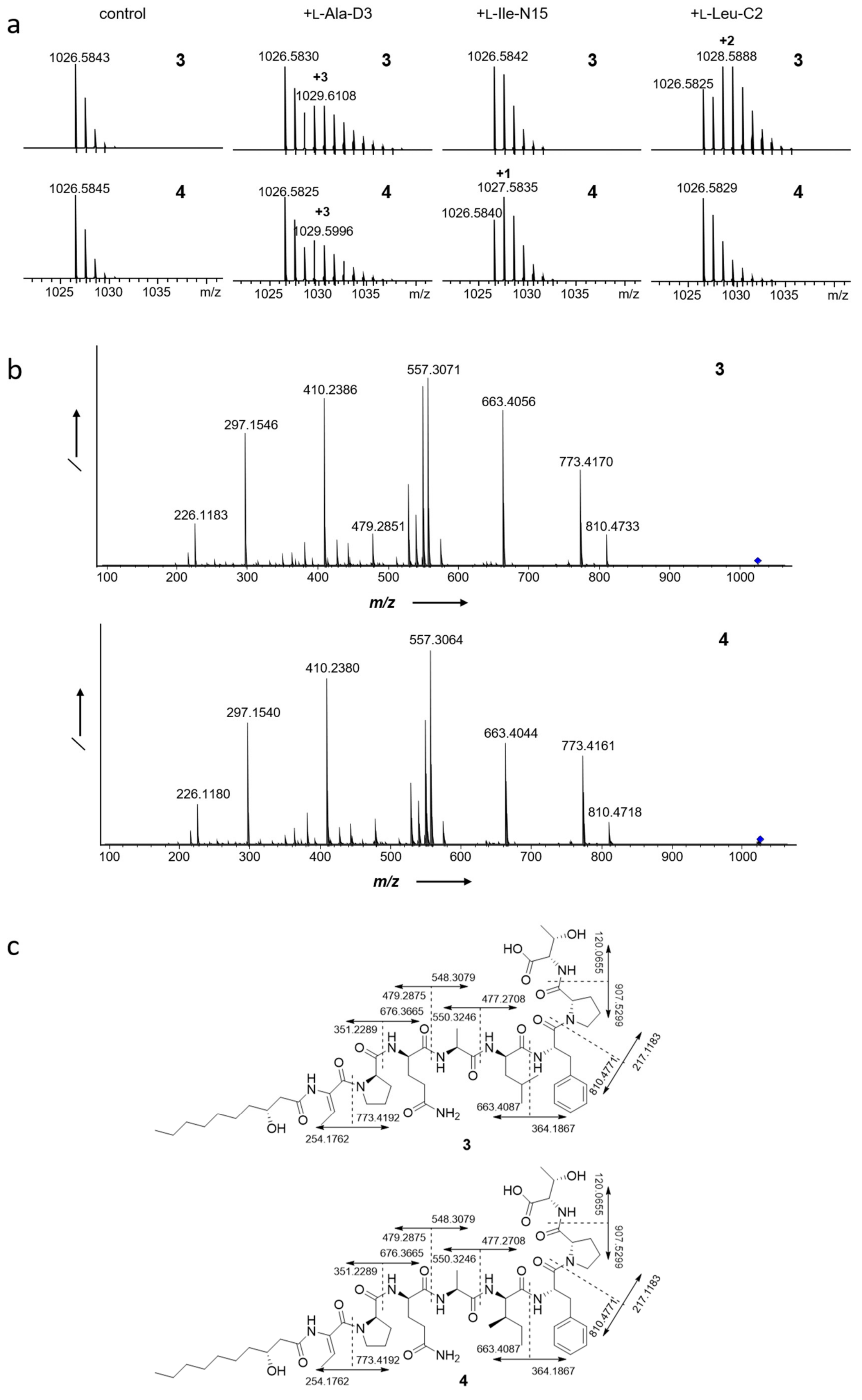
a
control
+L-Ala-D3
+L-Ile-N15
+L-Leu-C2
1026.5843
3
1026.5830
+3
1029.6108
1026.5842
+2
1028.5888
1026.5825
1026.5845
4
1026.5825
+3
1029.5996
+1
1027.5835
1026.5840
1026.5829
1025
1030
1035
m/z
b
557.3071
410.2386
663.4056
297.1546
773.4170
226.1183
479.2851
810.4733
m/z
557.3064
410.2380
297.1540
663.4044
773.4161
226.1180
810.4718
c
120.0655
907.5299
548.3079
479.2875
477.2708
676.3665
550.3246
351.2289
217.1183
810.4771
663.4087
773.4192
364.1867
254.1762

Figure S5. HRESIMS spectra, MS/MS fragmentations and structures of compound **3** and **4**. **a**, HRESIMS spectra of **3** and **4** after HPLC-MS analysis of culture extracts from feeding experiments with labelled L-Ala, L-Ile and L-Leu. **3** contained one Ala and one Leu. **4** contained one Ala and one Ile. **b**, MS/MS fragmentations of **3** and **4**. **c**, Structure and calculated fragmentations of **3** and **4**.

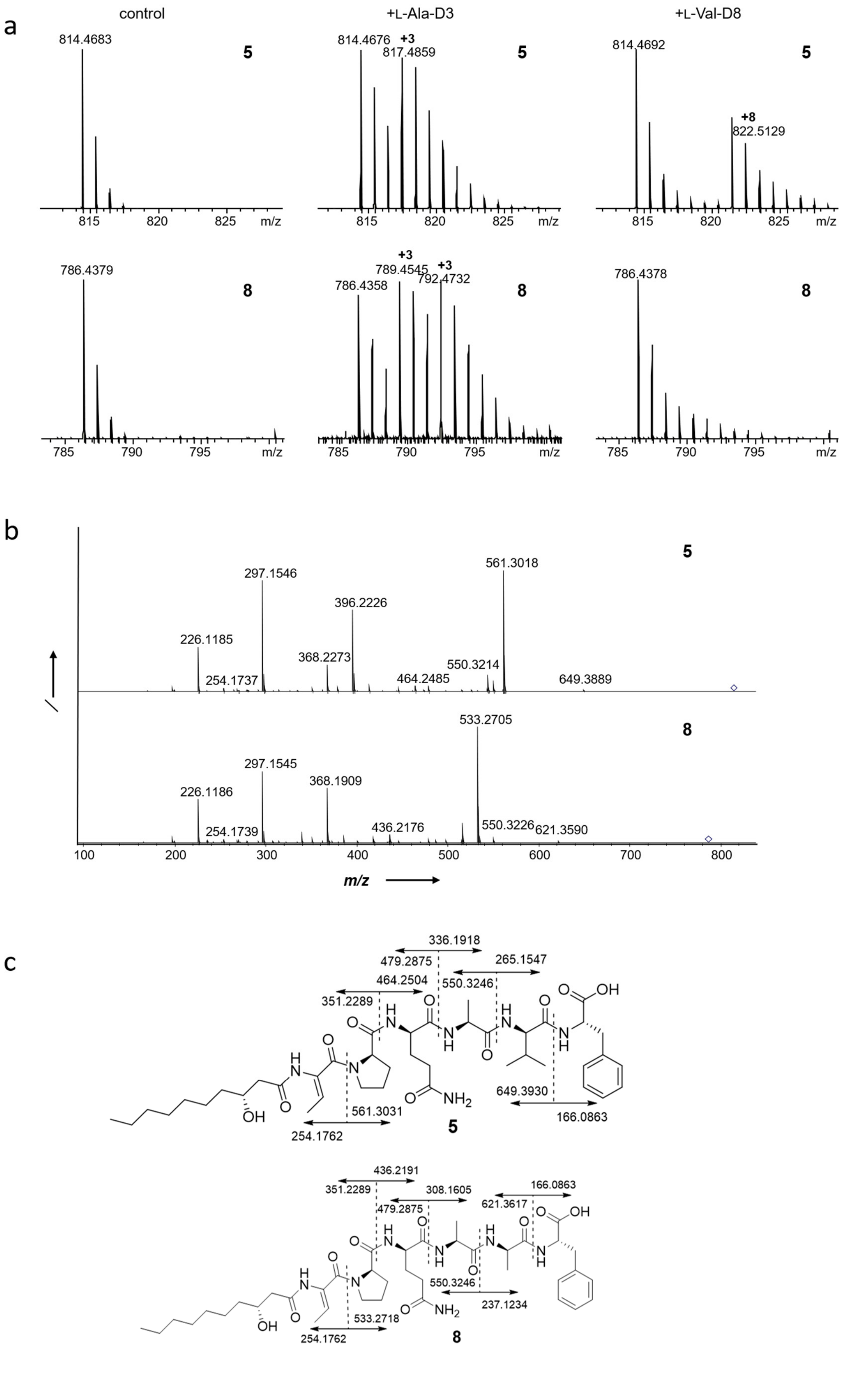
a
control
+L-Ala-D3
+L-Val-D8
814.4683
5
814.4676
+3
817.4859
5
814.4692
5
+8
822.5129
815
820
825
m/z
786.4379
8
+3
789.4545
+3
792.4732
786.4358
8
786.4378
8
785
790
795
m/z
b
297.1546
561.3018
5
396.2226
226.1185
368.2273
550.3214
254.1737
464.2485
649.3889
533.2705
8
297.1545
368.1909
226.1186
436.2176
550.3226
621.3590
254.1739
100
200
300
400
500
600
700
800
m/z
c
336.1918
479.2875
265.1547
464.2504
550.3246
351.2289
649.3930
561.3031
166.0863
254.1762
5
436.2191
351.2289
308.1605
166.0863
479.2875
621.3617
550.3246
237.1234
533.2718
254.1762
8

Figure S6. HRESIMS spectra, MS/MS fragmentations and structures of compounds **5** and **8**. **a**, HRESIMS spectra of **5** and **8** after HPLC-MS analysis of culture extracts from feeding experiments with labelled L-Ala and L-Val. **5** contained one Ala and one Val. **8** contained two Ala. **b**, MS/MS fragmentations of **5** and **8**. **c**, Structure and calculated fragmentations of **5** and **8**.

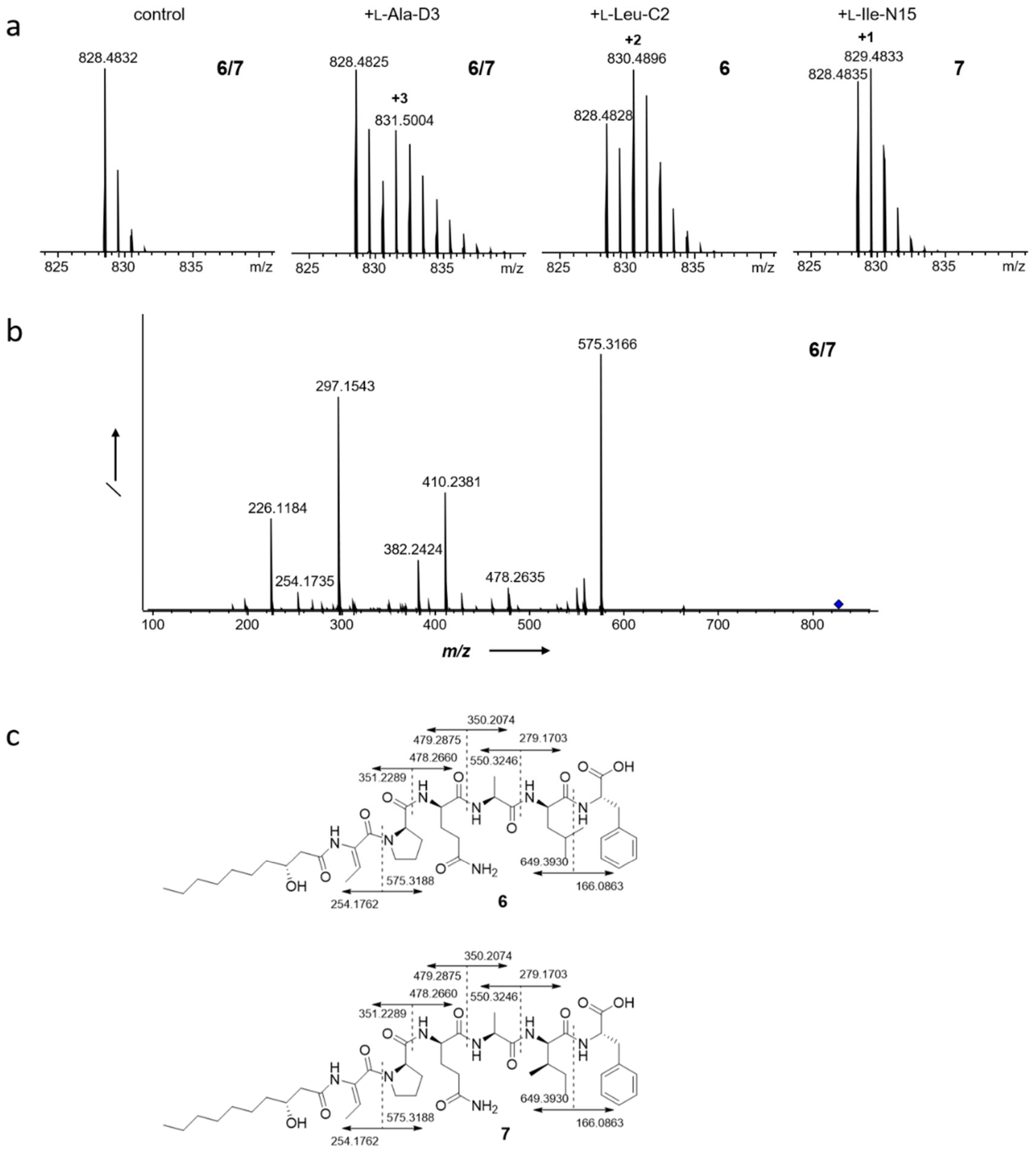

Figure S7. HRESIMS spectra, MS/MS fragmentations and structures of compound **6** and **7**. **a**, HRESIMS spectra of **6** and **7** after HPLC-MS analysis of culture extracts from feeding experiments with labelled L-Ala, L-Ile and L-Leu. **6** contained one Ala and one Leu. **7** contained one Ala and one Ile. **b**, MS/MS fragmentations of **6** and **7**. **c**, Structure and calculated fragmentations of **6** and **7**.

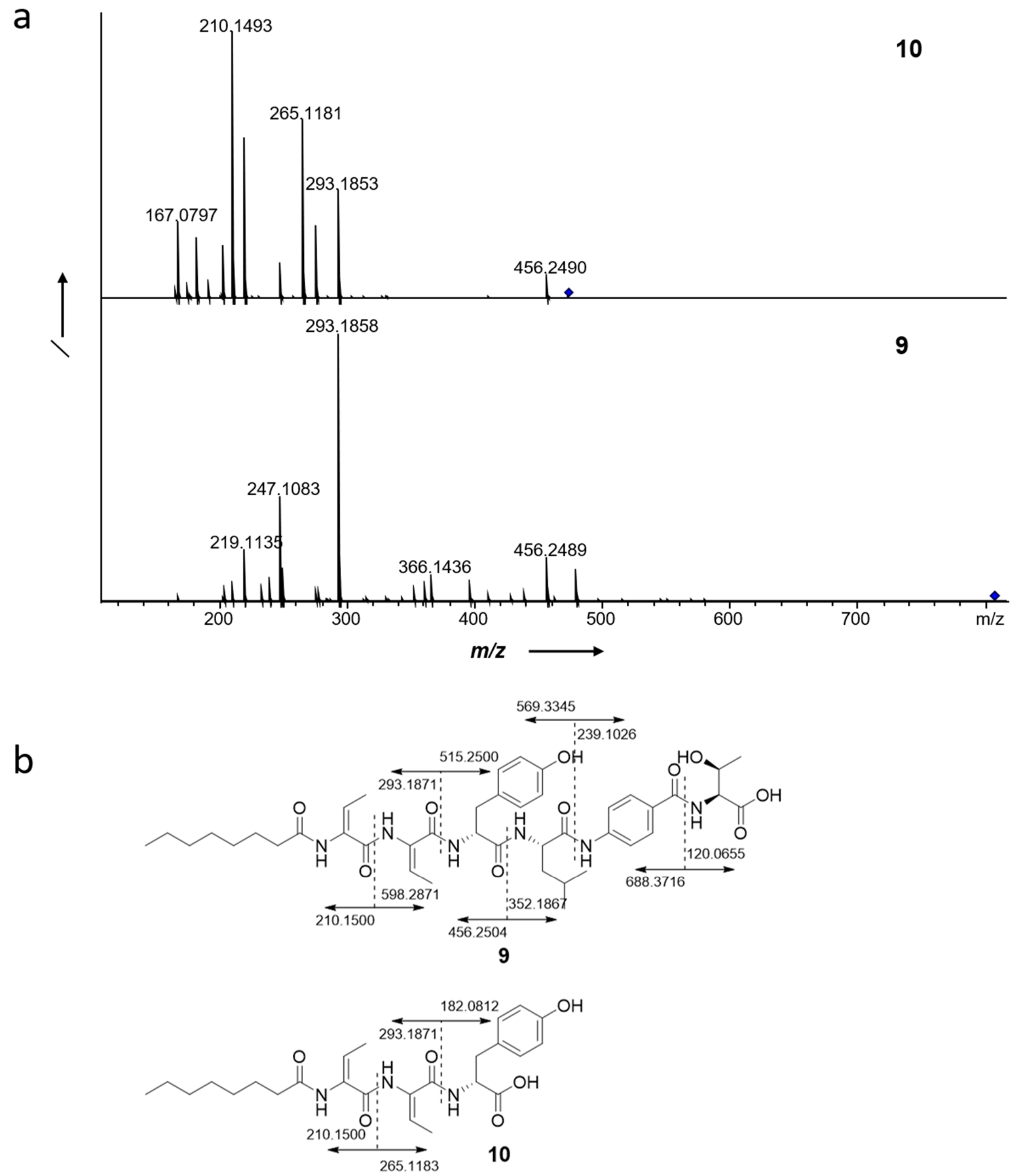

Figure S8. MS/MS fragmentations and structures of compound **9** and **10**. **a**, MS/MS fragmentations of **9** and **10**. **b**, Structures and calculated fragmentations of **9** and **10**.

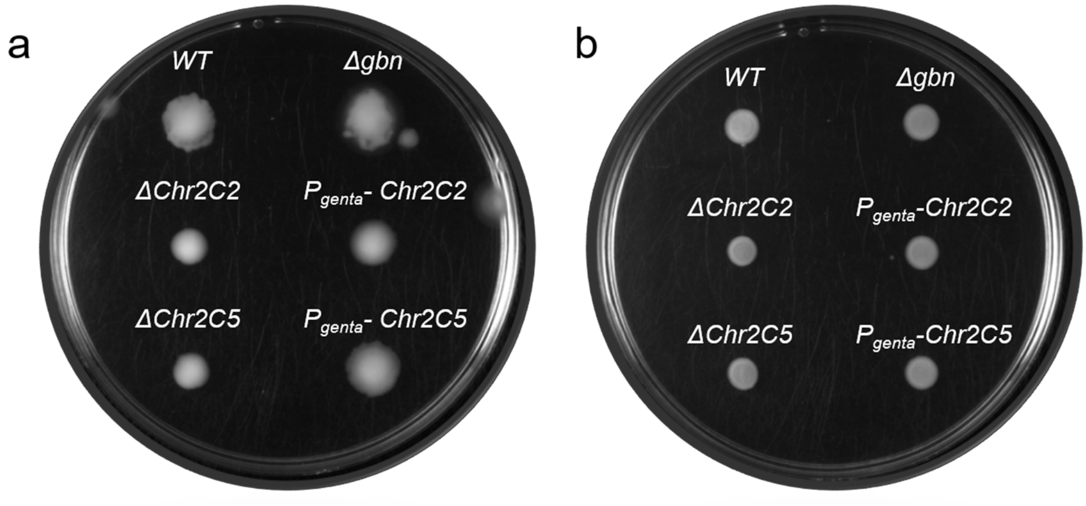

Figure S9. Swarming and swimming assays of wild type and mutants.

a, Swarming phenotypes of wild type, mutant *Δgbn*, activation mutants and inactivation mutants on the CYMG plates with 0.5% agar. **b**, Swimming phenotypes of wild type, mutant *Δgbn*, activation mutants and inactivation mutants on the CYMG plates with 0.25% agar. *WT*: wild type ATCC 10248, *Δgbn*: mutant ATCC 10248*Δgbn*, P_{genta}-*BGC2*: activation mutant ATCC 10248*ΔgbnP*$_{genta}$-*Chr2C2*, *ΔChr2C2*: inactivation mutant ATCC 10248*ΔgbnΔChr2C2*, P_{genta}-*Chr2C5*: activation mutant ATCC 10248*ΔgbnP*$_{genta}$-*Chr2C5*, and *ΔChr2C5*: inactivation mutant ATCC 10248*ΔgbnΔChr2C5*.

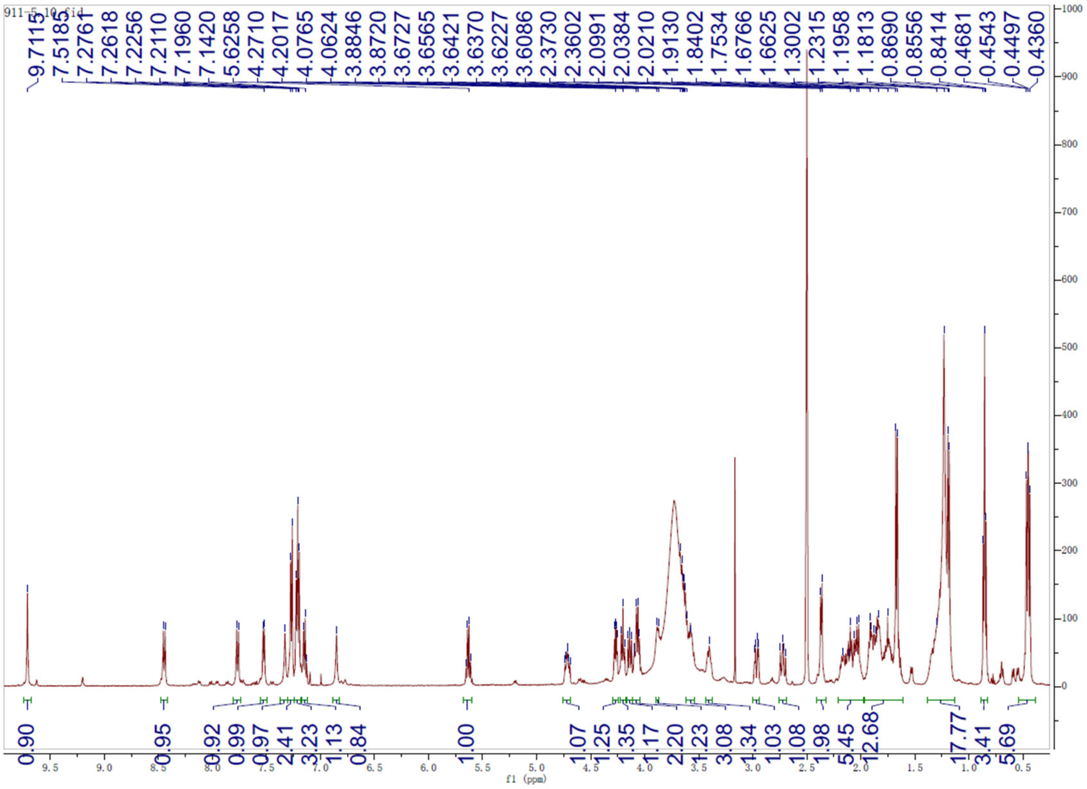

Figure S10. ^{1}H NMR spectrum of burriogladiodin A (**1**) in DMSO-d_6

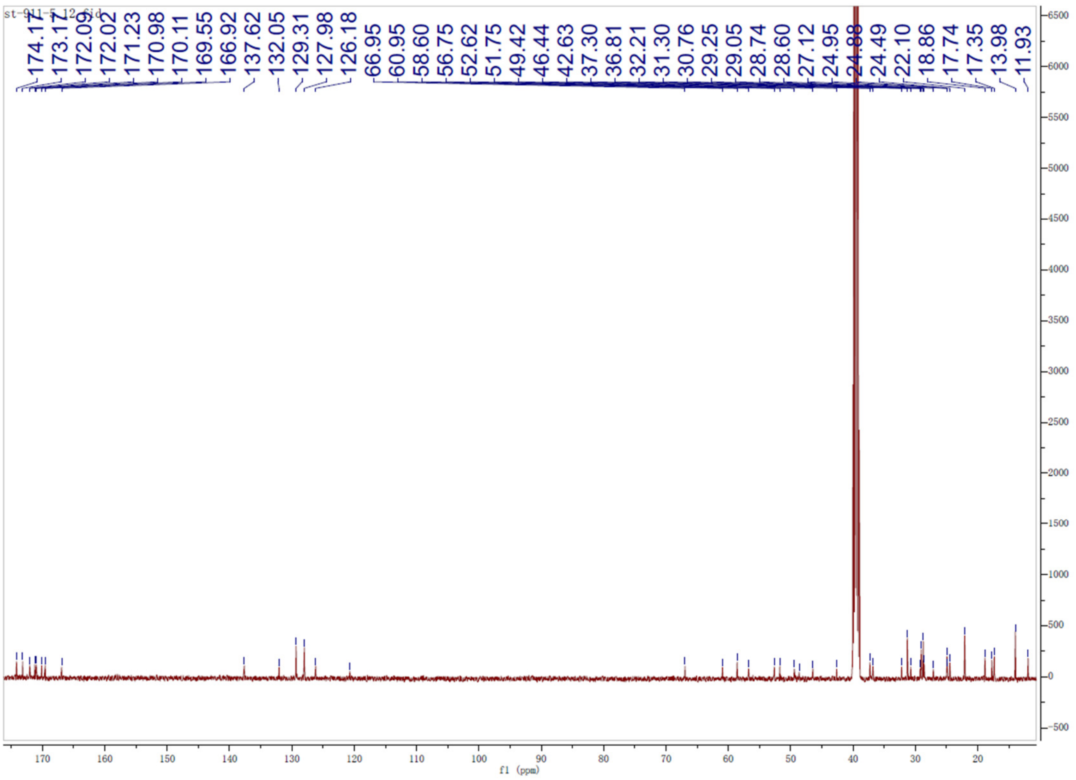

Figure S11. ^{13}C NMR spectrum of burriogladiodin A (**1**) in DMSO-d_6

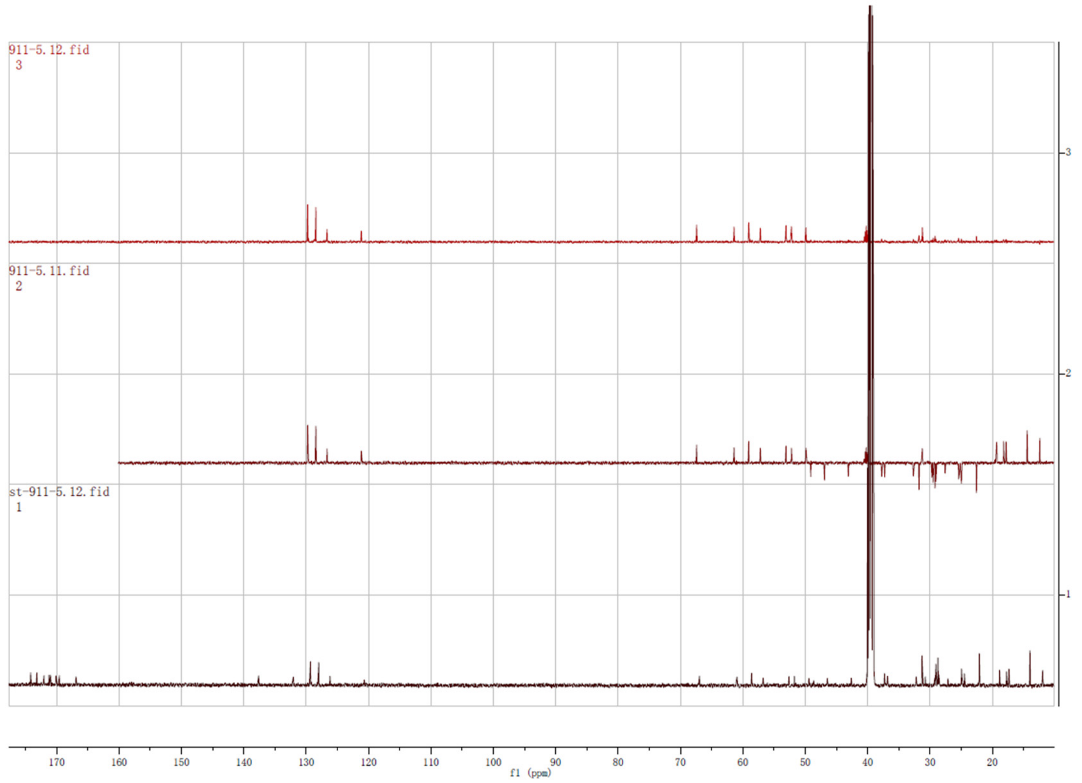

Figure S12. DEPT spectrum of burriogladiodin A (**1**) in DMSO-*d*6

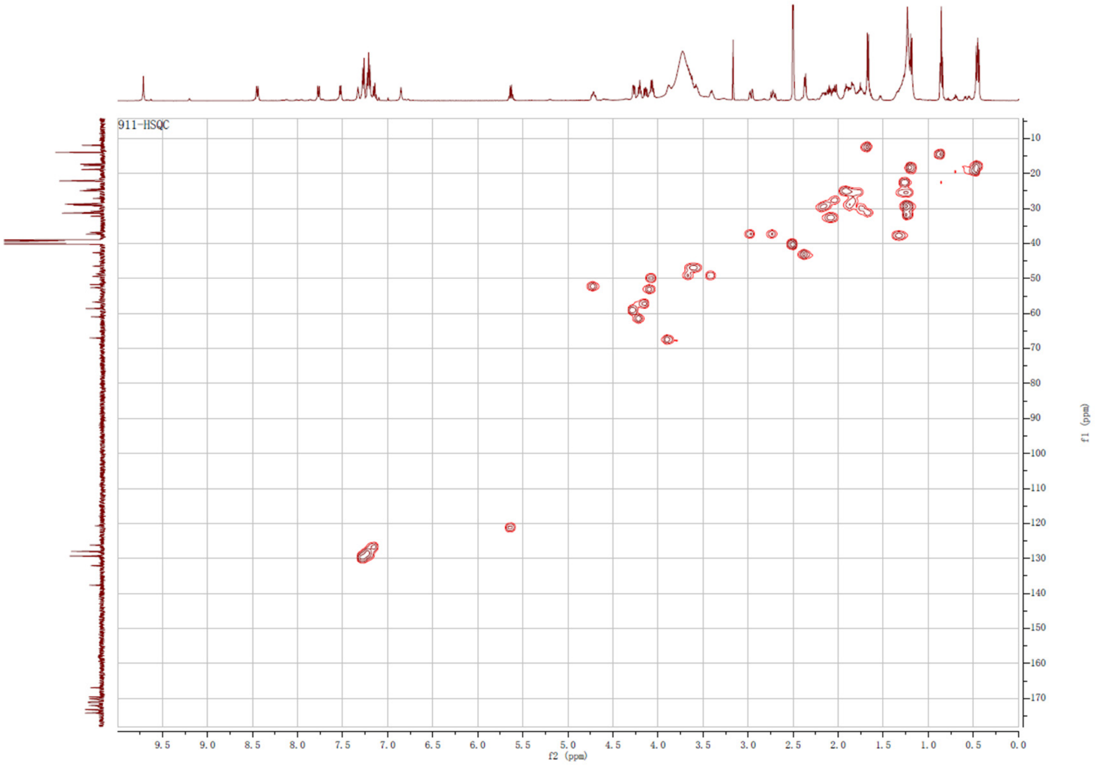

Figure S13. HSQC spectrum of burriogladiodin A (**1**) in DMSO-*d*6

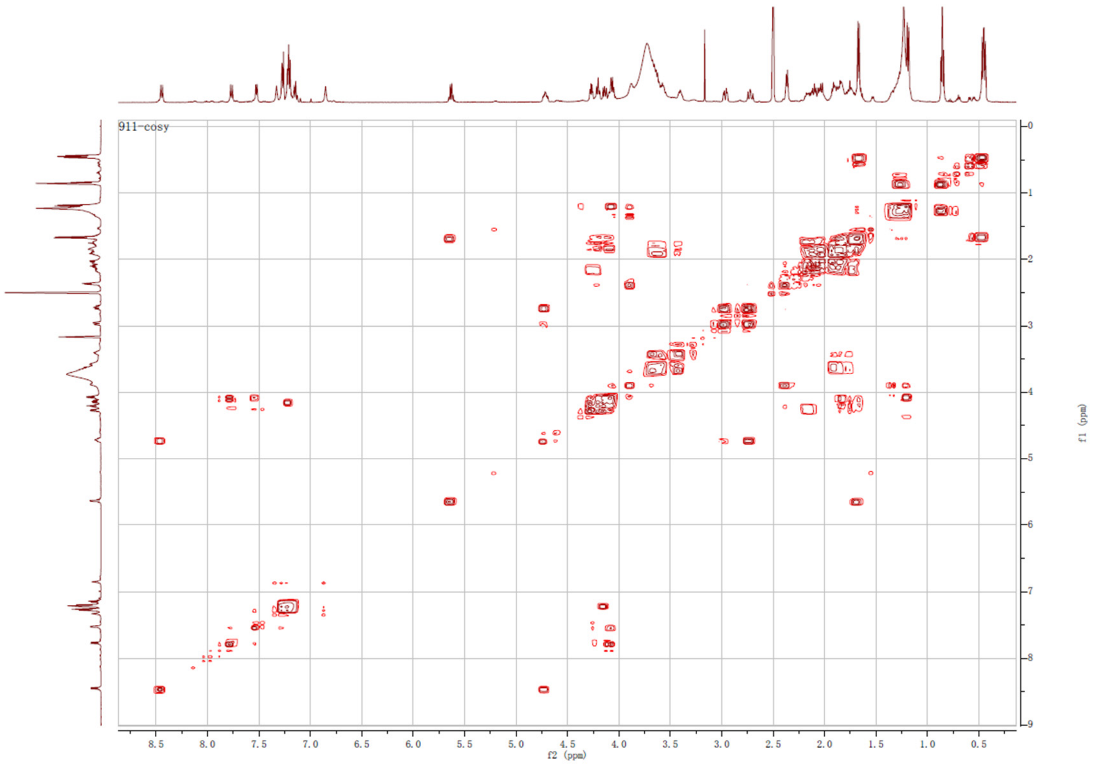

Figure S14. ^{1}H-^{1}H COSY spectrum of burriogladiodin A (**1**) in DMSO-*d*$_6$

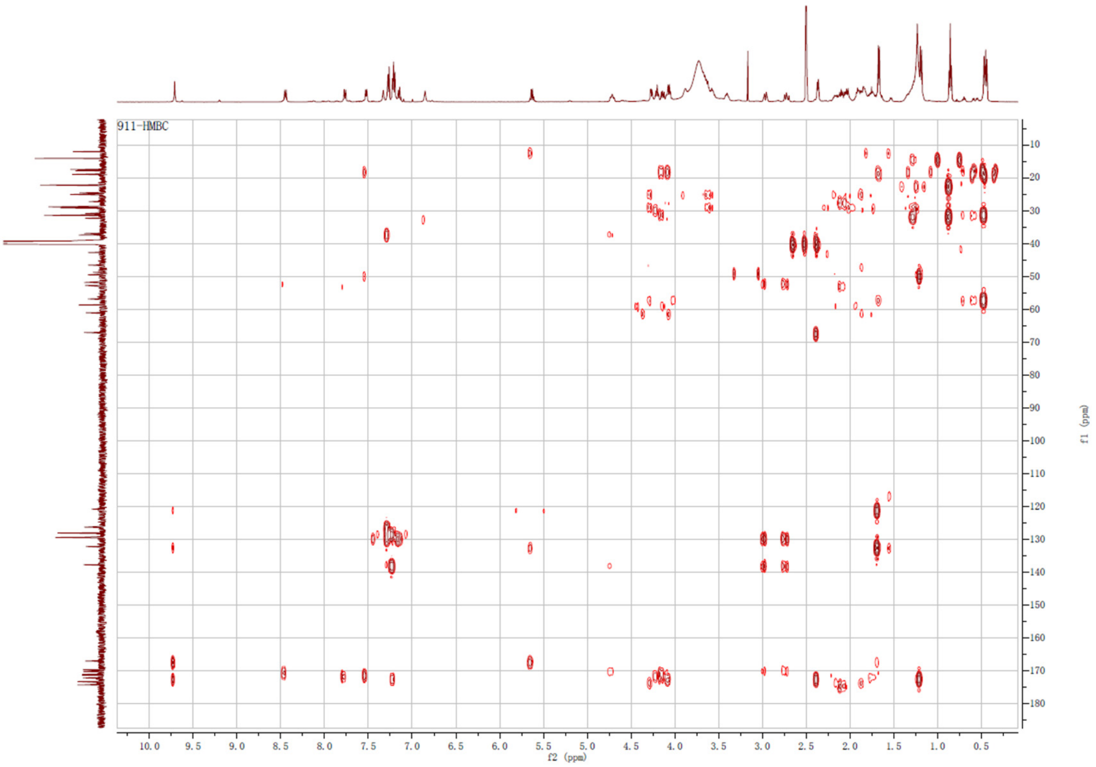

Figure S15. HMBC spectrum of burriogladiodin A (**1**) in DMSO-*d*$_6$

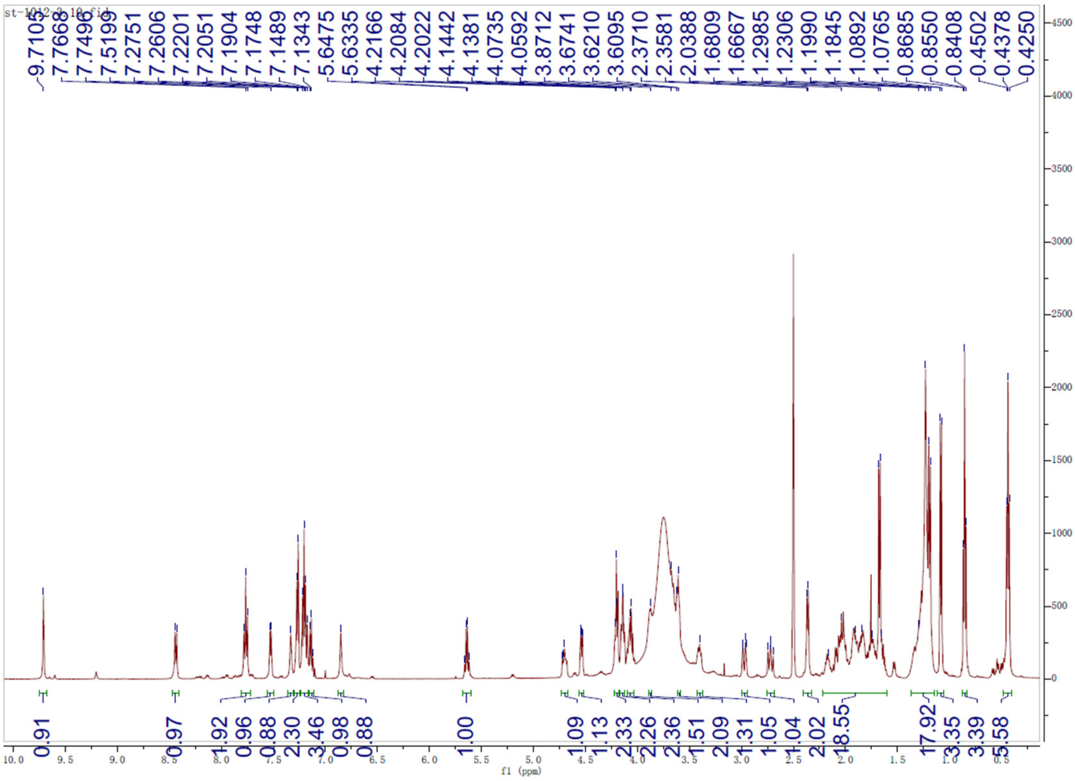

Figure S16. ^{1}H NMR spectrum of burriogladiodin B (**2**) in DMSO-*d*6

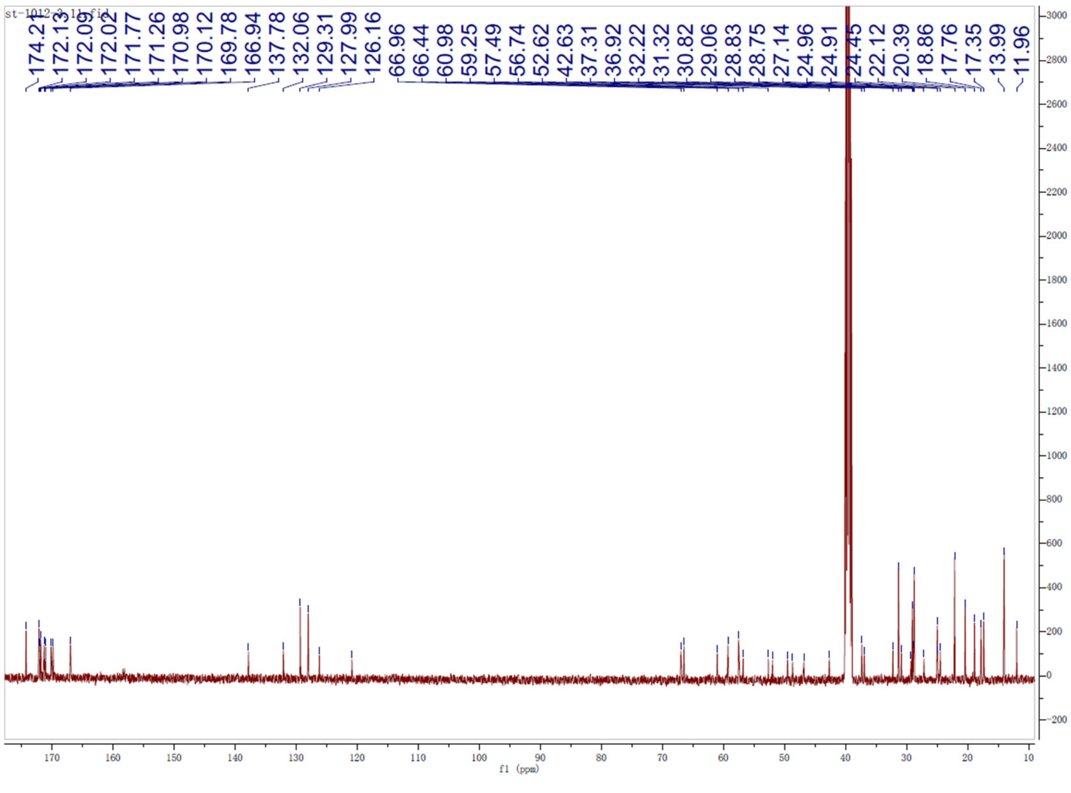

Figure S17. ^{13}C NMR spectrum of burriogladiodin B (**2**) in DMSO-*d*6

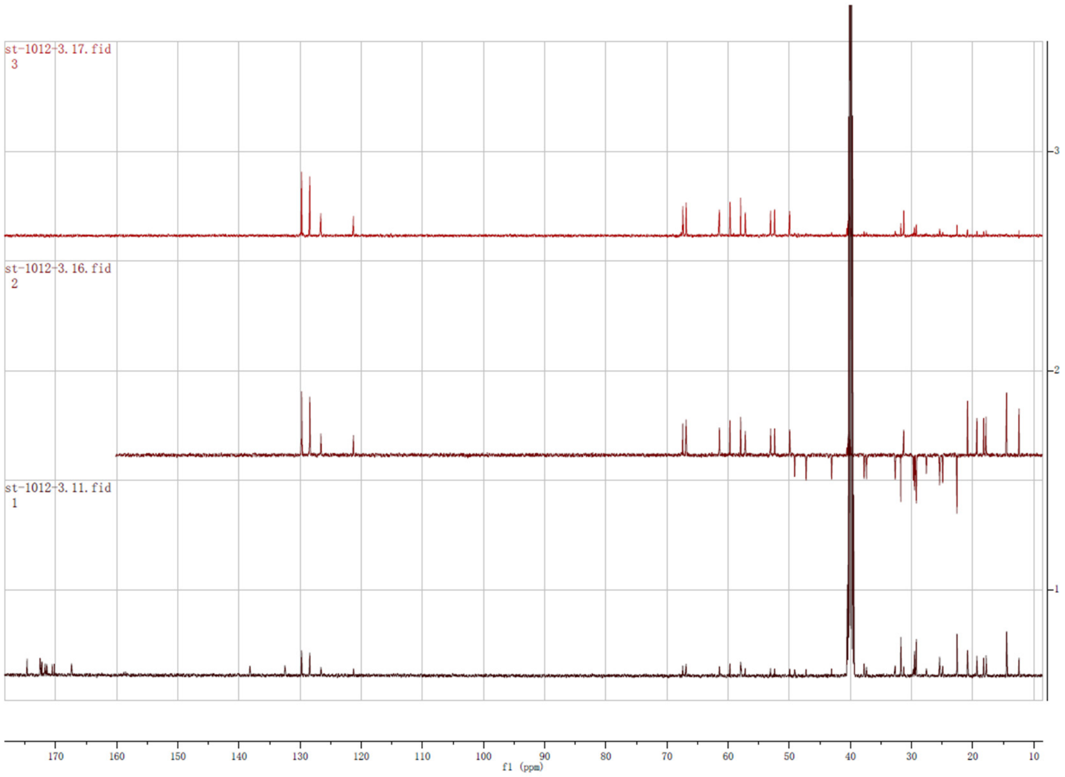

Figure S18. DEPT spectrum of burriogladiodin B (**2**) in DMSO-d_6

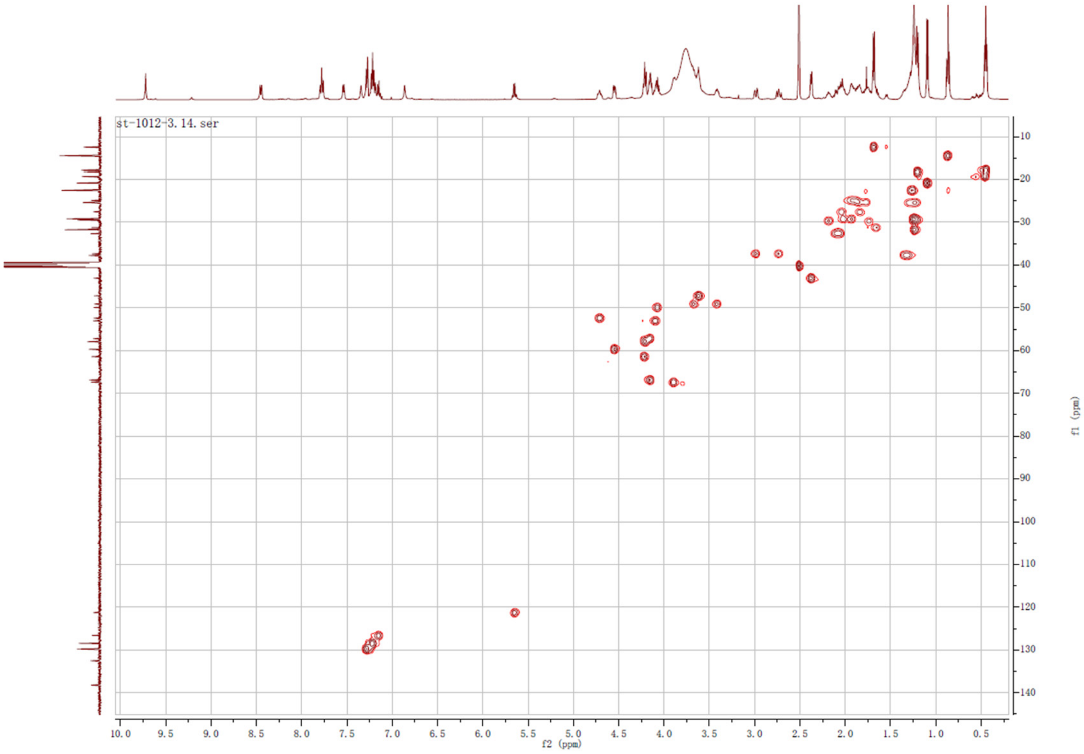

Figure S19. HSQC spectrum of burriogladiodin B (**2**) in DMSO-d_6

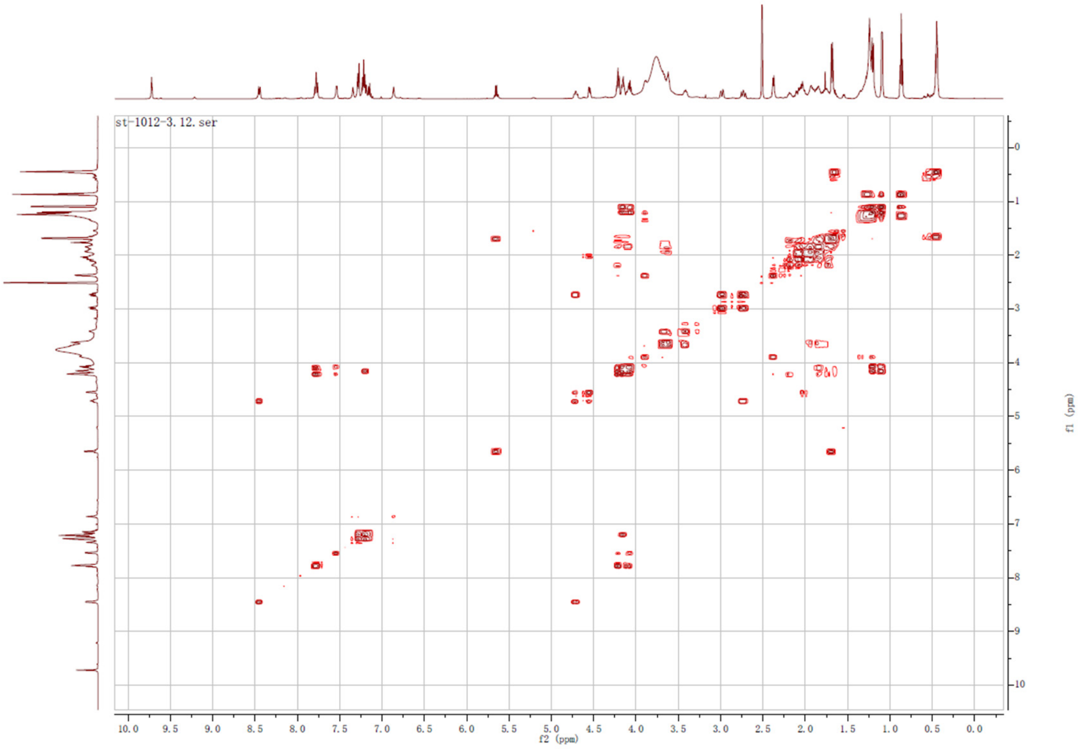

Figure S20. ¹H-¹H COSY spectrum of burriogladiodin B (**2**) in DMSO-d_6

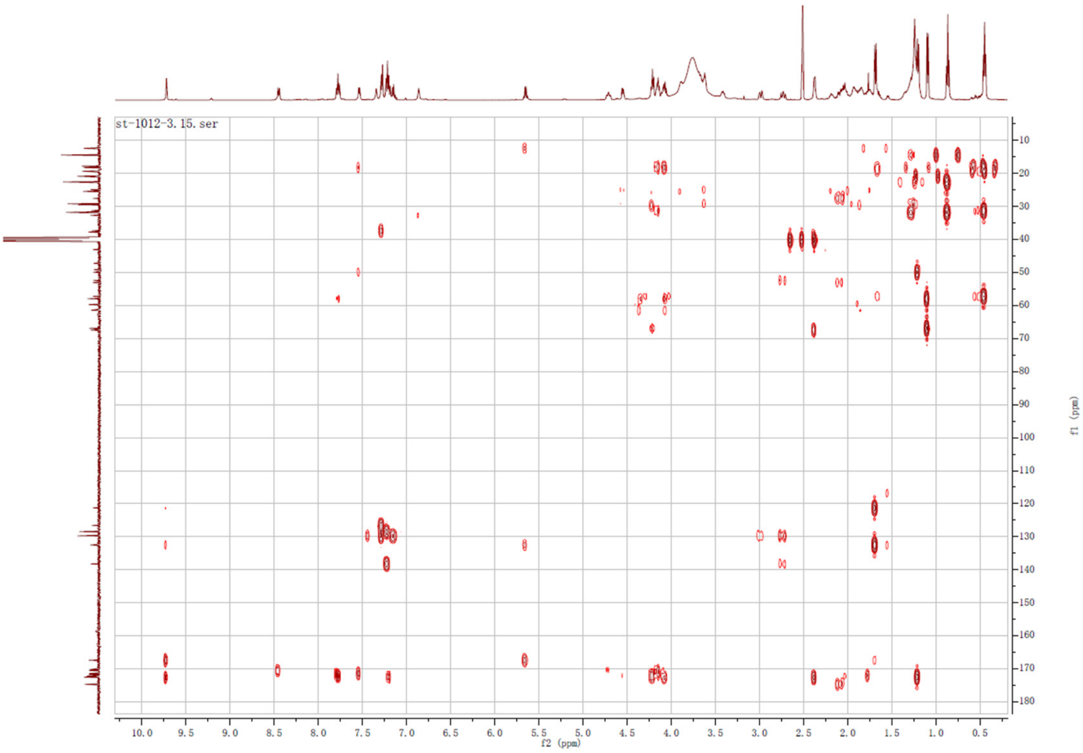

Figure S21. HMBC spectrum of burriogladiodin B (**2**) in DMSO-d_6

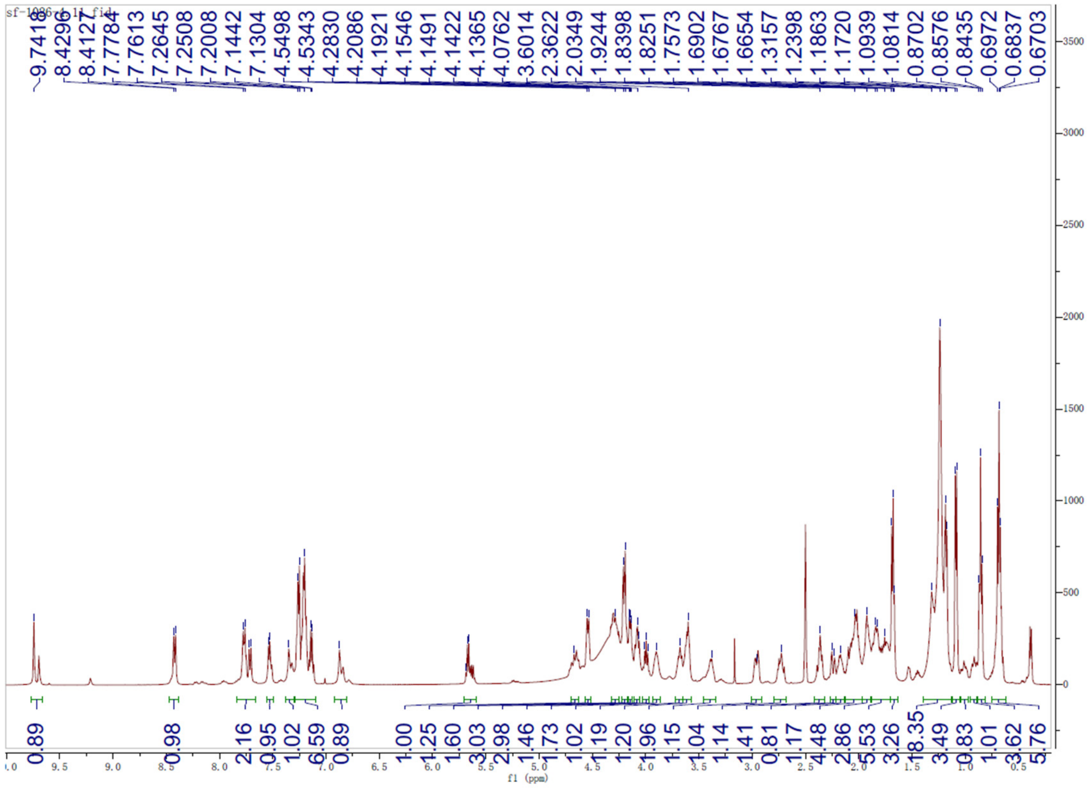

Figure S22. ^{1}H NMR spectrum of burriogladiodins C (**3**) and D (**4**) in DMSO-*d*6

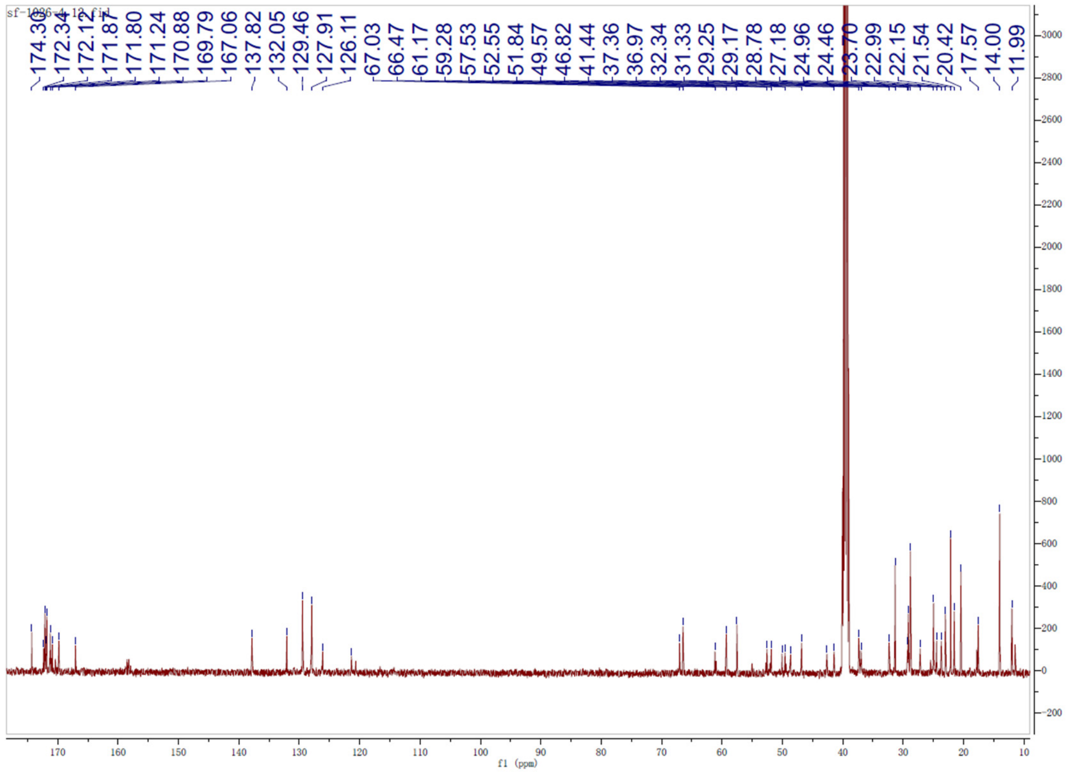

Figure S23. ^{13}C NMR spectrum of burriogladiodins C (**3**) and D (**4**) in DMSO-*d*6

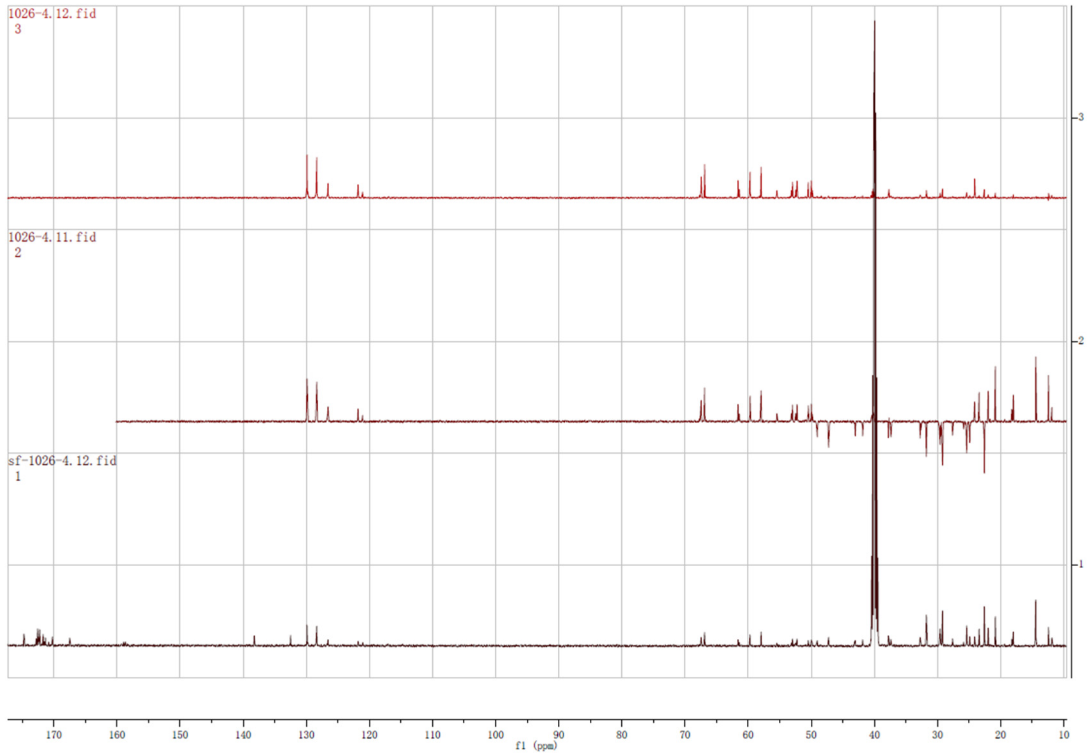

Figure S24. DEPT spectrum of burriogladiodins C (**3**) and D (**4**) in DMSO-d_6

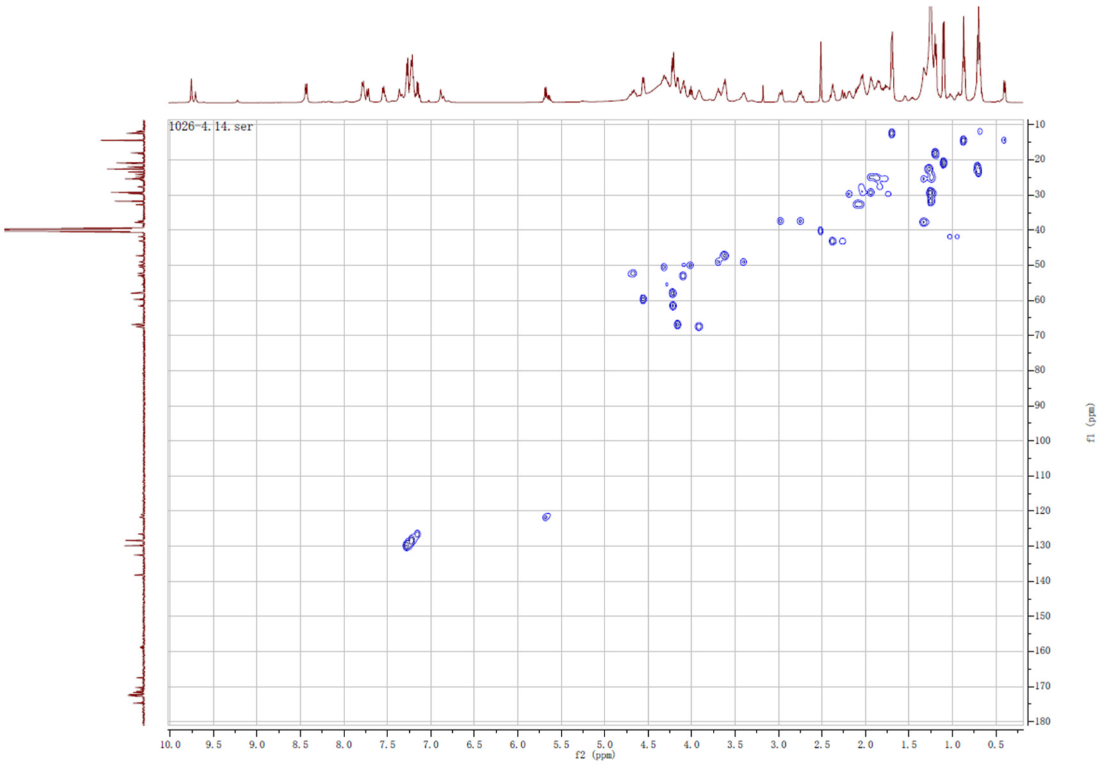

Figure S25. HSQC spectrum of burriogladiodins C (**3**) and D (**4**) in DMSO-d_6

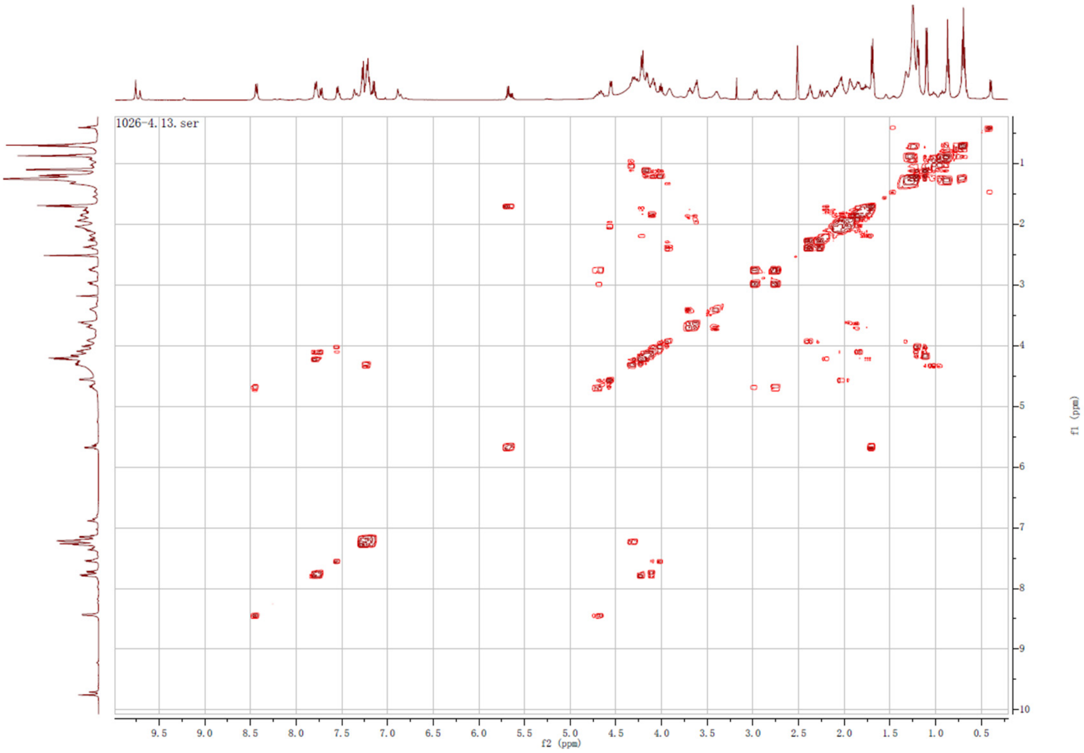

Figure S26. ^{1}H-^{1}H COSY spectrum of burriogladiodins C (**3**) and D (**4**) in DMSO-d_6

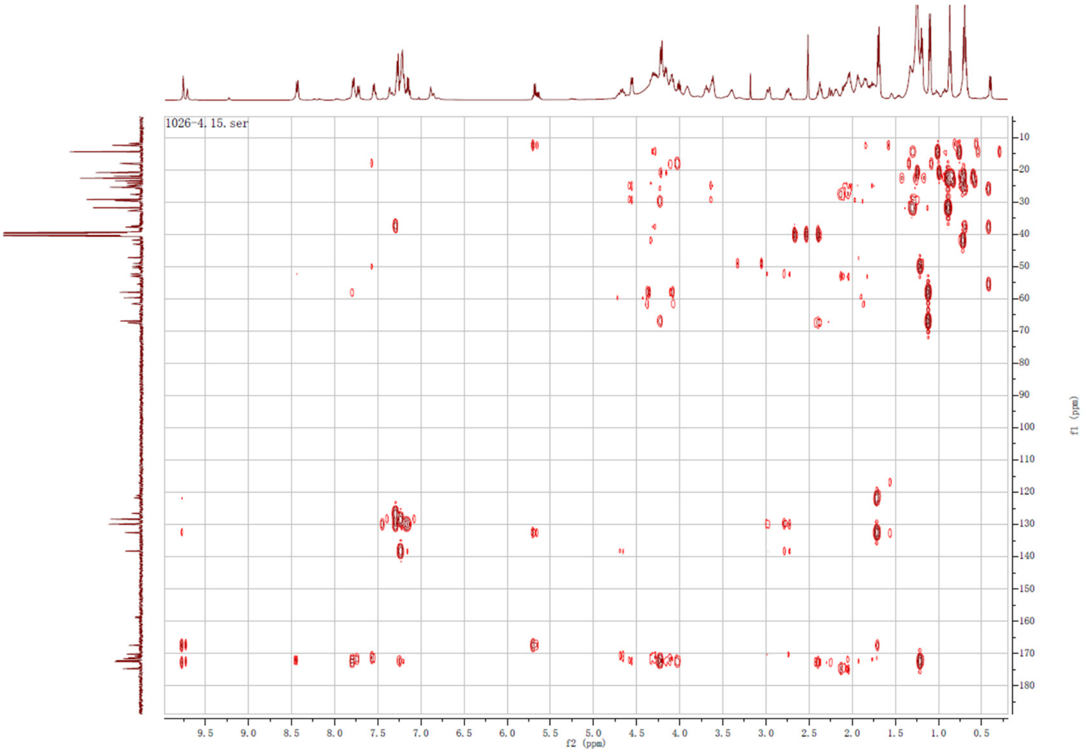

Figure S27. HMBC spectrum of burriogladiodins C (**3**) and D (**4**) in DMSO-d_6

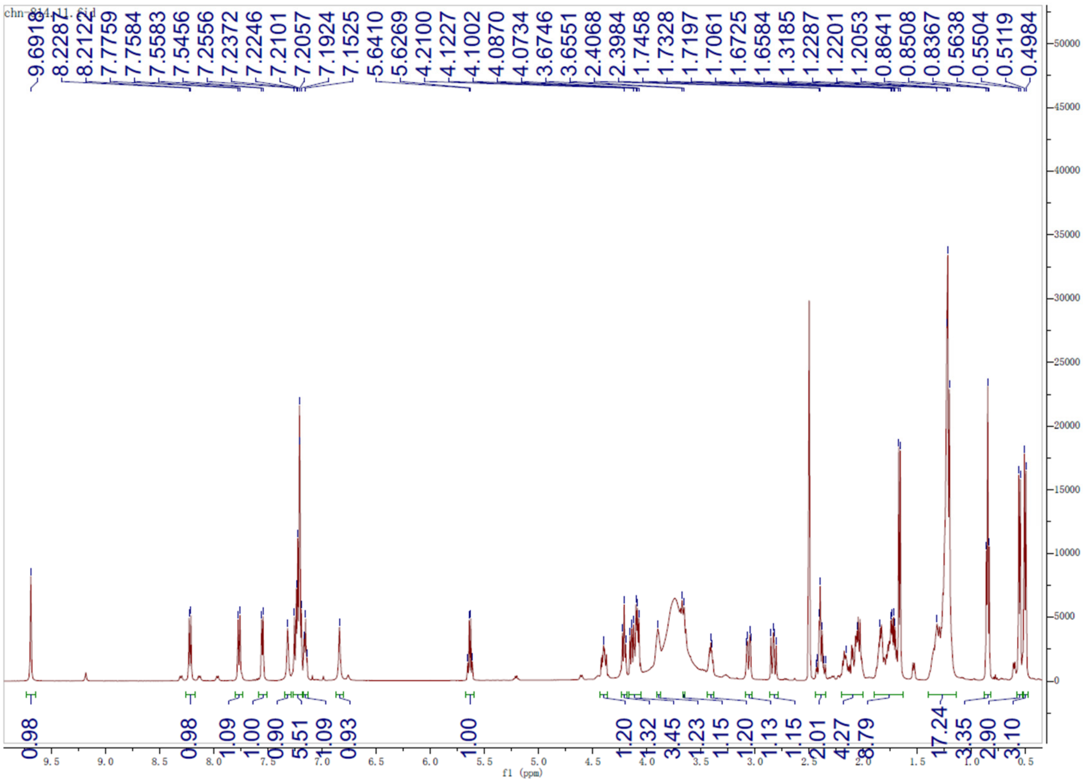

Figure S28. ^{1}H NMR spectrum of burriogladiodin E (**5**) in DMSO-d_6

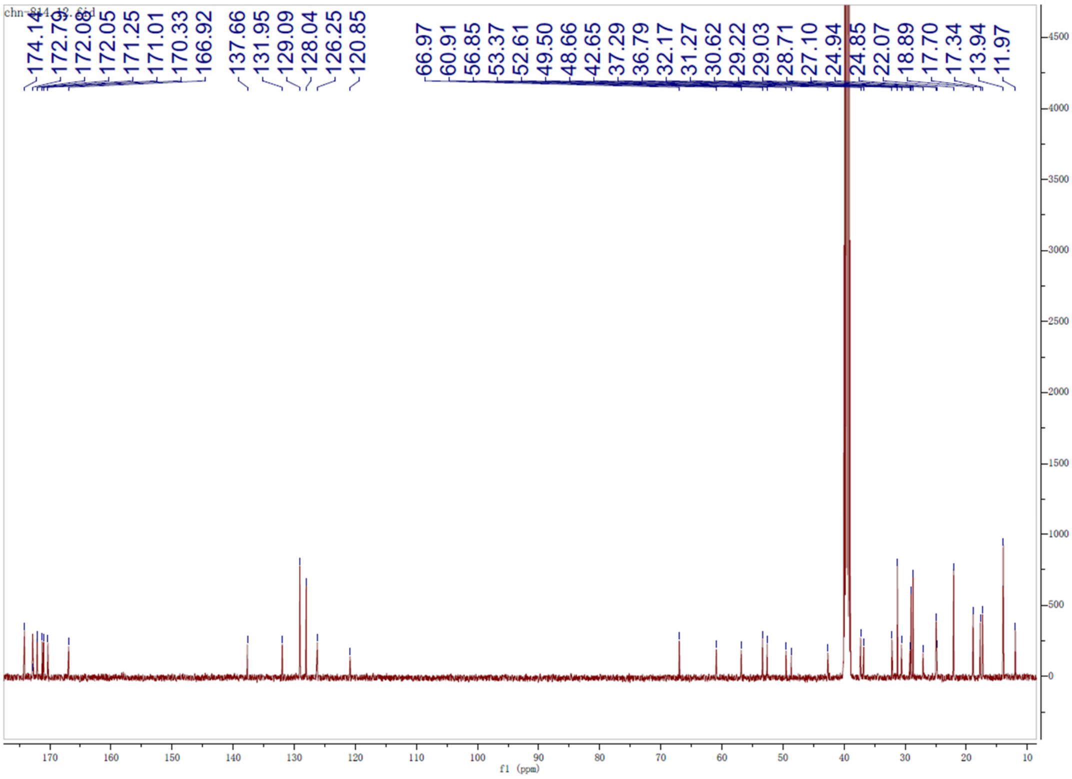

Figure S29. ^{13}C NMR spectrum of burriogladiodin E (**5**) in DMSO-d_6

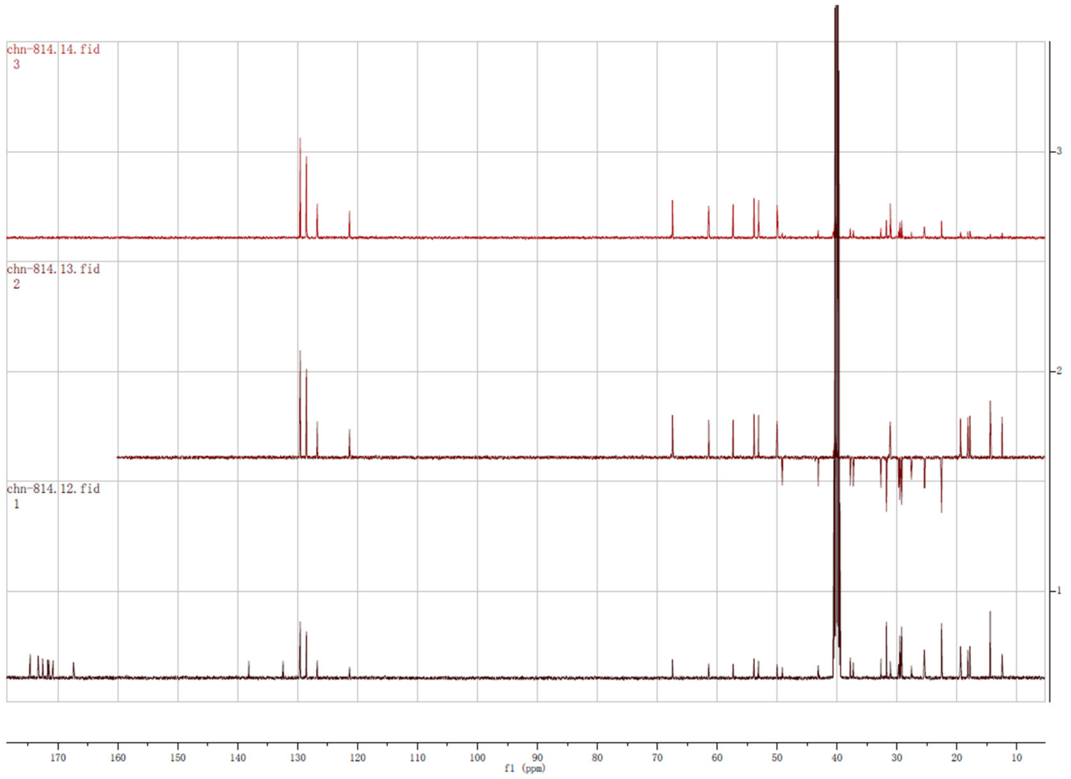

Figure S30. DEPT spectrum of burriogladiodin E (**5**) in DMSO-d_6

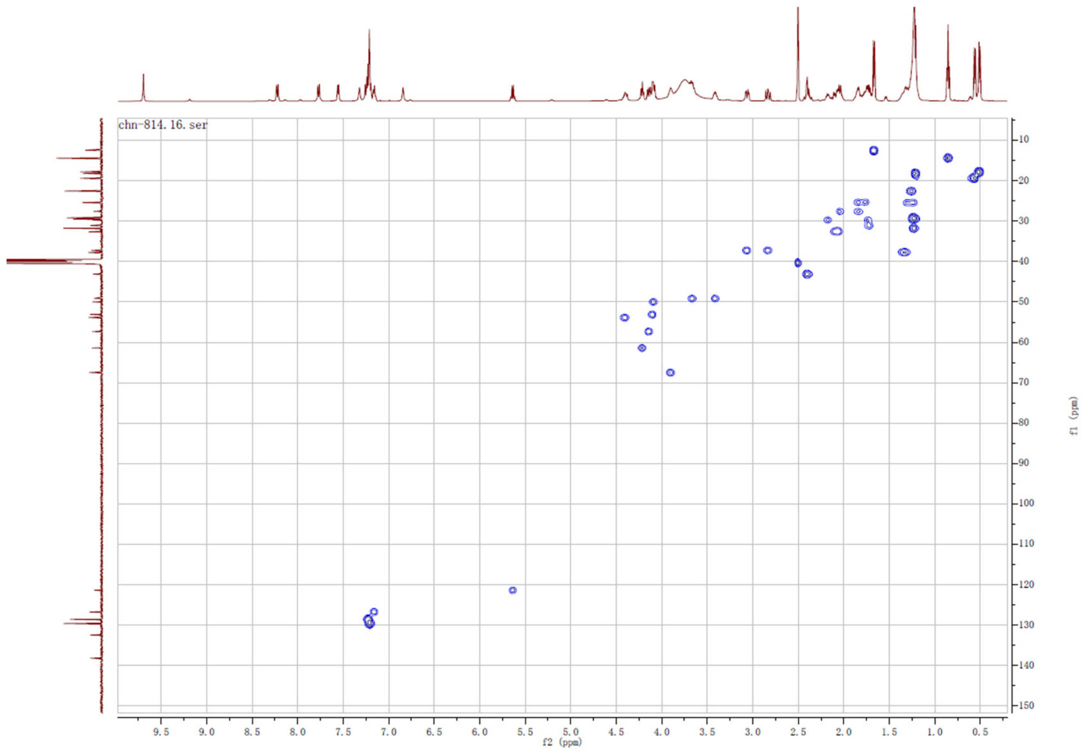

Figure S31. HSQC spectrum of burriogladiodin E (**5**) in DMSO-d_6

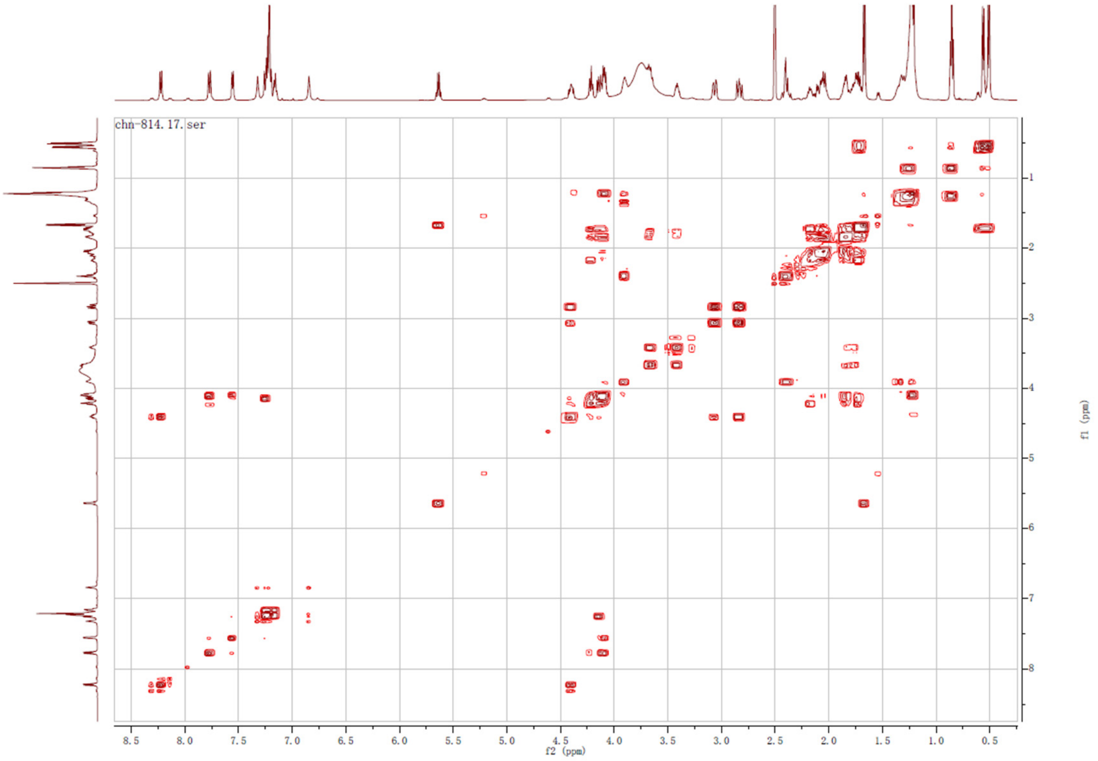

Figure S32. ^{1}H-^{1}H COSY spectrum of burriogladiodin E (**5**) in DMSO-d_6

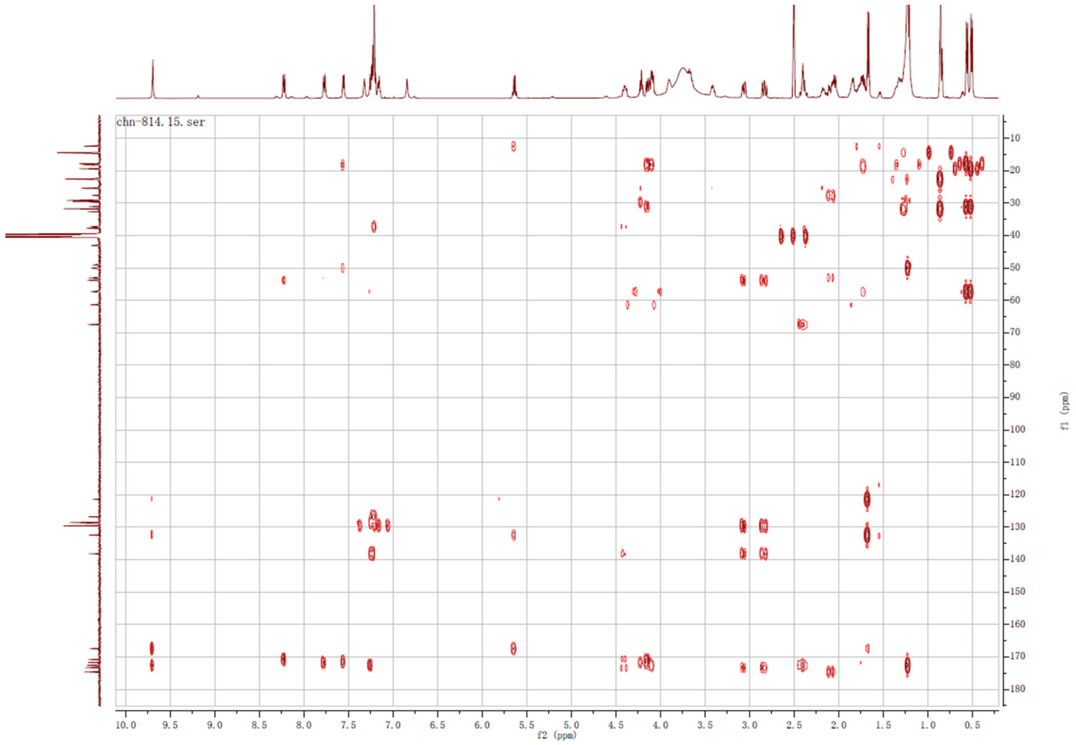

Figure S33. HMBC spectrum of burriogladiodin E (**5**) in DMSO-d_6

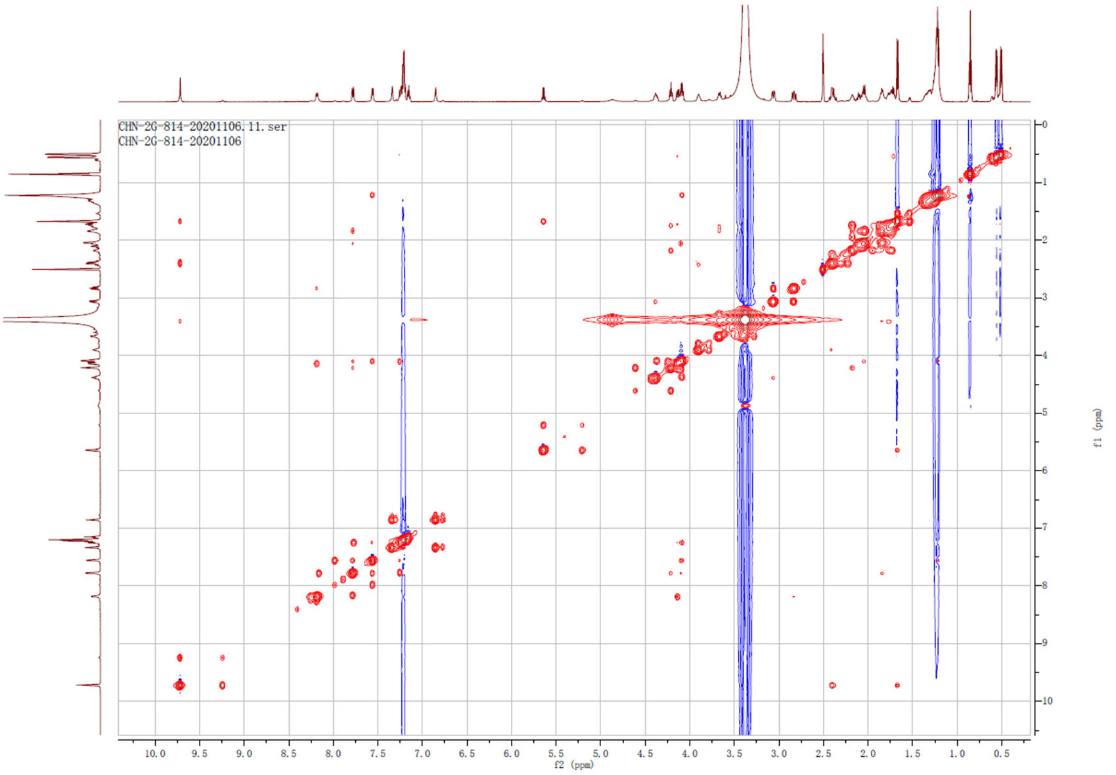

Figure S34. NOESY spectrum of burriogladiodin E (**5**) in DMSO-d_6

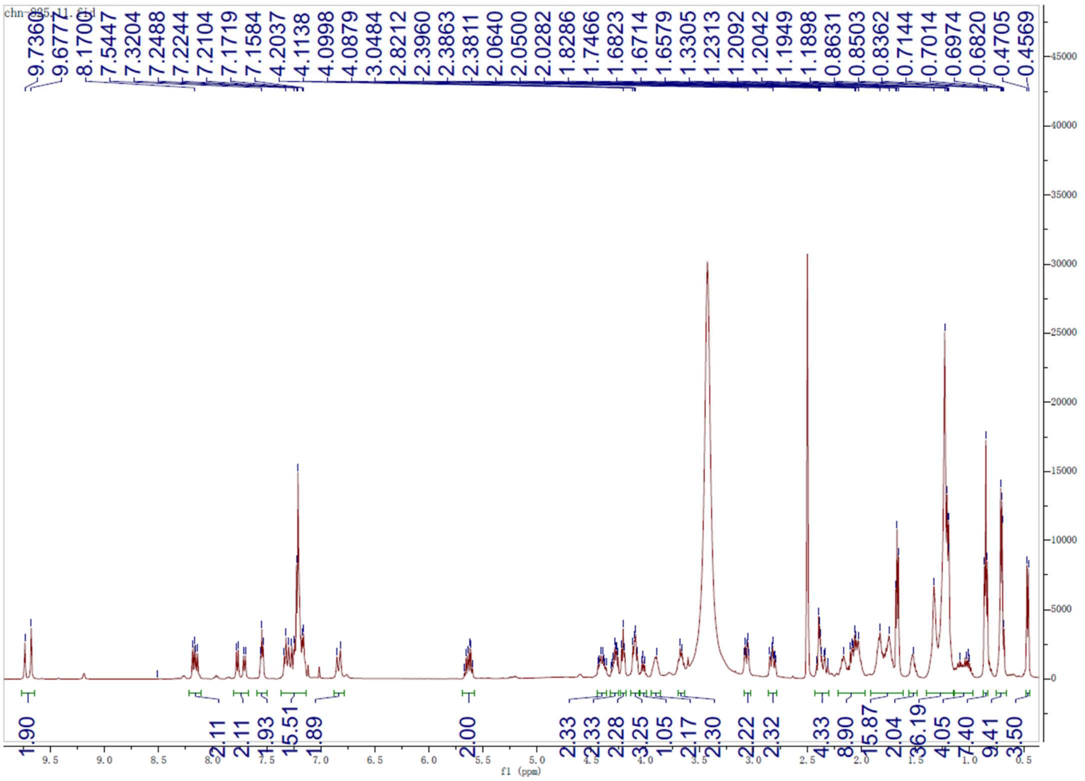

Figure S35. ^{1}H NMR spectrum of burriogladiodins F (**6**) and G (**7**) in DMSO-d_6

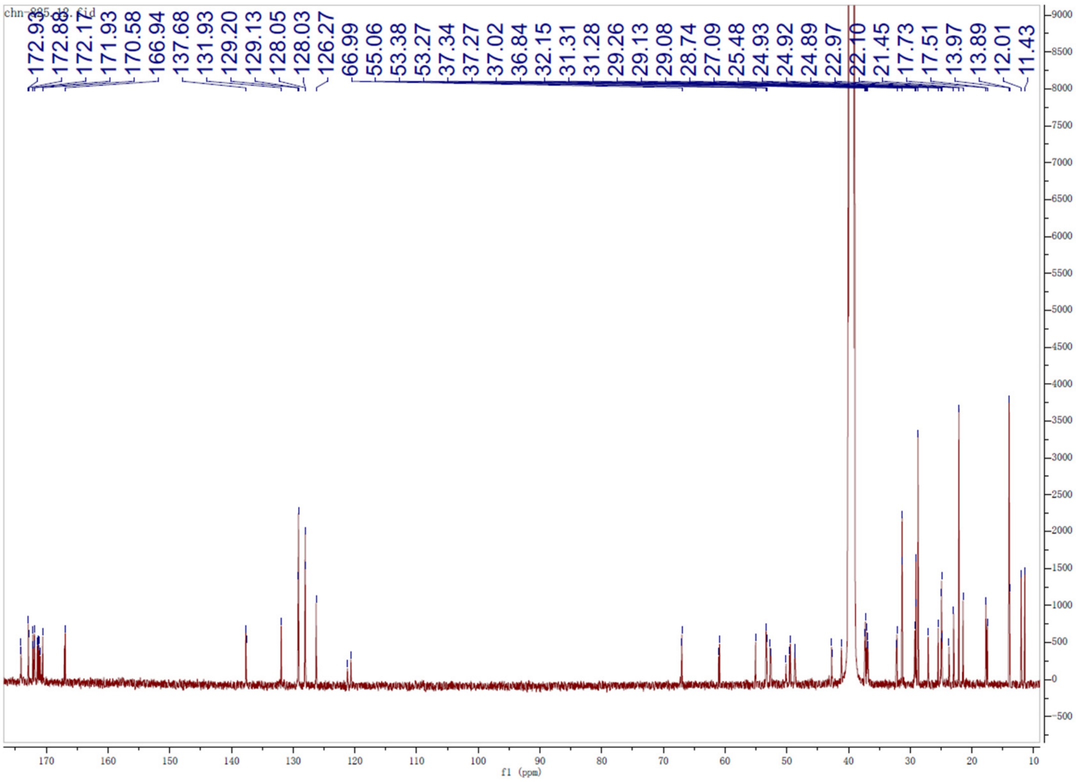

Figure S36. ^{13}C NMR spectrum of burriogladiodins F (**6**) and G (**7**) in DMSO-d_6

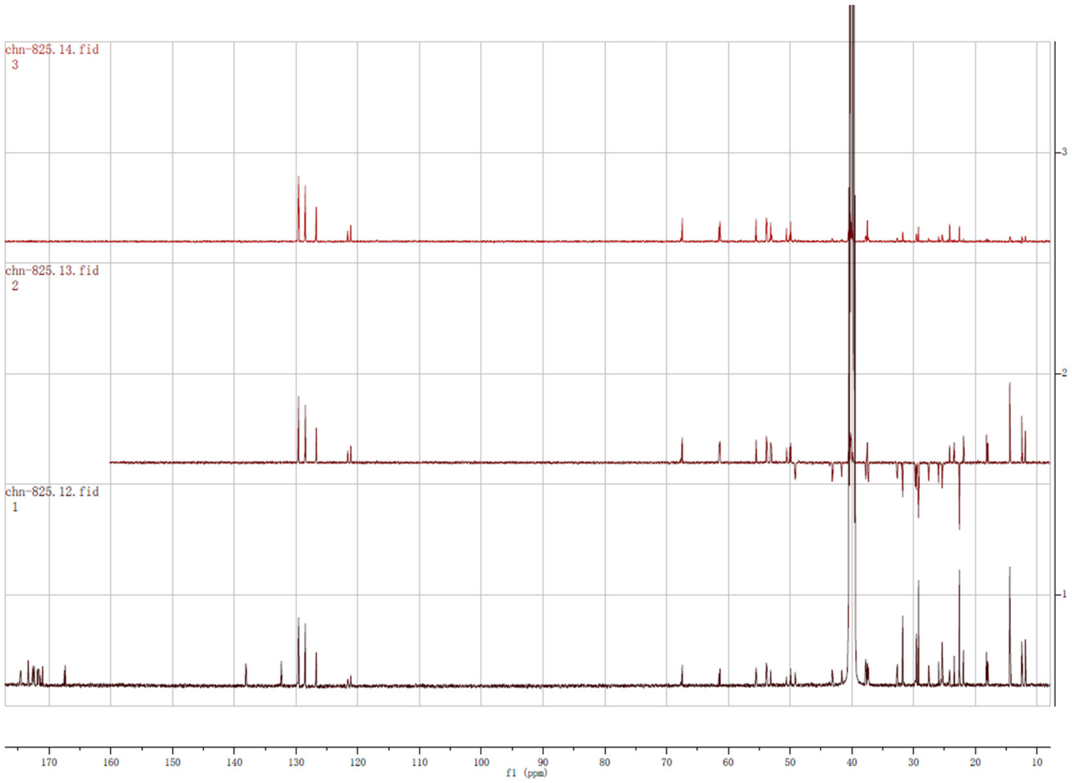

Figure S37. DEPT spectrum of burriogladiodins F (**6**) and G (**7**) in DMSO-d_6

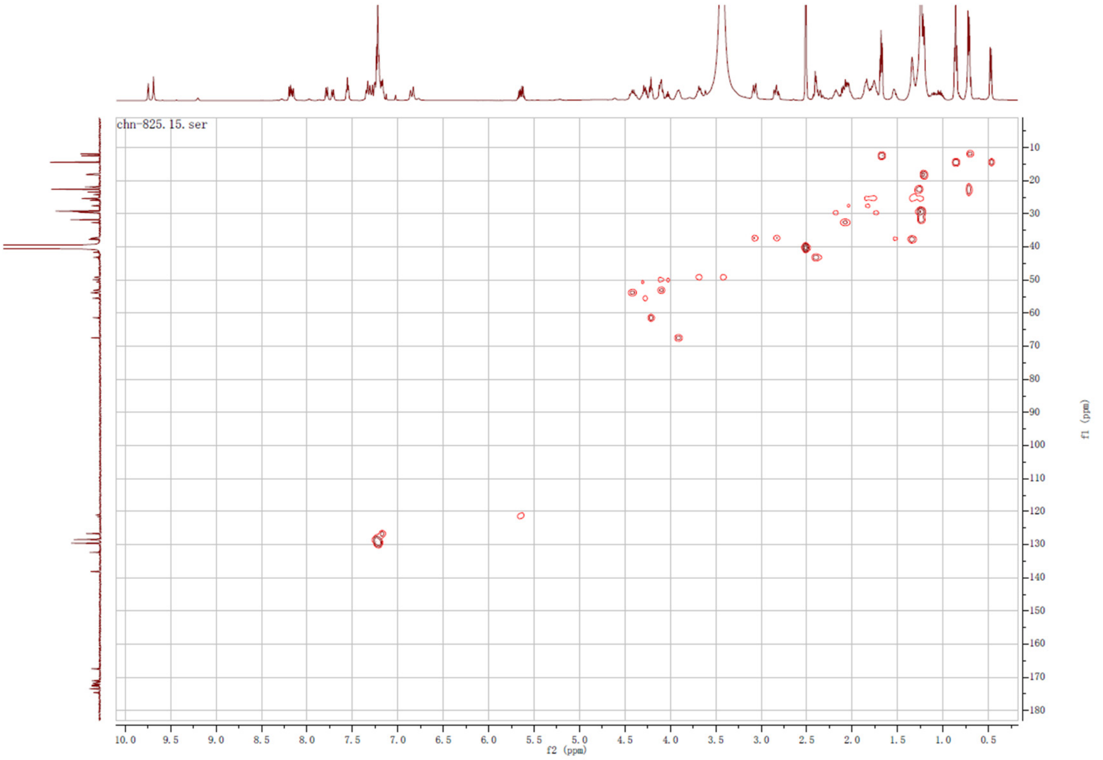

Figure S38. HSQC spectrum of burriogladiodins F (**6**) and G (**7**) in DMSO-d_6

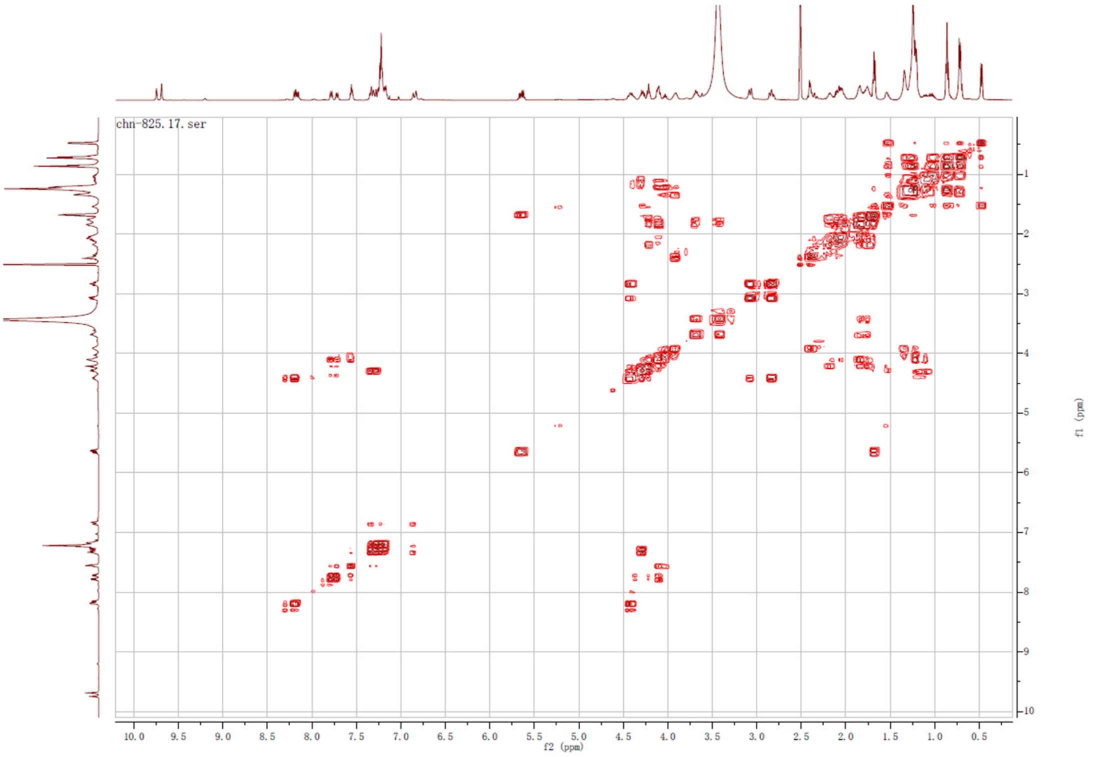

Figure S39. ^{1}H-^{1}H COSY spectrum of burriogladiodins F (**6**) and G (**7**) in DMSO-d_6

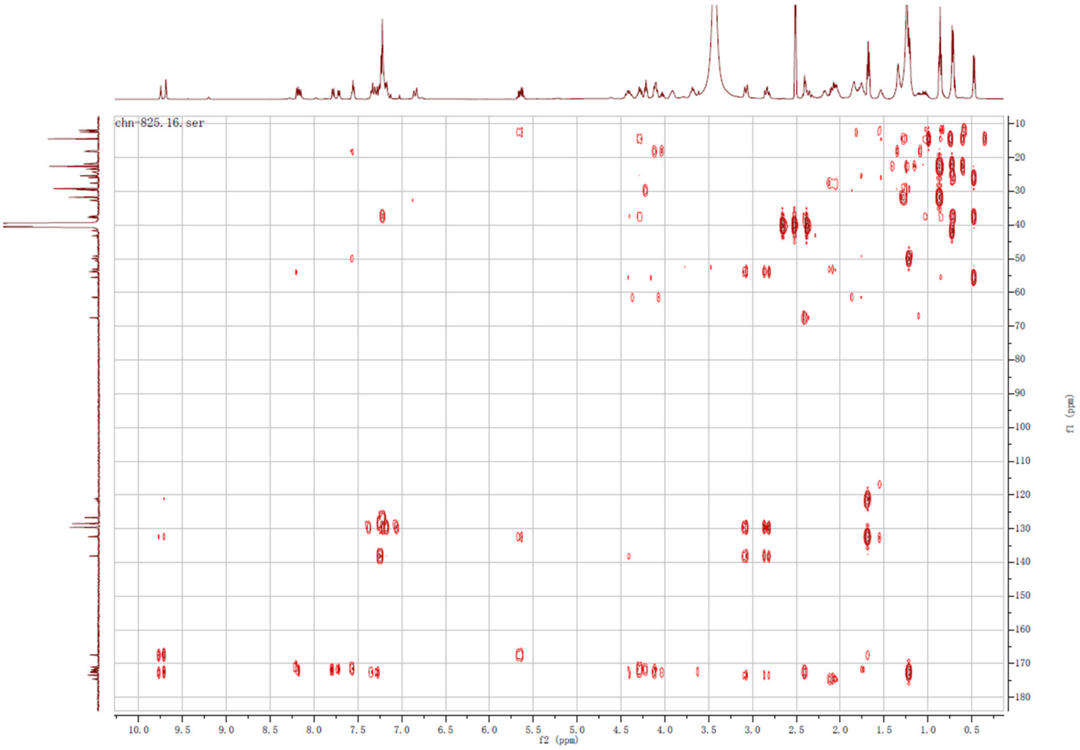

Figure S40. HMBC spectrum of burriogladiodins F (**6**) and G (**7**) in DMSO-*d*$_6$

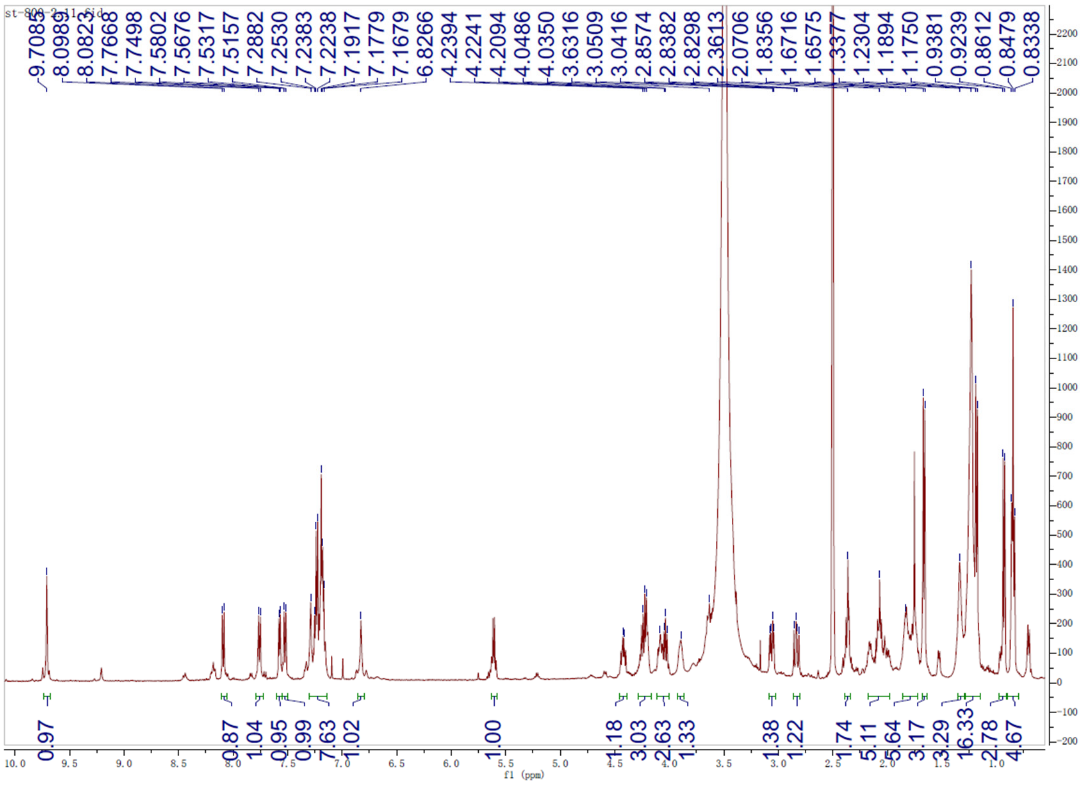

Figure S41. ^{1}H NMR spectrum of burriogladiodin H (**8**) in DMSO-*d*$_6$

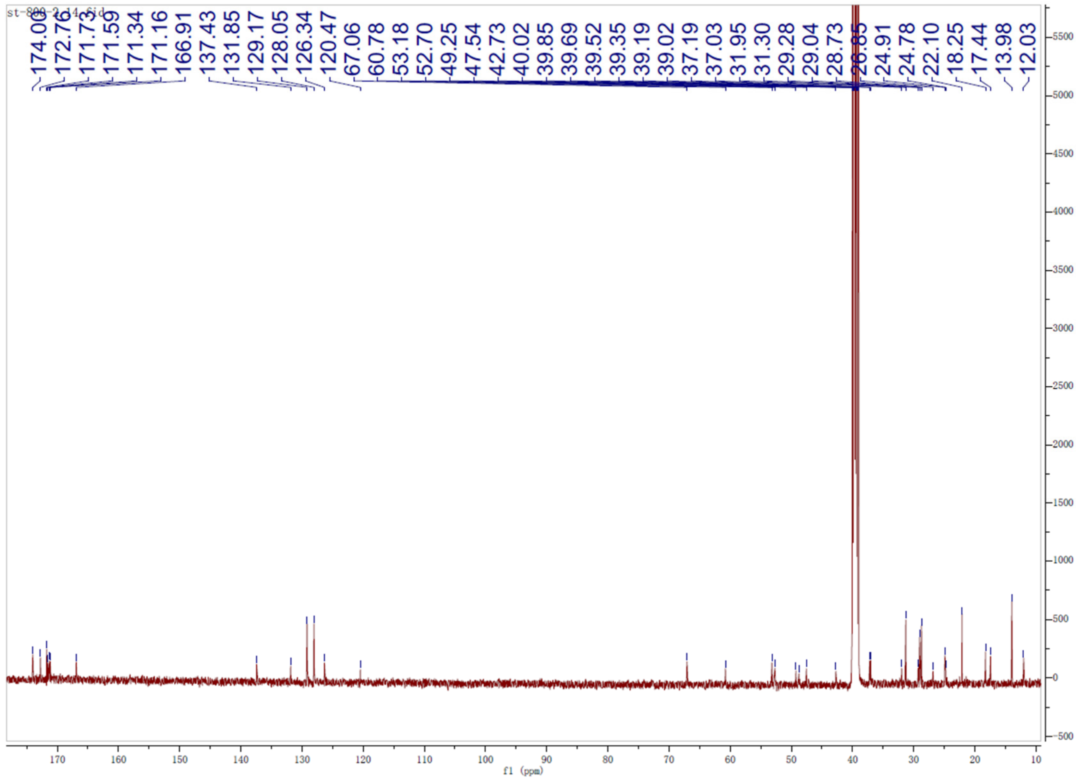

Figure S42. ^{13}C NMR spectrum of burriogladiodin H (**8**) in DMSO-d_6

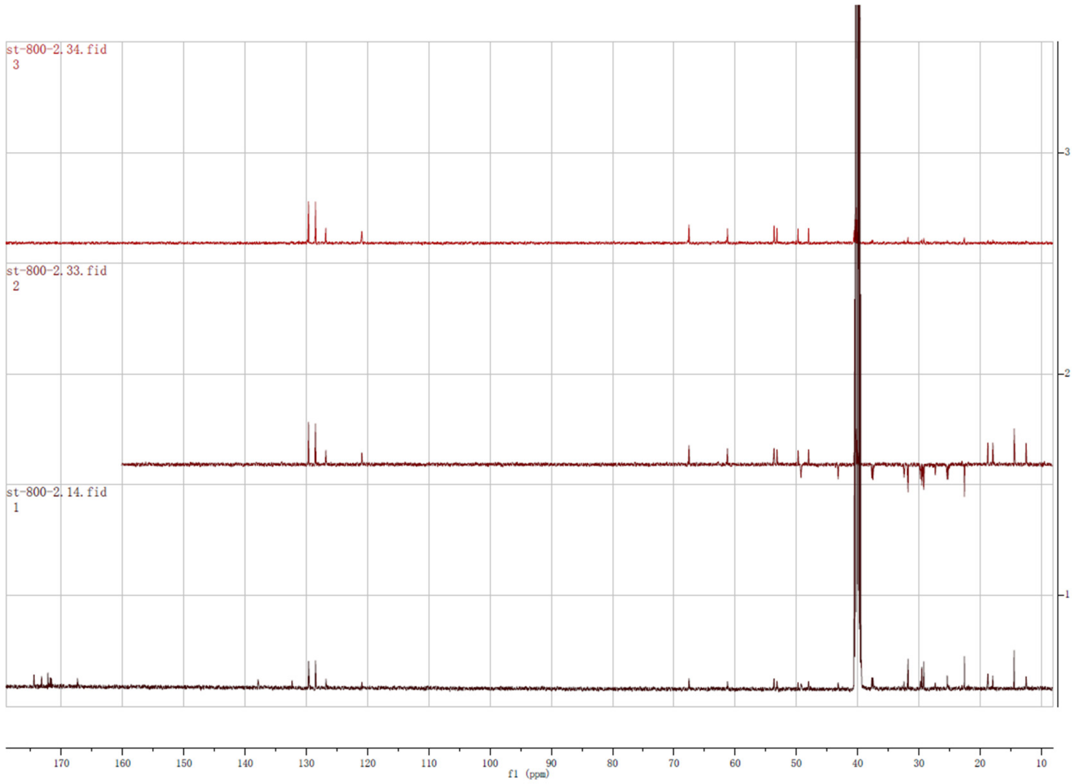

Figure S43. DEPT spectrum of burriogladiodin H (**8**) in DMSO-d_6

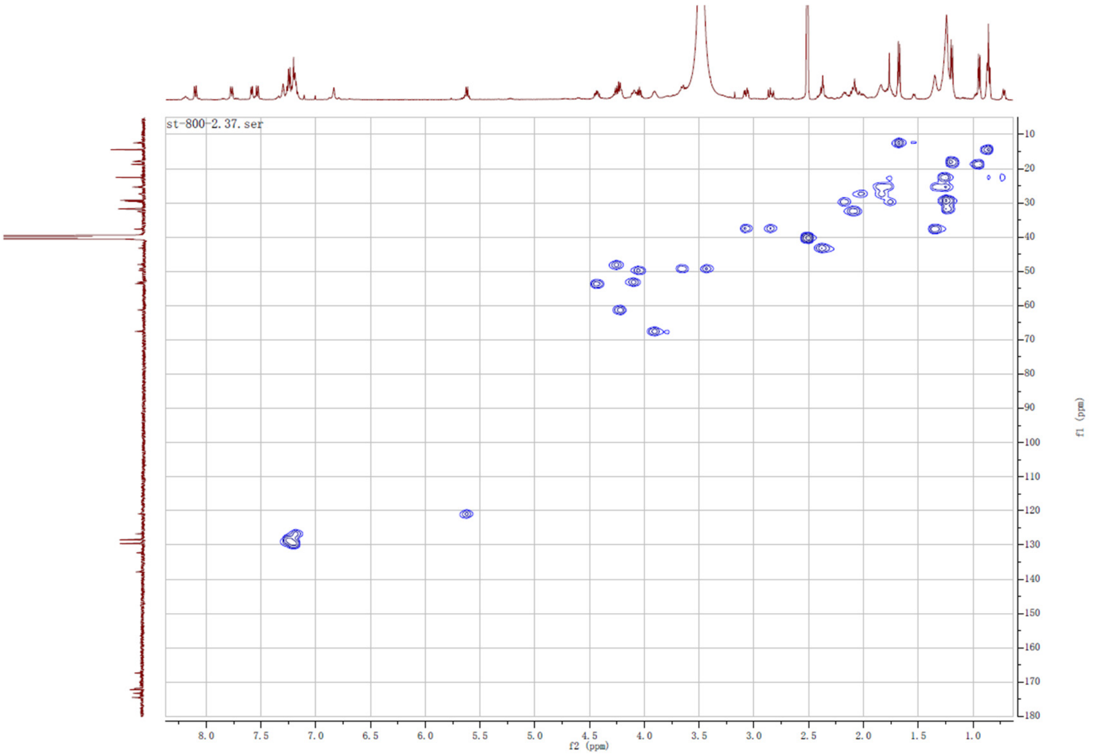

Figure S44. HSQC spectrum of burriogladiodin H (**8**) in DMSO-*d*6

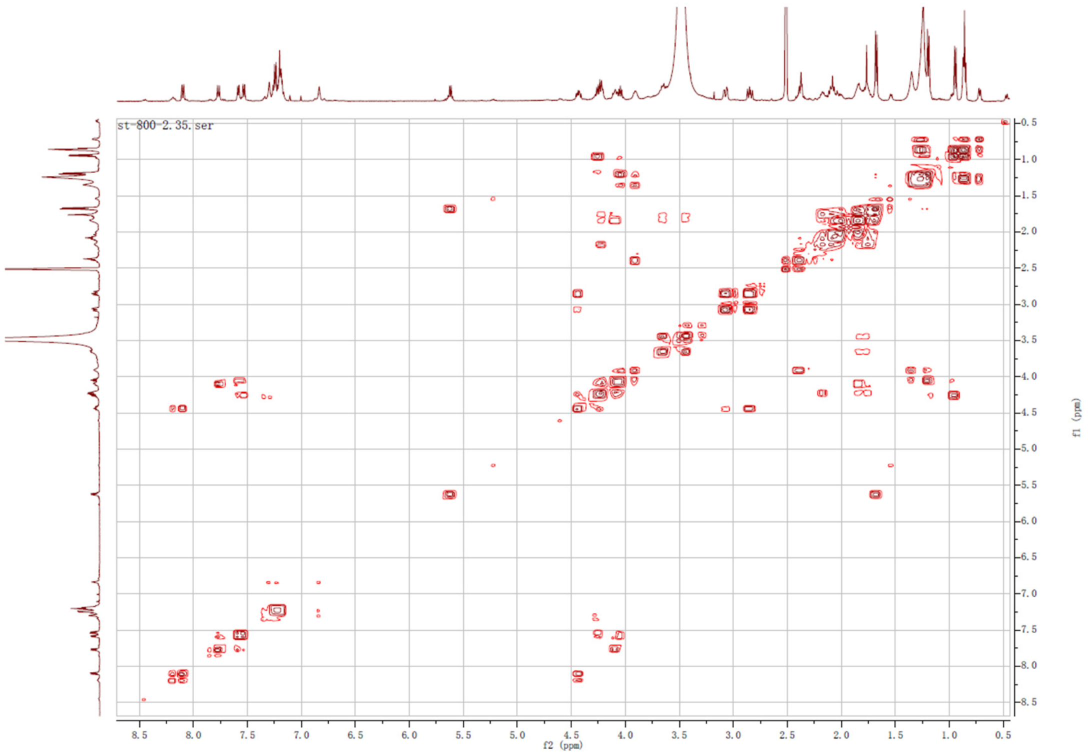

Figure S45. ^{1}H-^{1}H COSY spectrum of burriogladiodin H (**8**) in DMSO-*d*6

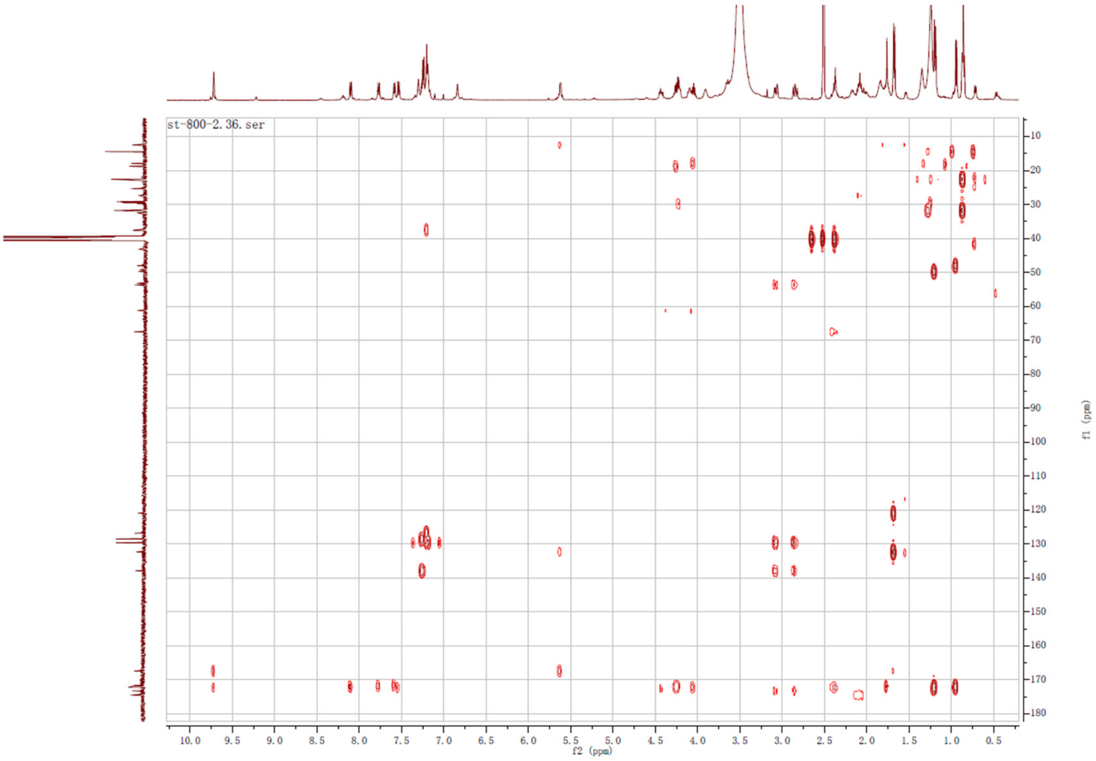

Figure S46. HMBC spectrum of burriogladiodin H (**8**) in DMSO-*d*₆

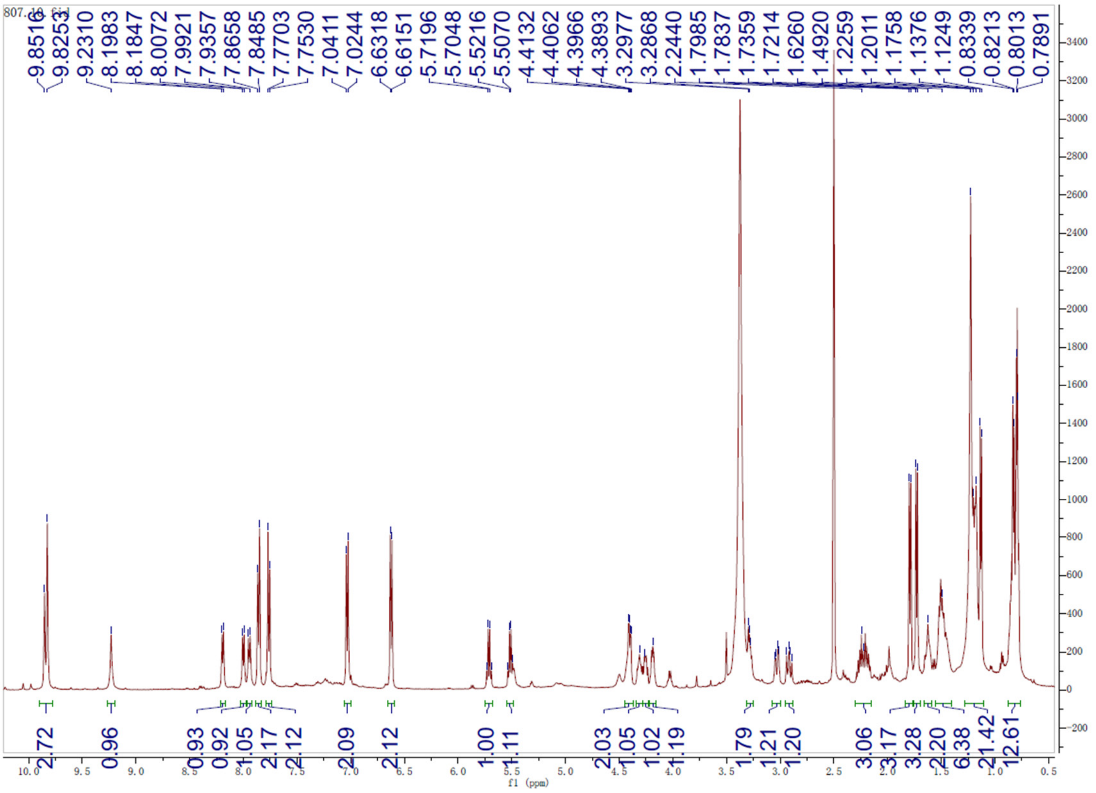

Figure S47. ¹H NMR spectrum of haereogladiodin A (**9**) in DMSO-*d*₆

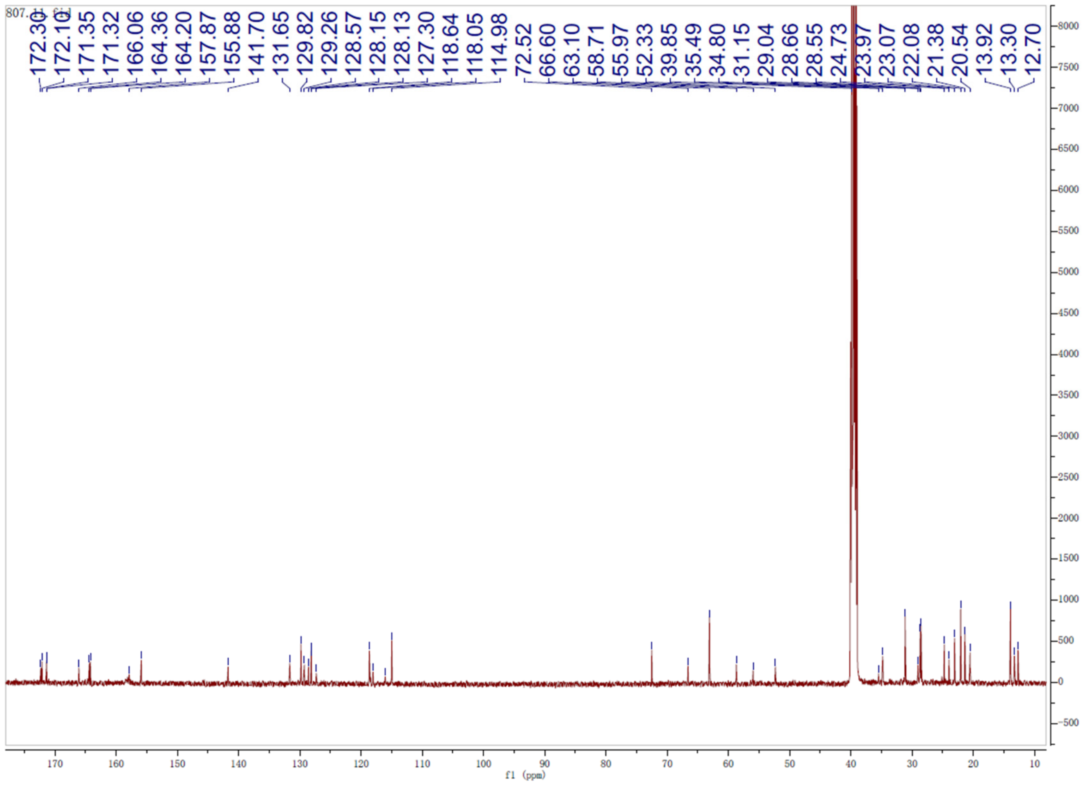

Figure S48. ^{13}C NMR spectrum of haereogladiodin A (**9**) in DMSO-d_6

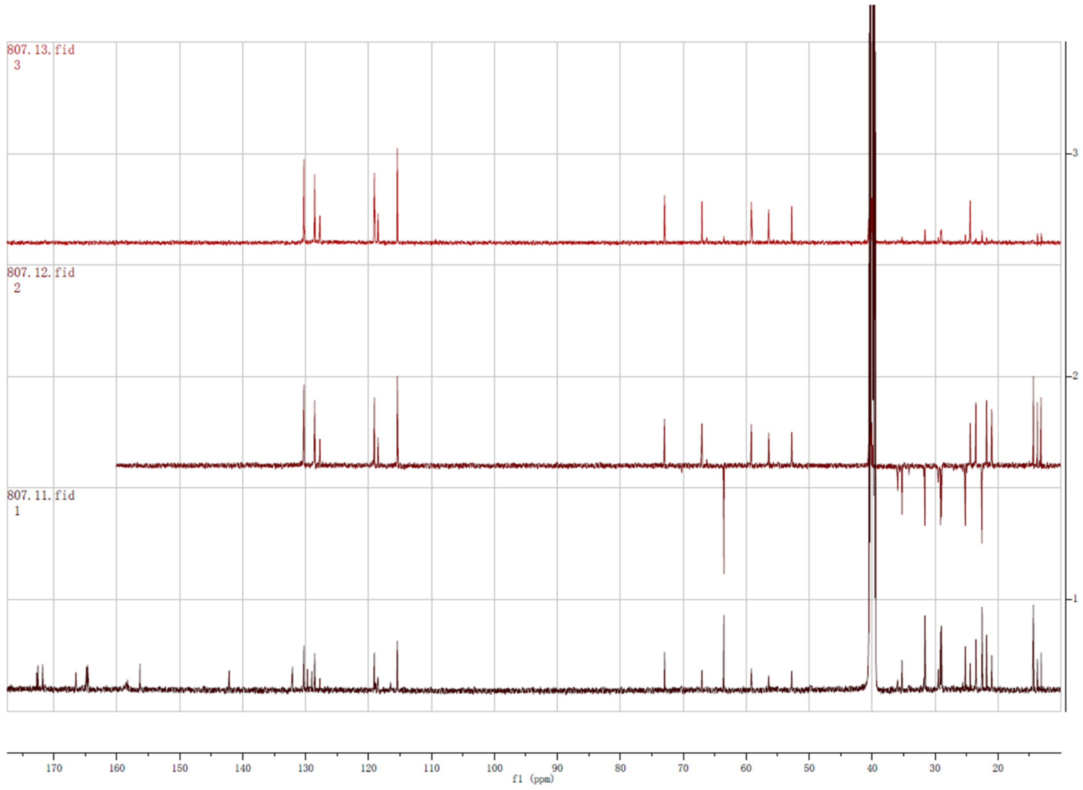

Figure S49. DEPT spectrum of haereogladiodin A (**9**) in DMSO-*d*6

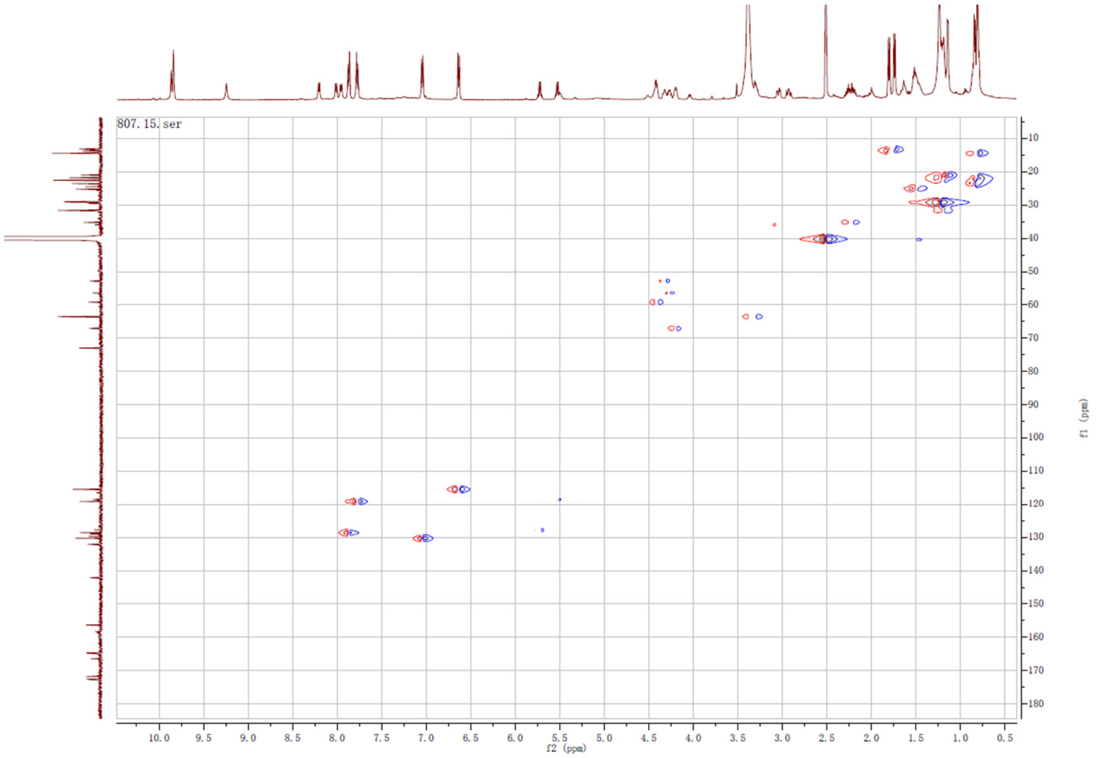

Figure S50. HSQC spectrum of haereogladiodin A (**9**) in DMSO-*d*6

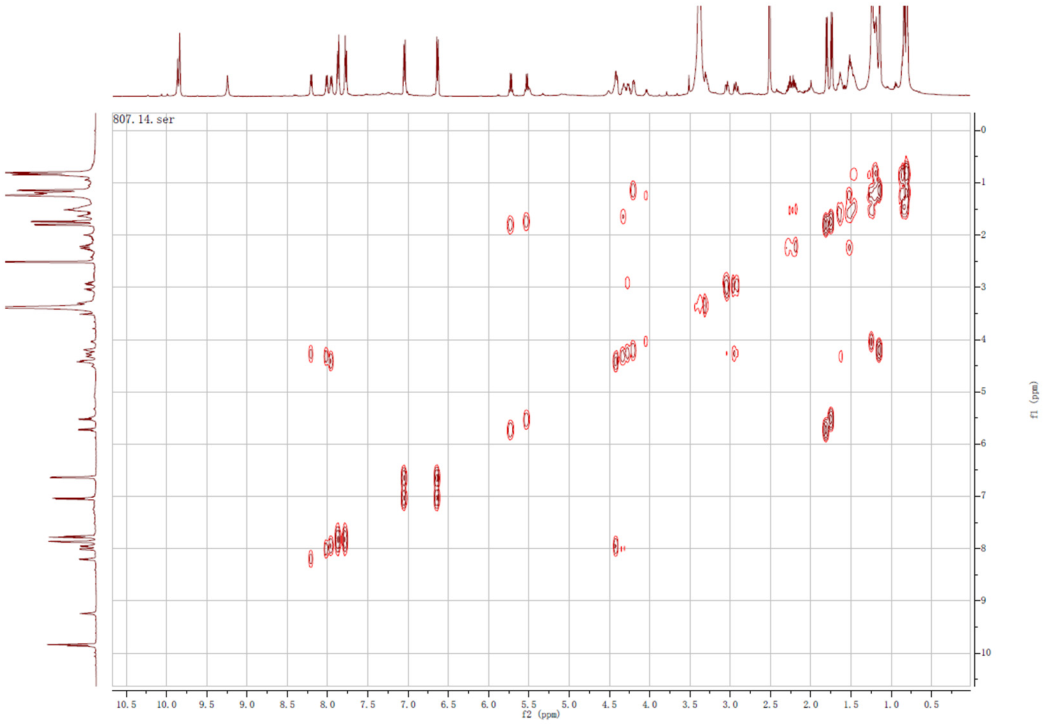

Figure S51. ^{1}H-^{1}H COSY spectrum of haereogladiodin A (**9**) in DMSO-*d*6

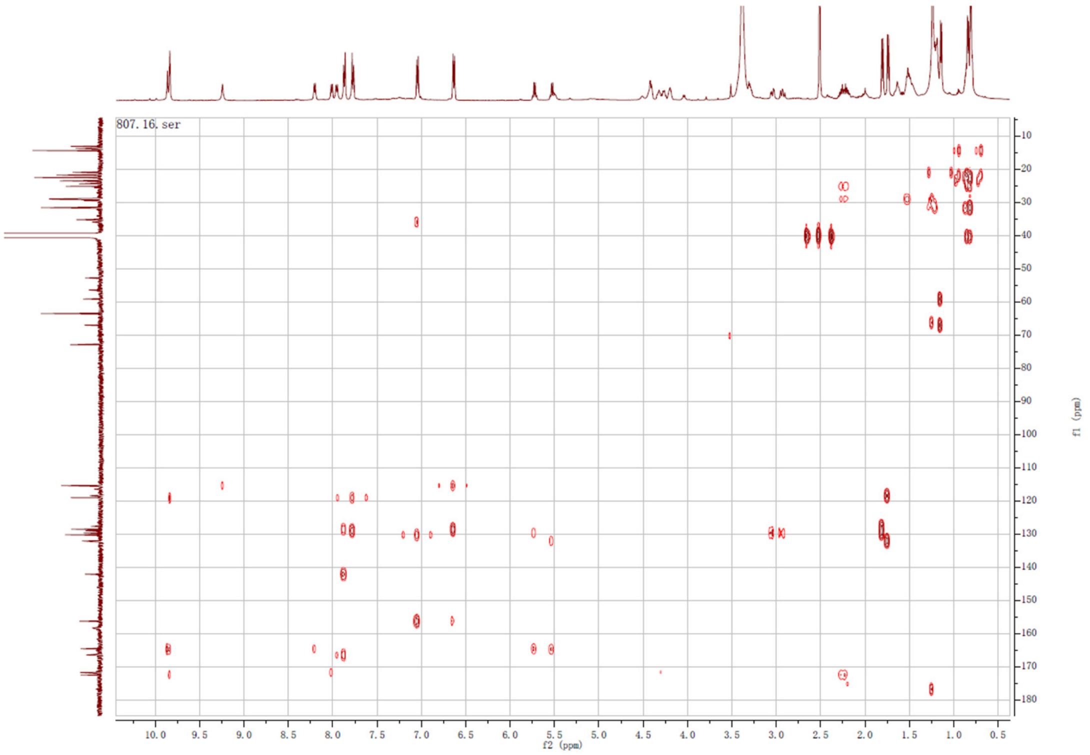

Figure S52. HMBC spectrum of haereogladiodin A (**9**) in DMSO-*d*6

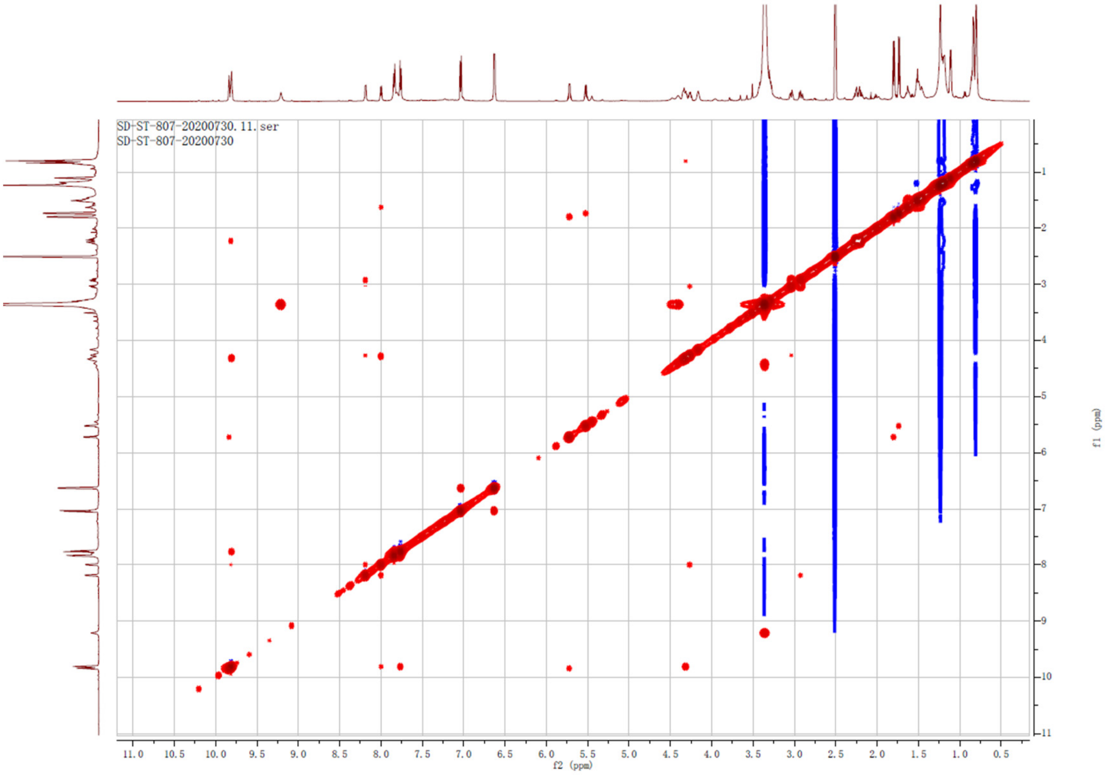

Figure S53. NOESY spectrum of haereogladiodin A (**9**) in DMSO-*d*6

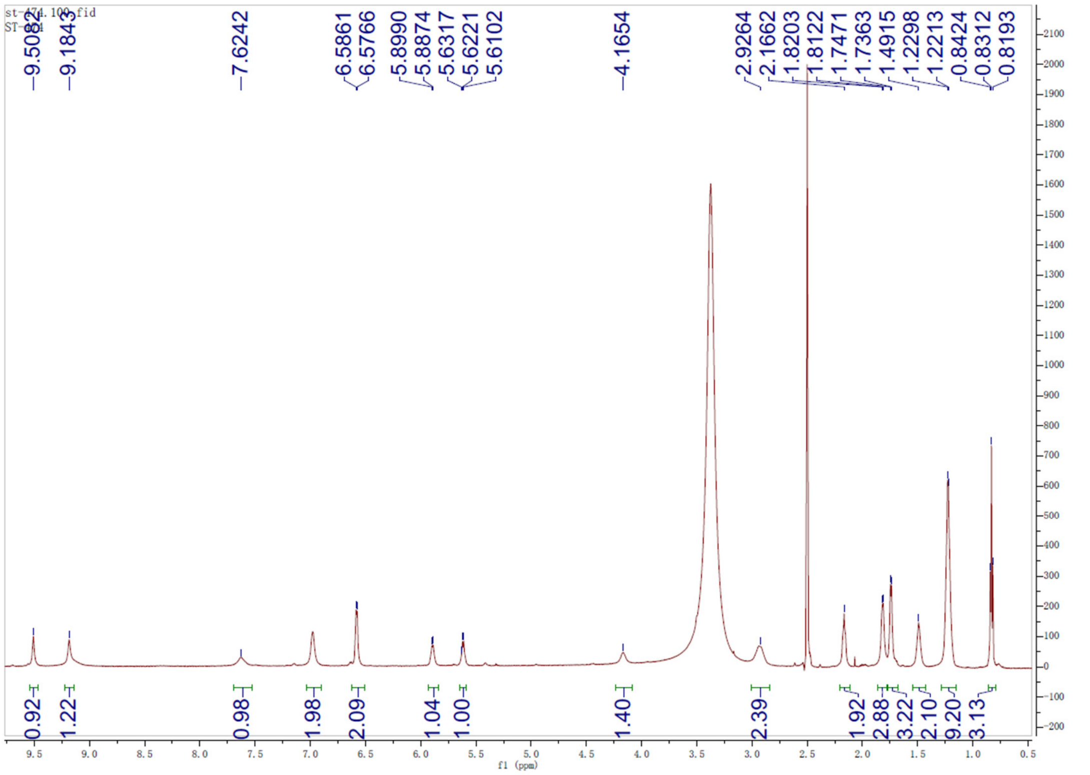

Figure S54. ^{1}H NMR spectrum of haereogladiodin B (**10**) in DMSO-d_6

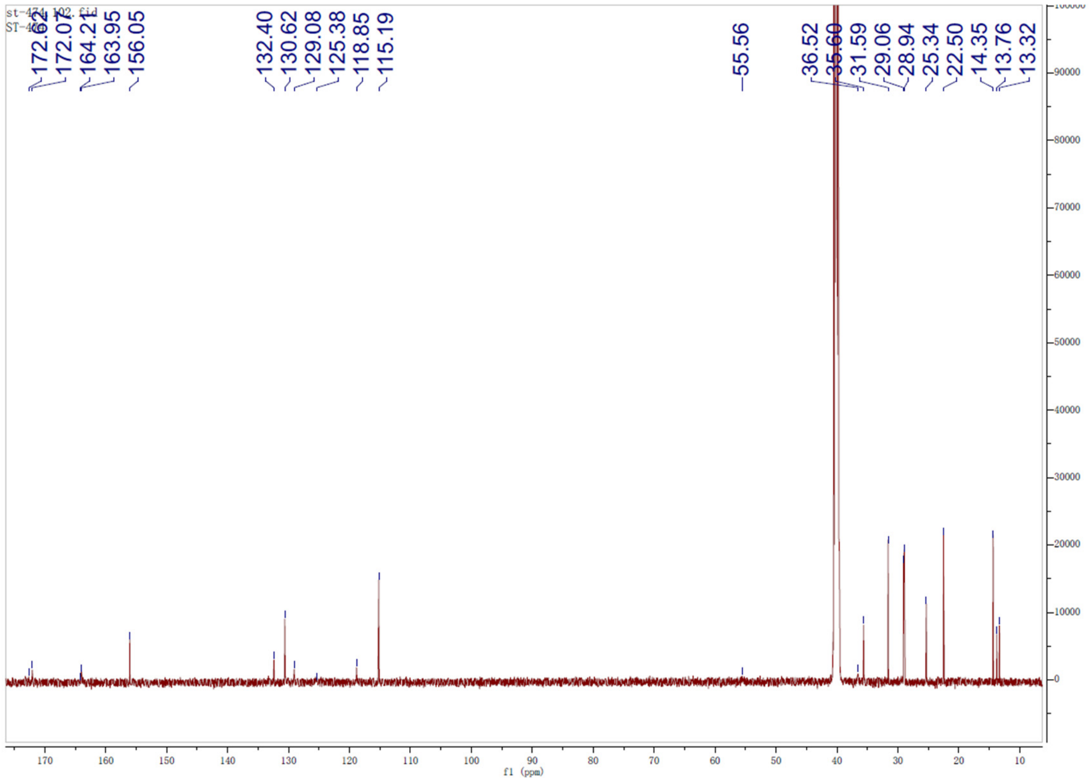

Figure S55. ^{13}C NMR spectrum of haereogladiodin B (**10**) in DMSO-d_6

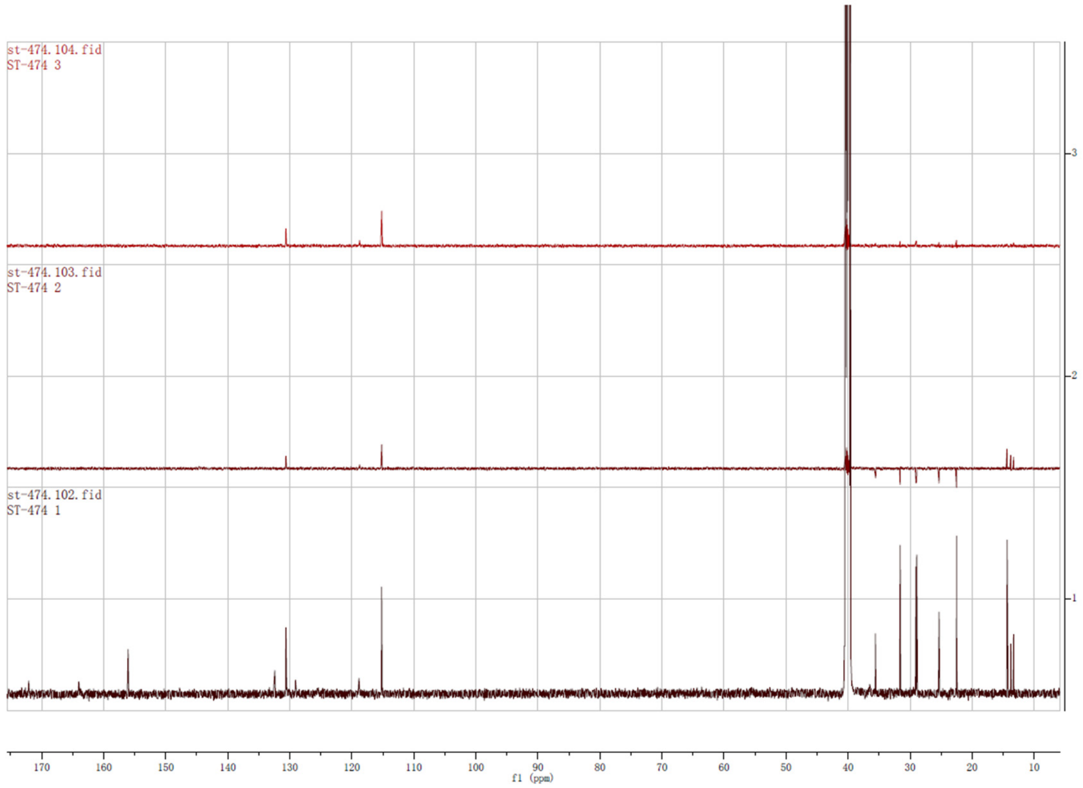

Figure S56. DEPT spectrum of haereogladiodin B (**10**) in DMSO-d_6

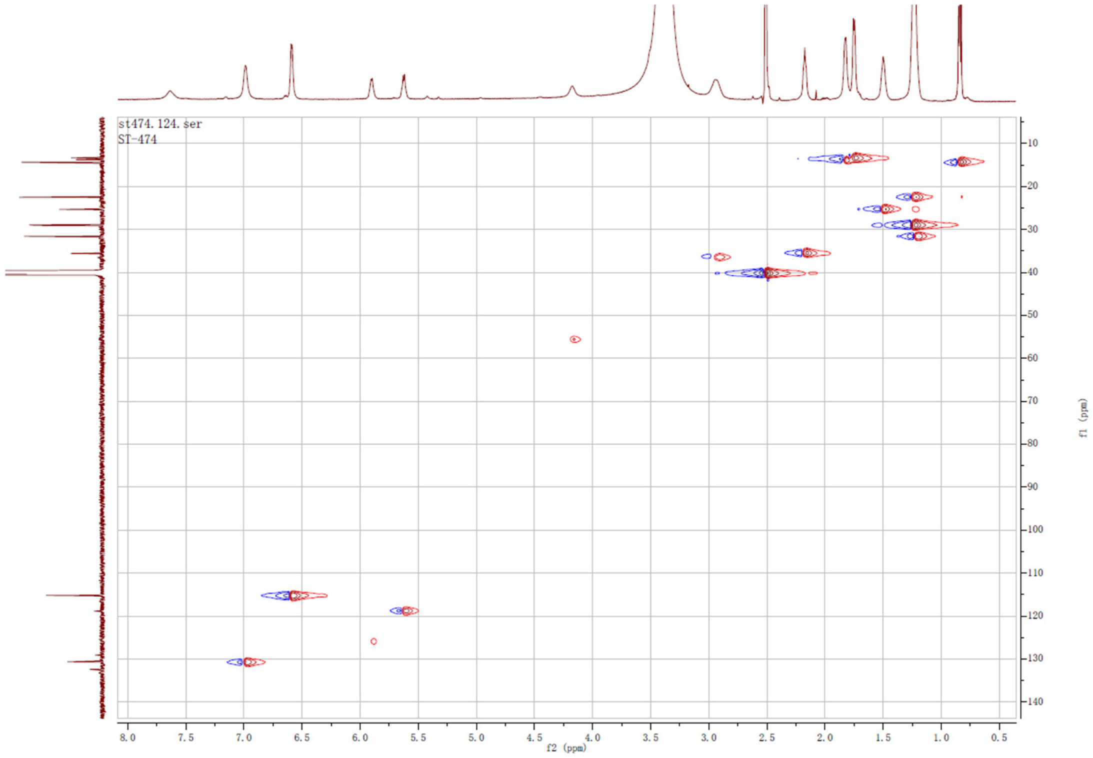

Figure S57. HSQC spectrum of haereogladiodin B (**10**) in DMSO-d_6

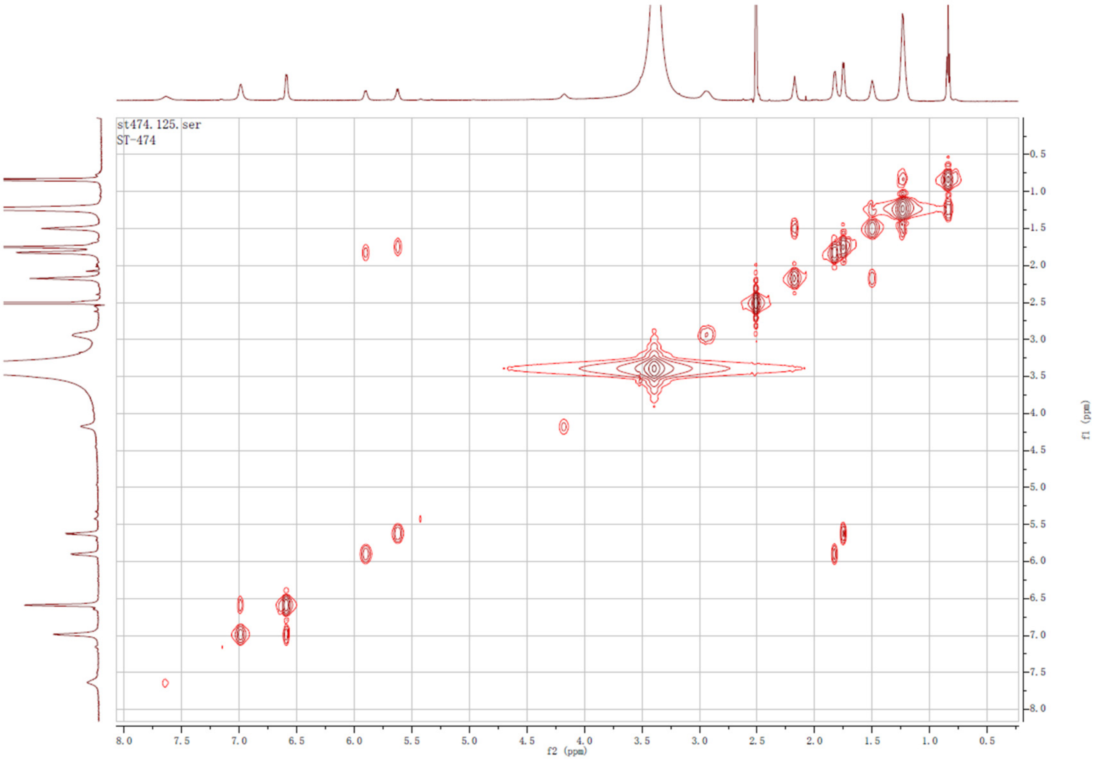

Figure S58. ^{1}H-^{1}H COSY spectrum of haereogladiodin B (**10**) in DMSO-d_6

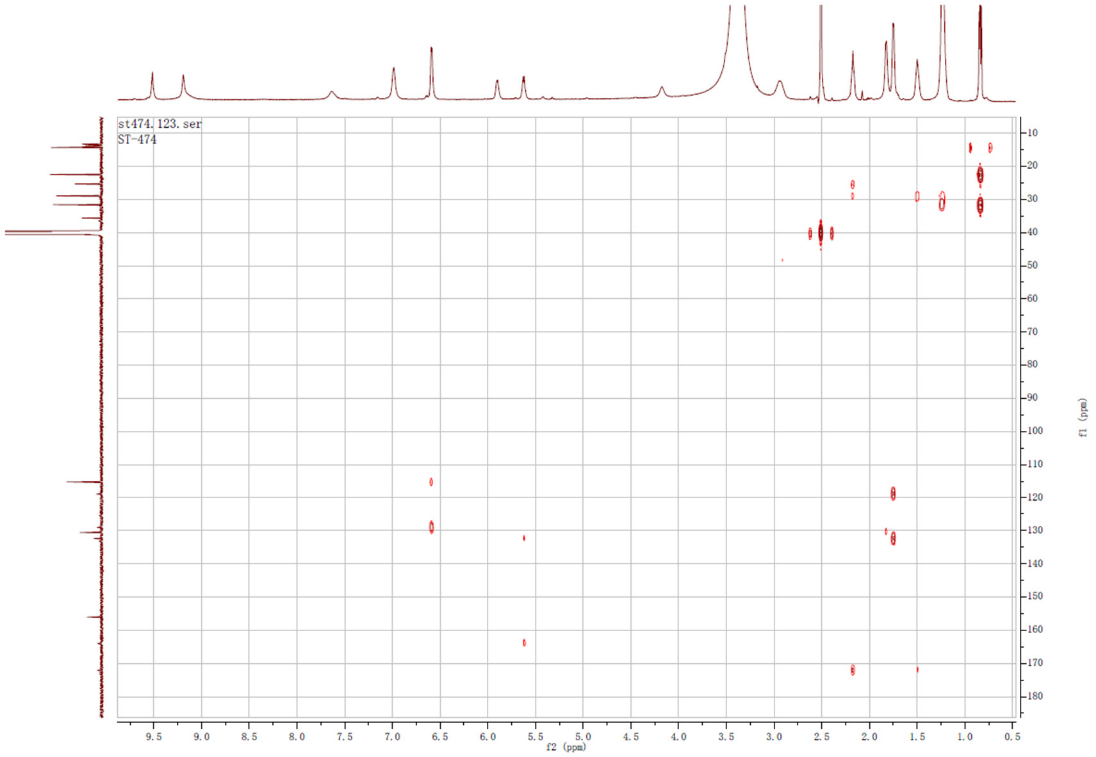

Figure S59. HMBC spectrum of haereogladiodin B (**10**) in DMSO-d_6
